# Chemical Carbonylation of Arginine in Peptides and Proteins

**DOI:** 10.1021/jacs.4c14476

**Published:** 2025-03-15

**Authors:** Lyndsey Prosser, Benjamin Emenike, Pinki Sihag, Rajendra Shirke, Monika Raj

**Affiliations:** Department of Chemistry, Emory University, Atlanta, Georgia 30322, United States

## Abstract

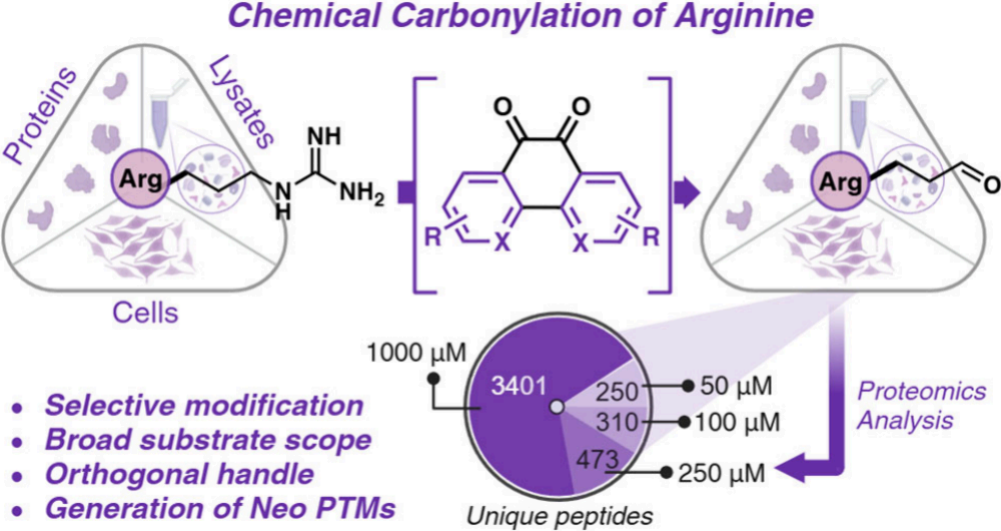

The chemoselective incorporation of arginine carbonylation post-translational modification (PTM) within proteins represents an underexplored frontier. This is largely due to the poor nucleophilicity and resistance to chemical oxidation of arginine. Drawing inspiration from the metal catalyzed oxidation (MCO) processes of arginine, we introduce a chemical methodology aimed at generating glutamate-5-semialdehyde from arginine residues within peptides and proteins. This innovative chemical approach capitalizes on the inherent weak nucleophilicity and oxidative properties of arginine. We also demonstrate the application of this strategy to selectively introduce both natural and unnatural post-translational modifications (PTMs) in a targeted manner. Our chemical approach offers a rapid, robust, and highly selective technique, facilitating chemoproteomic profiling of arginine sites prone to forming glutamate-5-semialdehyde PTM within the human proteome. Additionally, this methodology serves as a versatile platform for uncovering microenvironments that are susceptible to undergoing arginine carbonylation PTM, enabling the understanding of the effect of oxidative stress on arginine in proteins and the impact of these PTMs on cellular processes.

## Introduction

Protein carbonylation, the oxidation of amino acids to carbonyl moieties, is a common indicator of oxidative stress and is associated with numerous diseases such as Alzheimer’s disease, chronic kidney disease, cancer, diabetes, and fibrosis.^1−4^ Consequently, the ability to understand the role of protein carbonylation in these disease states is imperative. A notable focus in protein carbonylation research is the ability to create model systems that facilitate the determination of protein environments that are more susceptible to carbonylation.^5−9^

**Figure 1:**
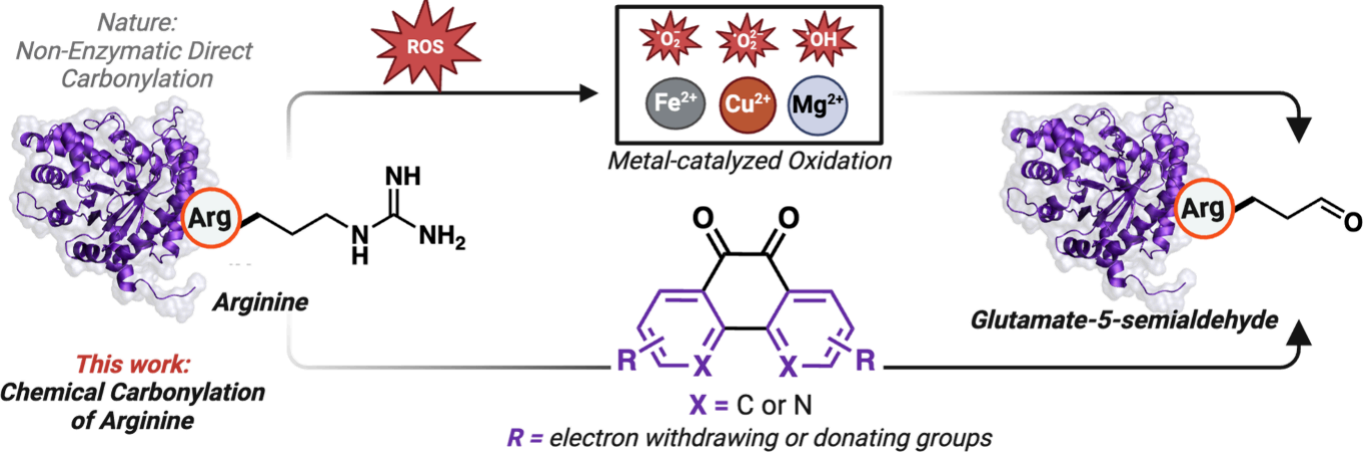
Platform for the selective carbonylation of arginine. Nonenzymatic oxidation of the guanidine group of arginine via metal-catalyzed oxidation (MCO) in the presence of ROS and reduced metal ions to generate glutamate-5-semialdehyde. This work: Selective carbonylation of arginine to generate glutamate-5-semialdehyde through the use of a metal-free chemical approach utilizing 9,10-phenanthrenequinone analogs. Created with BioRender.com, released under a Creative Commons Attribution-NonCommercial-NoDerivs 4.0 International license (Agreement number: TU27EWWM6M).

Arginine is one of the most common sites for protein carbonylation and is abundant in proteins, comprising approximately 3.9% of the human proteome.^10,11^ In nature, protein carbonylation occurs by a nonenzymatic pathway including direct reactive oxygen species (ROS) attack on arginine or metal-catalyzed oxidation (MCO) of arginine in the presence of ROS and reduced metal ions to generate glutamate-5-semialdehyde,^12−15^ a major product of arginine carbonylation (Figure 1). Despite the plethora of methods for detecting arginine carbonylation, there is no selective chemical method for incorporating glutamate-5-semialdehyde into peptides and proteins. Developing such methods would facilitate the identification of arginine residues on proteins that are more prone to carbonylation and elucidate their role in modulating interactions with other proteins.^16−18^

The chemical incorporation of arginine carbonylation has been challenging due to the low nucleophilicity and resistance of its guanidine group to chemical oxidation.^19−21^ Inspired by the facile oxidation of arginine by direct MCO in the presence of ROS, and the reaction of guanidine with dicarbonyl compounds,^22−25^ we introduce a chemical advancement utilizing 9,10-phenanthrenequinone for the carbonylation of arginine residues in peptides and proteins to glutamate-5-semialdehyde (Figure 1). This method facilitates rapid protein diversification, marking a significant step in the selective incorporation of arginine carbonylation within complex biological environments. The application of this novel arginine carbonylation strategy has led to the identification of new protein targets that are more susceptible to arginine oxidation in the human proteome. Additionally, our chemoproteomics exploration revealed sequence motifs of arginine residues that are prone to carbonylation within the human proteome. We also demonstrated the utility of this chemical strategy in generating cellular models of arginine carbonylation, providing a chemical platform for understanding its effect on cellular components. Moreover, this method directly introduces reactive aldehyde groups onto peptides and proteins, facilitating functionalization through aldehyde-specific reactions such as hydroxylamine and reductive amination.

## Results and Discussion

### Development of Chemical Carbonylation of Arginine

**Figure 2:**
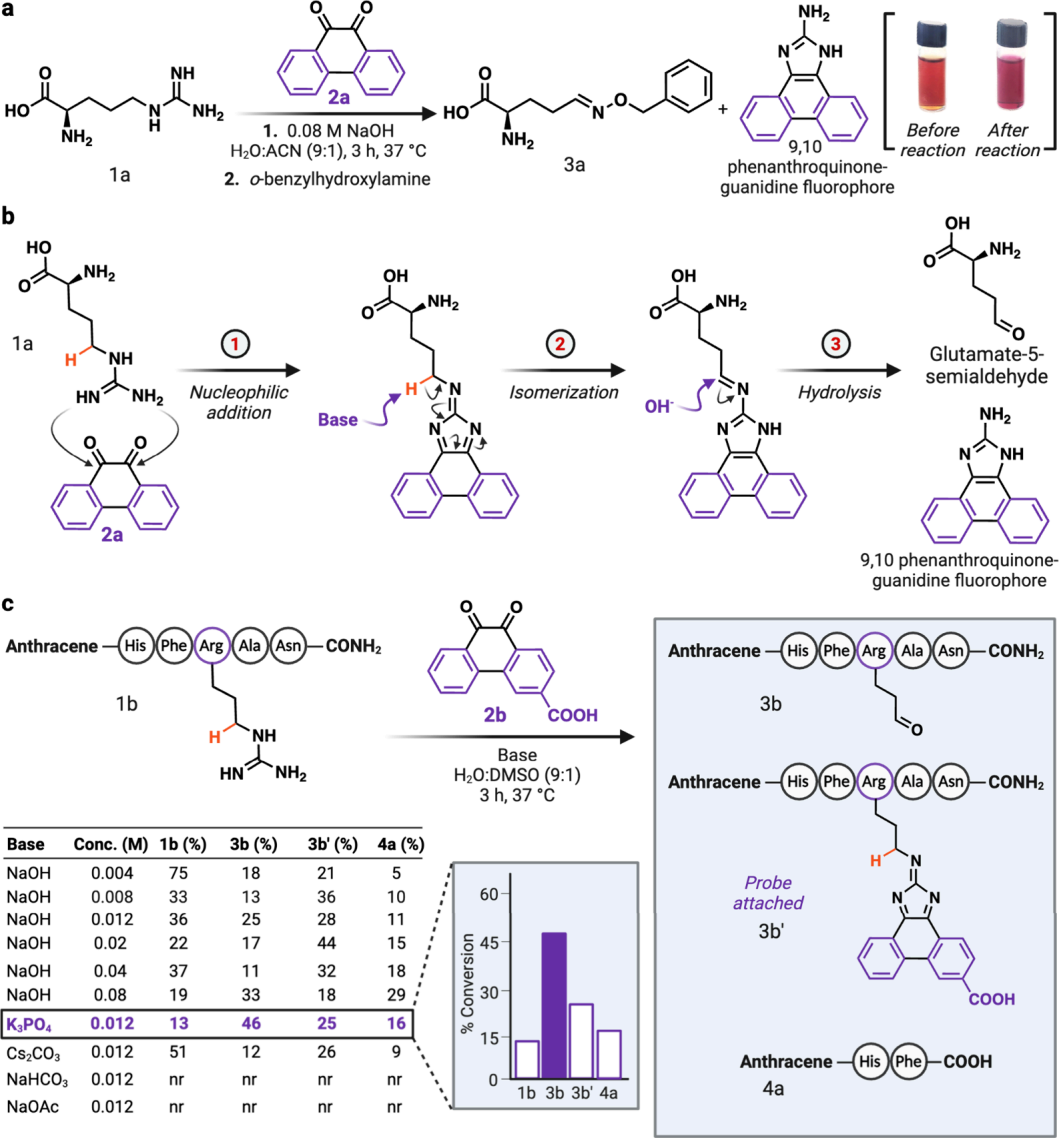
Development and optimization of arginine carbonylation reaction conditions. (a) Formation of glutamate-5-semialdehyde by the reaction of 9,10-Phenanthrenequinone with l-Arg. (b) Proposed Mechanistic pathway for the synthesis of glutamate-5-semialdehyde product from Arg. (c) Optimization of reaction conditions on a model peptide **1b** with **2b** to obtain high conversion to glutamate-5-semialdehyde **3b** by varying bases. Created with BioRender.com, released under a Creative Commons Attribution-NonCommercial-NoDerivs 4.0 International license (Agreement number: FV26S9AK33)

Drawing inspiration from the non-enzymatic metal-catalyzed oxidation of arginine^25−28^ and our previous work on carboxypeptidase B mimic for the selective cleavage of C-terminal arginine,^29^ our current study investigates arginine carbonylation using 9,10-phenanthrenequinone derivatives to selectively modify arginine (Arg) residues in peptides and proteins to glutamate-5-semialdehyde. Commencing with the treatment of l-Arg **1a** with 9,10- phenanthrenequinone **2a** in aqueous solution H_2_O:ACN (9:1) and 0.08 M NaOH at 37 °C for 3 h, we observed the formation of glutamate-5-semialdehyde. The aldehyde was converted to oxime **3a** through reaction with benzyl hydroxylamine and subsequently isolated (Figure 2a, Figure S1). The reaction also produced a 9,10-phenanthrenequinone-guanidine fluorophore, causing the reaction mixture to turn purple (Figure 2a, Figure S1). Subsequently, we carried out the reaction with a small molecule benzyl guanidine with 9,10-phenanthrenequinone analogue **2b** in ACN: H_2_O (9:1) and 0.08 M NaOH at 37 °C for 3 h and observed the formation of benzaldehyde (70% yield) along with the formation of 9,10-phenanthrenequinone-guanidine fluorophore (Figure S2). Our proposed reaction mechanism for the guanidine carbonylation involves nucleophilic addition of arginine to diketones of 9,10-phenanthrenequinone, generating imines (Figure 2b). We postulate that the aromatic rings of 9,10-phenanthrenequinone enhance conjugation, consequently lowering the p*K*_a_ of the alpha-carbon proton adjacent to guanidine. This decrease in p*K*_a_ leads to deprotonation of the alpha-carbon proton, followed by isomerization to create a new imine that is hydrolyzed to create the 9,10-phenanthrenequinone-guanidine fluorophore and glutamate-5-semialdehyde.^30,31^

To validate this hypothesis and adapt this reaction for carbonylation of Arg on proteins, we investigated the water-soluble 9,10-phenanthrenequinone-3-carboxylic acid **2b** under aqueous conditions H_2_O:DMSO (9:1) using a model peptide anthracene-HFRAN **1b** (Figure 2c, Figure S3). The peptide was labeled with anthracene to increase its visibility on HPLC at 220 nm due to the high absorbance of the 9,10-phenanthrenequinone and the corresponding fluorophore obtained by the reaction of 9,10-phenanthrenequinone with the Arg side chain. Initially, by varying the amounts of sodium hydroxide as a base (from 0.004 to 0.08 M), we observed the formation of the desired glutamate-5-semialdehyde product anthracene-HFR(CHO)AN **3b**, alongside the formation of the probe-attached anthracene-HFR(PQ)AN product **3b′** and the peptide cleavage product **4a**. We propose that the cleavage product arises from the formation of a pyrollinium-like intermediate between the amide backbone chain and the aldehyde handle obtained from the Arg side chain, leading to hydrolysis of the backbone amide (Figure S3). To enhance conversion to the desired glutamate-5-semialdehyde peptide product **3b**, we tested various bases such as K_3_PO_4_, Cs_2_CO_3_, NaHCO_3_, and NaOAc, and found that the maximum conversion (46%) occurred with 0.012 M K_3_PO_4_ (Figure 2c, Figure S3, Table S1). This data highlights the pivotal role of the base in deprotonating the proton of the α carbon adjacent to the guanidine ring. Surprisingly, we did not observe the significant cleavage of the C-terminal Arg residue (<13%) under the reaction conditions which is in contrast to the previous work (Figure S3).^29^ This might be due to the use of a lower equivalent of the probe, less basic conditions, and lower amounts of organic solvent under the reaction conditions.

**Figure 3:**
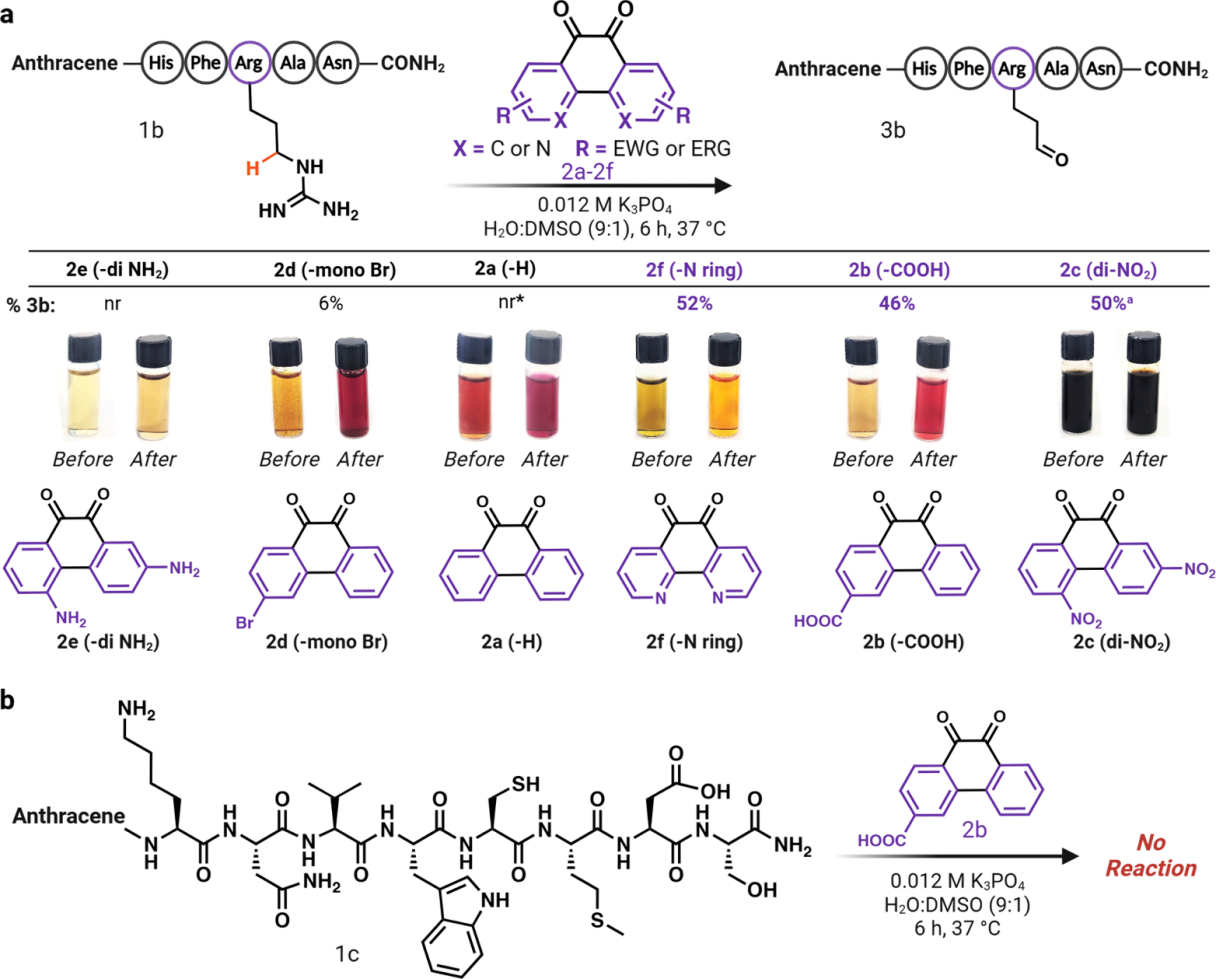
Evaluation of substituted 9,10-phenanthrenequinone analogs on the arginine carbonylation reaction (a) Screening of varying 9,10-phenanthrenequinone analogs with EWG and ERG for the modification of Arg to aldehyde on a model peptide **1b**. nr = no reaction, nr* = no reaction under optimized conditions but reaction condition was modified to show the generation of the fluorophore byproduct, a = reaction was evaluated using 0.01 M NaOH as a base as reaction decomposed in K_3_PO_4_due to high reactivity of probe. (b) Chemoselectivity evaluation of the arginine carbonylation reaction with a peptide **1c** containing various reactive amino acids. No modification of peptide **1c** was observed with **2b** under the optimized reaction conditions. Created with BioRender.com, released under a Creative Commons Attribution-NonCommercial-NoDerivs 4.0 International license (Agreement number: RU26STO6IB).

To further enhance the conversion to the desired glutamate-5-semialdehyde product **3b** within the peptide **1b**, we examined analogs of 9,10-phenanthrenequinone (**2a**–**2f**) bearing electron-withdrawing (EWG) groups (mono-COOH; **2b**, di-NO_2_; **2c**) and electron-releasing (ERG) groups (mono-Br; **2d**, di-NH_2_; **2e**), including the phenanthroline derivative **2f**, where the CH groups in the hydrocarbon at positions 4 and 5 of 9,10-phenanthrenequinone are replaced with nitrogen atoms (Figure 3a). Probes **2c** and **2e** were synthesized in a multistep process (see Figure S4). These analogs were tested by adding one equivalent to a model peptide anthracene-HFRAN **1b** in a 9:1 H_2_O:DMSO solution with 0.012 M of K_3_PO_4_ at 37 °C for 6 h, followed by HPLC and MS analysis (Figure 3a, Figure S5). Interestingly, we observed the visible change in the color of the reaction mixtures (**2a**–**2f**), plausibly due to the formation of 9,10-phenanthrenequinone-guanidine fluorophore during the hydrolysis of imine that generates glutamate-5-semialdehyde **3b** (Figure 3a, Figure S6).

The experiments demonstrated an increased conversion to the glutamate-5-semialdehyde product anthracene-HFR(CHO)AN **3b** (52%) with 9,10-phenanthroline-5,6-dione **2f**, which further increased to 71% in 10 h (Figure S5, Table S2), compared to no conversion with the unsubstituted 9,10-phenanthrenequinone **2a** and 6% conversion with mono-Br (**2d**) derivative due to their poor solubility in aqueous conditions (Figure 3a, Figure S5). Evaluation of 9,10-phenanthrenequinone-3-carboxylic acid **2b** led to decent conversion to the desired glutamate-5-semialdehyde product anthracene-HFR(CHO)AN **3b** (46%) (Figure 3a, Figure S5). Interestingly, screening of phenanthrenequinone analogue **2c** with two nitro substituents led to the immediate degradation under the reaction conditions plausibly due to elevated reactivity of the probe. However, evaluation of the di-nitro probe **2c** in 0.01 M NaOH hydroxide led to 50% formation of **3b** in 3 h. Taken together, these results suggest an increase in the reactivity of phenanthrenequinone analogs (**2b**, **2c**, and **2f**) substituted with electron-withdrawing groups (EWGs) toward arginine. To rationalize the observed reactivity trends of 9,10-phenanthrenequinone analogs, computational calculations of the electrostatic potential (ESP) map of these analogs were performed. Analysis of the results showed increasing electropositive characteristic of 9,10-phenanthrenequinone analogs, with the maximum electrophilicity observed for electron withdrawing analogs (**2b**, **2c**, and **2f**) (Figure 3b, Figure S7). These observations suggest an increased reactivity of the diketone moiety in EWG analogs with the guanidinium group of arginine. These computational results corroborate with our experimental findings, thus highlighting an increased reactivity of electron withdrawing analogs over electron releasing substituents (Figure 3b, Figure S7).

### Chemoselectivity Studies of Carbonylation Reaction

Chemoselectivity studies unveiled the specificity of the carbonylation reaction in producing aldehyde products exclusively with Arg, as evidenced by the reaction of **2b** with peptides **1c**, anthracene-KNVWCMDS, and **1d**, anthracene-HRW, containing different reactive amino acids with high nucleophilicity and oxidation potential (K, N, W, C, M, D, S) as compared to Arg (Figure 3b, Figure S8). 58% conversion to the corresponding glutamate-5-semialdehyde **3d** anthracene-HRW was observed under the reaction conditions as analyzed by HPLC and MS (Figure S9). We did not observe any oxidation of Met or Cys under the reaction conditions. Overall, these comprehensive experiments display this chemistry as an efficient and selective method for arginine carbonylation.

### Selective Chemical Carbonylation of Arginine in Proteins

**Figure 4:**
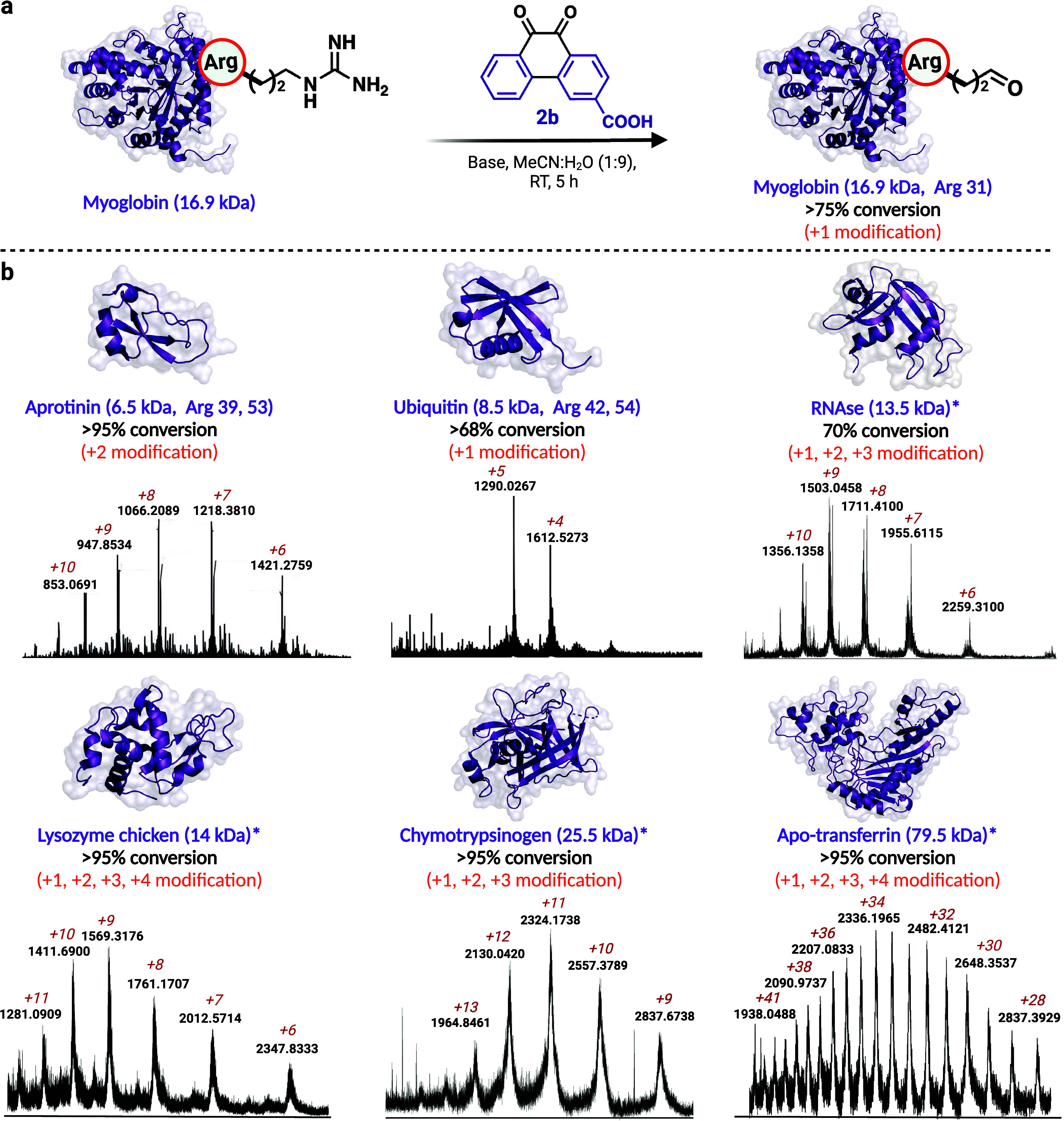
Chemical carbonylation of arginine in various proteins (6.5 kDa–80 kDa). (a) Selective modification of arginine of myoglobin to glutamate-5-semialdehyde using probe **2b**. The % conversion to modified myoglobin was analyzed by MS. Carbonylation reaction on myoglobin using 480 μM carboxylate-substituted 9,10-phenanthrenequinone **2b** yielded a homogeneously modified glutamate-5-semialdehyde protein. MS/MS analysis identified R31 as a site of modification. (b) Carbonylation reaction for the modification of arginine in different proteins with **2b** and **2c**. The % conversion to modified proteins was analyzed by MS and the sites of modifications were determined by MS/MS analysis. * = proteins modified using probe **2c**. Created with BioRender.com, released under a Creative Commons Attribution-NonCommercial-NoDerivs 4.0 International license (Agreement number: BP27EX18EK).

Expanding our investigation to protein carbonylation, we began optimization studies with myoglobin (Mb) containing two arginine residues using the water-soluble electron-withdrawing probe **2b**. Initial treatment of myoglobin (240 μM) with **2b** (480 μM, 2 equiv) in a 1:9 ACN:H_2_O in 0.08 M NaOH primarily modified one Arg to glutamate-5-semialdehyde (75% conversion) within 5 h, as confirmed by LCMS (Figure 4a, Figure S10). A similar observation was made when the reaction was evaluated using 13 mM organic base (DBU), yielding 72% conversion to a single modification of arginine (Figure S10). Subsequent MS/MS analysis of modified myoglobin identified R31 as the preferred modification site (Figure 4a, Figure S10). Although two of the arginines present in myoglobin are surface-exposed, only one was preferentially carbonylated, suggesting a differential propensity of various arginine residues to undergo oxidation.

Using 9,10-phenanthrenequinone analogue **2b** and 0.08 M NaOH, reactions were conducted with additional proteins, including aprotinin and ubiquitin. Aprotinin, with six arginine residues and three disulfides, exhibited full conversion to two Arg carbonylations (Figure 4b, Figure S10). MS/MS analysis revealed R39 and R53 as sites of modifications on aprotinin. A 68% conversion of Arg to glutamate-5-semialdehyde was observed with ubiquitin, resulting primarily in a single modification of one arginine, along with minor modification of the second arginine out of the four arginines present in ubiquitin (Figure 4b, Figure S10). Subsequent MS/MS analysis of modified and digested ubiquitin identified R42 as the preferred site of modification along with the minor modification of R54 (Figure 4b, Figure S10). To further demonstrate the scope of the electron withdrawing analogs of 9,10-phenanthrenequinone, we utilized probe **2c** in 0.01 M NaOH for the carbonylation of additional protein substrates with varying 3D structures and molecular weights (14.3–80 kDa) such as RNase, lysozyme chicken, chymotrypsinogen, and apo-transferrin. These experiments exhibited 70 to >95% modification of arginine to glutamate-5-semialdehyde with excellent selectivity, even at low concentrations of proteins (70 μM) (Figure 4b, Figure S10). The analysis of the intact mass spectra of carbonylated proteins showed no breakage of the disulfides, further confirming the highly chemoselective nature of our carbonylation platform. By adjusting the reaction conditions, we demonstrated that homogeneous modification to a single aldehyde product can be achieved on varying proteins of different sizes and molecular weights such as myoglobin, ubiquitin, aprotinin, and cytochrome C (Figure S10). Taken together, these results demonstrate the robustness of our chemical platform for carbonylation of a wide range of protein substrates.

### Arginine Carbonylation Generates De Novo Intramolecular Imines and Hydrates

**Figure 5:**
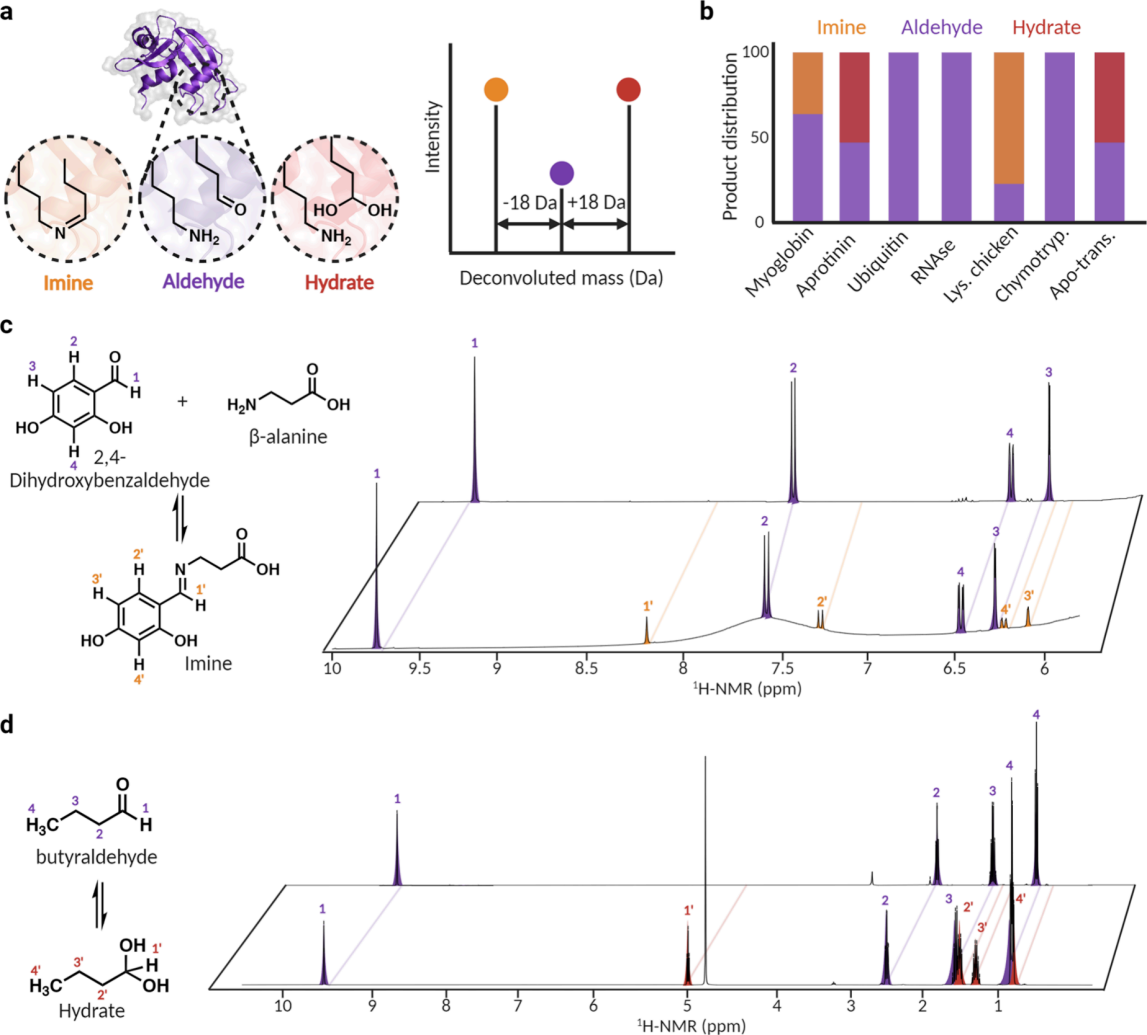
De novo intracross-linking of carbonylated proteins. (a) Schematic showing the formation of the de novo formation of Schiff base (−18 Da) between a lysine residue with glutamate-5-semialdehyde modified protein and the formation of a hydrate (+18 Da) on the carbonyl of glutamate-5-semialdehyde. (b) Product distribution (Schiff base, aldehyde, hydrate) of the carbonylated protein. Myoglobin (39% imine, 61% aldehyde), aprotinin (50% aldehyde, 50% hydrate), ubiquitin (100% aldehyde), RNase (100% aldehyde), lysozyme chicken (75% imine, 25% aldehyde), chymotrypsinogen (100% aldehyde), and apo-transferrin (50% aldehyde, 50% hydrate). It should be emphasized that these data do not allow direct identification of lysines forming imines with glutamate-5-semialdehyde. (c) Incubation of water-soluble 2,4-dihydroxybenzaldehye with β-alanine in a deuterated DMSO-phosphate buffer (pH 12) mixture for 2 h followed by ^1^H NMR analysis. Results revealed the formation of characteristic imine protons not observed in control sample containing 2,4-dihydroxybenzaldehye without β-alanine. (d) Incubation of butyraldehyde in 20 mM Phosphate buffer (pH 12) for 2 h followed by ^1^H NMR analysis. Formation of characteristic hydrate protons were observed that were absent in DMSO control. Created with BioRender.com, released under a Creative Commons Attribution-NonCommercial-NoDerivs 4.0 International license (Agreement number: FI27F1PN4C).

Protein carbonylation has been reported to activate various signaling pathways associated with diseases. This is primarily attributed to the alteration of the interactions and structures resulting from the conversion of the positively charged arginine residue to a neutral and reactive aldehyde group. Investigating these changes in interactions due to selective chemical carbonylation of arginine is crucial for understanding its implications in cellular processes and disease mechanisms. The analysis of chemical carbonylation of arginine in various proteins led to an interesting observation: the formation of imines (−18 Da) and hydrate (+18 Da) derived from glutamate-5-semialdehyde product (Figure 5a, 5b, Figure S10). This observation suggests a plausible formation of Schiff base between glutamate-5-semialdehyde and a nearby lysine residue and the hydration of the carbonyl group of glutamate-5-semialdehyde. To demonstrate the formation of imine and hydrate glutamate-5-semialdehyde, we carried out NMR investigations of small molecule mimics of aldehydes and amines.

We incubated water-soluble 2,4-dihydroxybenzaldehye with β-alanine in deuterated DMSO-phosphate buffer) mixture for 2 h. ^1^H NMR analysis revealed the formation of characteristic imine protons that were absent in 2,4-dihydroxybenzaldehye without β-alanine (Figure 5c, Figure S10). Additionally, the incubation of butyraldehyde in 20 mM phosphate buffer for 2 h led to the appearance of characteristic hydrate protons that were absent in DMSO control (Figure 5d, Figure S10). Taken together, these observations highlight the potential intra- and inter-cross-linking of lysine residues with glutamate-5-semialdehyde within carbonylated proteins.

### Chemical Carbonylation for Installation of Fluorophores in Proteins and Cell Lysates

**Figure 6:**
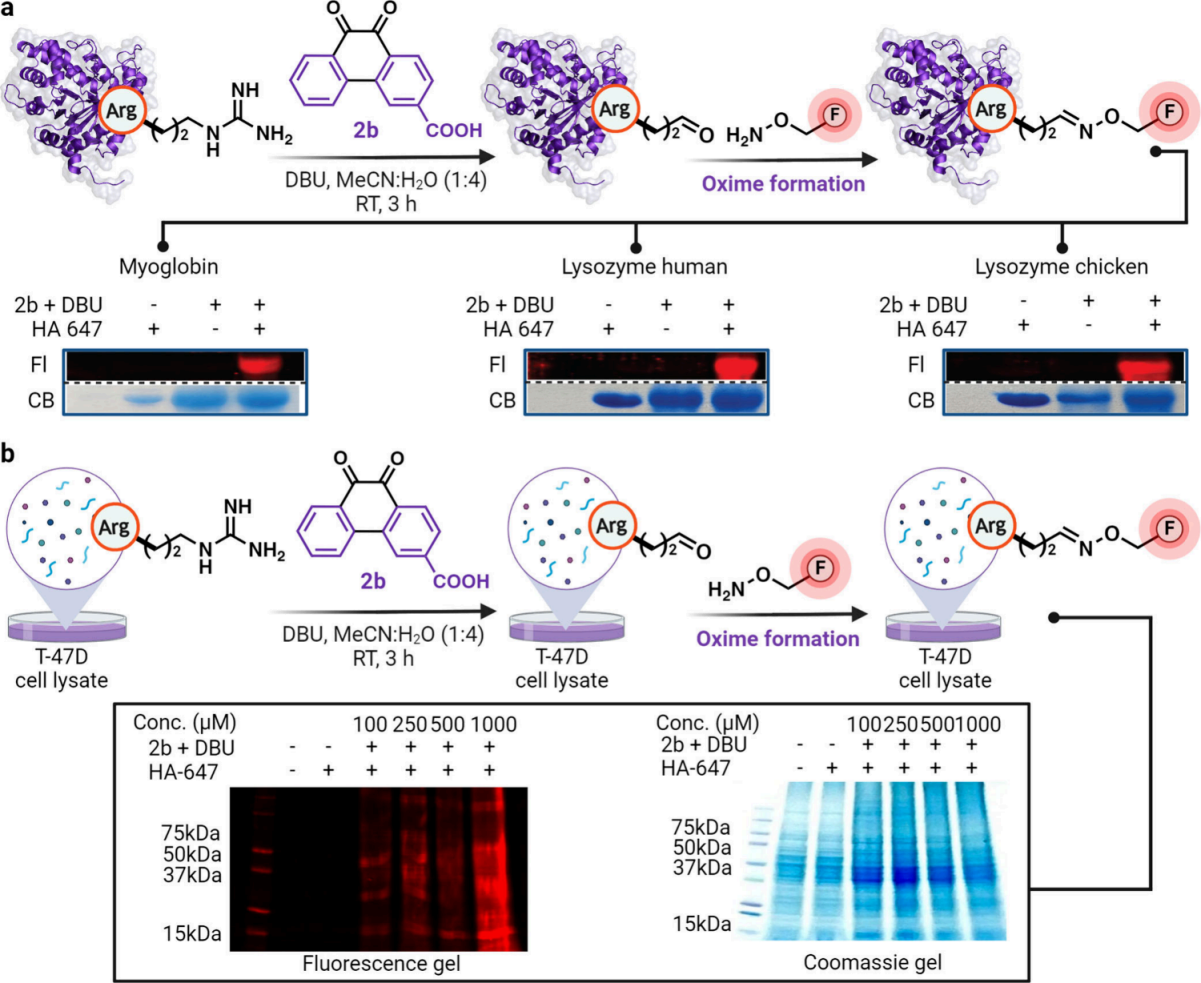
Fluorescence tagging of carbonylated arginine in proteins and the proteome. (a) Carbonylation of proteins (myoglobin, lysozyme human, and lysozyme chicken) followed by oxime formation with hydroxylamine-647 fluorophore and analysis by in-gel fluorescence. (b) Dose-dependent labeling of a complex cell lysate in the presence of a carbonylating reagent as analyzed by in-gel fluorescence. Created with BioRender.com, released under a Creative Commons Attribution-NonCommercial-NoDerivs 4.0 International license (Agreement number: TX26S9AZFZ).

The ability to generate glutamate-5-semialdehyde also allows for further diversification of the generated aldehyde modalities with affinity tags and fluorophores. To assess this capability, we functionalized carbonylated proteins (myoglobin, human lysozyme, and chicken lysozyme) with hydroxylamine-647 fluorophores and analyzed them using in-gel fluorescence (Figure 6a, Figure S11). The results distinctly displayed fluorescent labeling of carbonylated proteins (Figure 6a, lane 3, Figure S11), with no fluorescence signal observed without treatment with 9,10-phenanthrenequinone-3-carboxylic acid **2b** under reaction conditions (Figure 6a, lane 1).

Encouraged by the high labeling efficiency of this method in individual proteins, we showcased its applicability in the selective labeling of native proteins within complex systems such as the cell lysate. To achieve this, we incubated cell lysate obtained from prostate cancer cells (T-47D) with **2b** and DBU in a dose-dependent manner (100 μM to 1 mM) for 3 h, followed by the attachment of hydroxylamine-647 dye. In-gel fluorescence analysis clearly revealed extensive labeling of Arg on proteins in the cell lysate mixture in the presence of all of the components (Figure 6b, Figure S12). No labeling was observed in the control experiments without the carbonylation reagent 2b (lanes 1–2, Figure 6b). The ability to selectively introduce arginine carbonylation in a complex mixture such as cell lysate supports the potential application of this chemical approach for evaluating protein sites that are susceptible to oxidation.

### Arginine Carbonylation Chemoproteomic Profiling

**Figure 7:**
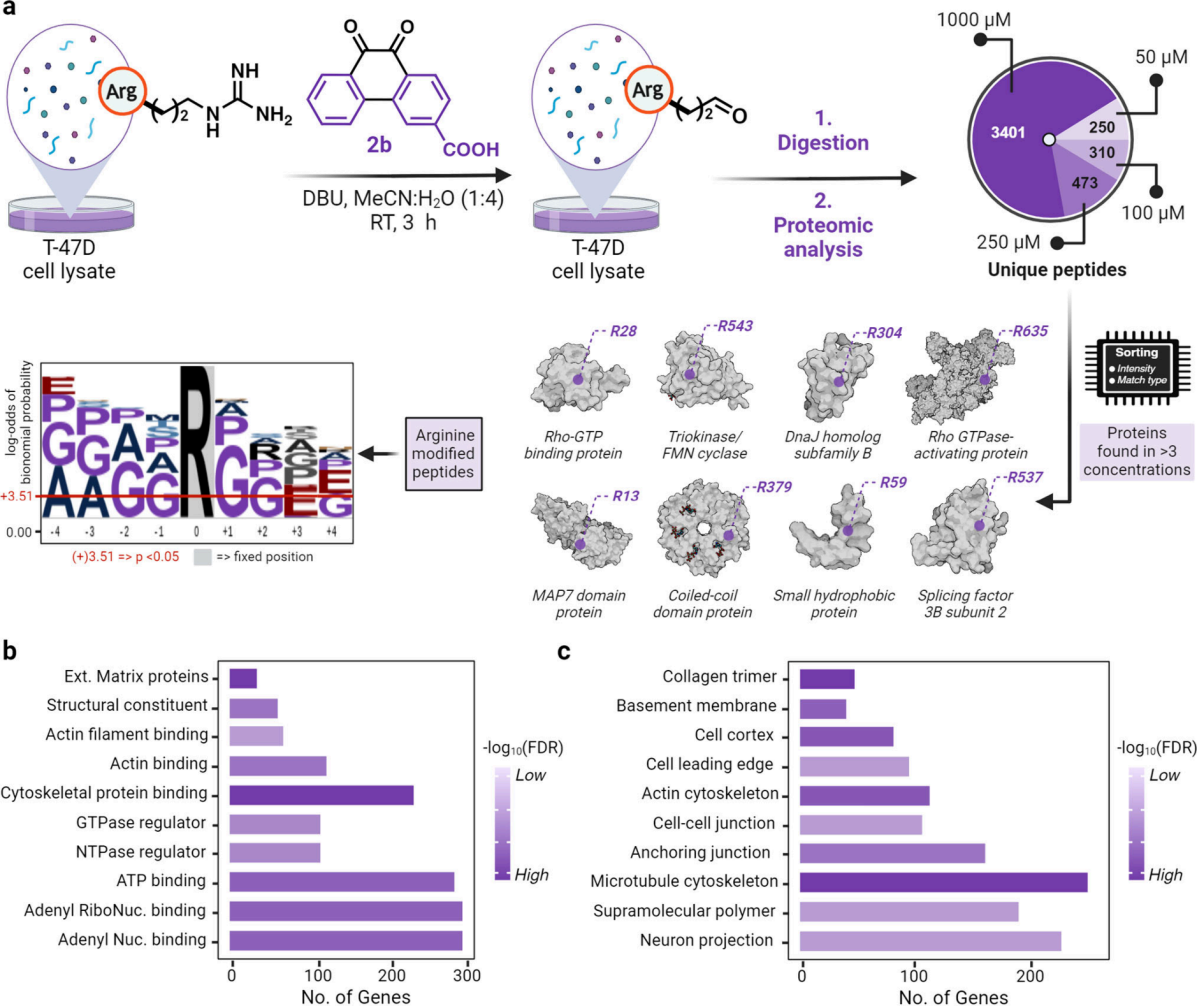
Chemoproteomics profiling of arginine residues prone to oxidation in cell lysates. (a) Carbonylated arginine profiling through treatment of T-47D breast cancer cell lysate with low (50 μM), medium (100 μM), high (250 μM), and superhigh doses (1000 μM) of carboxylate-substituted 9,10-phenanthrenequinone **2b** in 1:4 ACN: water, followed by Glu-C digestion for LC-MS/MS analysis. Analysis of results identified the modification of 250 unique peptides at 50 μM, 310 unique peptides at 100 μM, 473 unique peptides at 250 μM, and 3401 unique peptides at 1000 μM. Seventeen peptides were observed in the control sample (0 μM). Unique peptides for all concentrations were normalized using the control sample (0 μM). Analysis of probe **2b** modified peptides to identify proteins with hyperreactive arginine residues led to the Identification of 8 proteins with arginine sites susceptible to carbonylation. Sequence motif analysis of carbonylated arginine sites clearly identified a significant distribution of flexible and turn inducing residues such as glycine, proline, and alanine followed by glutamic acid. (b) Gene ontology analysis of modified proteins clearly shows a significant modification of regulatory and binding proteins. (c) Functional categorization of modified proteins showed a broad diversity of modified proteins, with a significant modification of −log10(FDR) > 14 for structural proteins. Created with BioRender.com, released under a Creative Commons Attribution-NonCommercial-NoDerivs 4.0 International license (Agreement number: QG26S9ABP7).

To further demonstrate the application of our novel chemical strategy for uncovering arginine sites in the proteome that are susceptible to carbonylation, we incubated T-47D cell lysates with varying concentrations of carboxylate-substituted 9,10-phenanthrenequinone **2b** (50 μM to 1 mM), followed by digestion and LC-MS/MS analysis. Analysis revealed a dose-dependent conversion of Arg to glutamate-5-semialdehyde in the proteome (250 unique peptides at 50 μM; 310 unique peptides at 100 μM; 473 unique peptides at 250 μM; and 3401 unique peptides at 1 mM; Figure 7a, Figure S13). This result demonstrates the increasing generation of arginine carbonylation products under increased oxidative stress conditions.

A comparison of the carbonylated proteins identified using phenanthrenequinone **2b** with previously arginine-carbonylated proteins observed in cells under oxidative stress modified by metal-catalyzed oxidation (MCO) revealed an overlap of approximately ∼30% (43 out of 144 proteins).^32^ It is important to note, however, that the cell lines and experimental conditions utilized in these studies are distinct, which may account for the differences in the identified proteins.

We emphasize that the major means of generating arginine carbonylation in cells is through metal catalyzed oxidations (MCO) and not enzyme catalysis. Since the mechanism of arginine oxidation in cells under oxidative stress via metal-catalyzed oxidation (MCO) parallels the mechanism employed by our phenanthrenequinone probe, it is reasonable to propose that the carbonylated sites identified using our probe serve as markers for arginine residues particularly prone to carbonylation.

To confirm the correlation between our method and the metal catalyzed oxidation (MCO) reaction, we attempted to modify proteins under MCO reaction conditions (Figure S13), but we did not observe the formation of Arg-to-aldehyde conversions under any of the reported conditions for modifying Arg to aldehydes in cells. This lack of observable modification underscores the novelty and significance of our study, which represents the first reported method to achieve this selective modification.

Further, the sorting of carbonylated arginine sites in at least 3 concentrations identified 8 proteins possessing arginine sites with an elevated propensity to undergo carbonylation. Most of the modified proteins possess enzymatic, regulatory, and structural functions (Figure 7a). The sequence motif analysis of carbonylated arginine sites clearly identified significant distribution of flexible and turn inducing residues such as glycine, proline, and alanine followed by glutamic acid (Figure 7a, Figure S14).^33^ We hypothesize that these arginine residues are more prone to carbonylation because the neighboring flexible and turn inducing residues enable glutamic acid to abstract the proton from the Arg thus activating them for the reaction with diketones. This data correlates with previous report on the sequence motif studies of carbonylation sites within the proteome.^34^

Gene ontology analysis revealed the significant enrichment of carbonylated arginine residues within numerous proteins exhibiting enzymatic, regulatory and structural functions (Figures 7b,c and S15).^35^ These observations highlight the encompassing effect of arginine carbonylation on various biological functions and processes. Interestingly, these observations closely correlate with previous reports on the effect of protein carbonylation on cytoskeletal proteins, extracellular matrix proteins, in addition to ATP binding proteins.^36−40^

### Carbonylation of Proteins inside Cells

**Figure 8:**
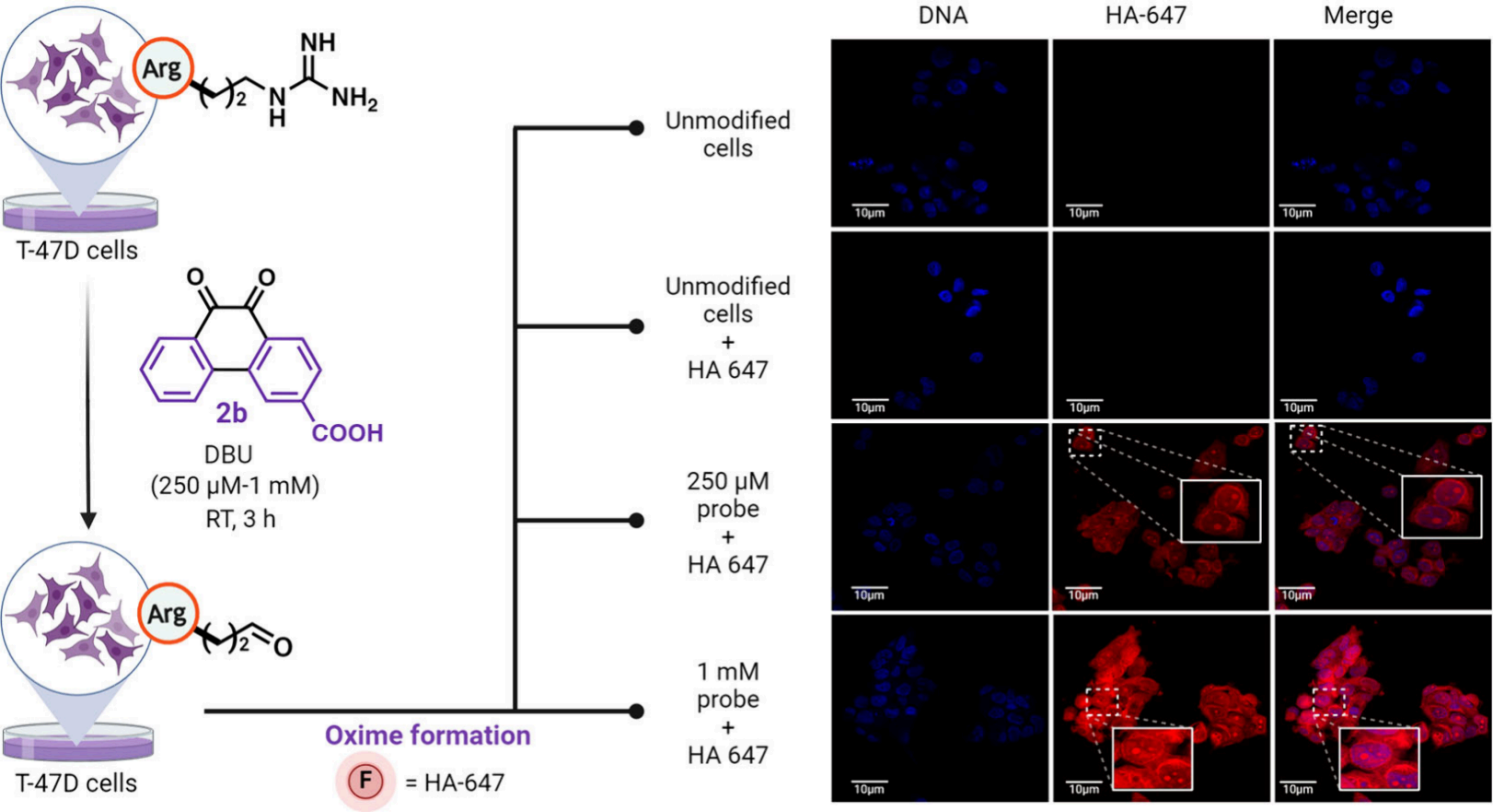
Carbonylation of proteins inside cells. Confocal imaging of fixed T-47D cells after treatment with carbonylation reagent **2b** in a dose-dependent manner and labeling with hydroxylamine HA-647 dye. Identification of protein localization in modified cells. Created with BioRender.com, released under a Creative Commons Attribution-NonCommercial-NoDerivs 4.0 International license (Agreement number: JA26STUNA7).

Lastly, we assessed the ability to modify Arg-containing proteins within cells. This application holds immense potential for advancing our understanding of hotspot regions within cells that are prone to carbonylation. Motivated by this, we conducted confocal microscopy imaging of **2b**-modified fixed T-47D cells at concentrations of 250 μM and 1 mM (Figure 8, Figure S16). No fluorescence signal was observed in the HA-647 channel for unmodified cells. However, a dose-dependent fluorescent intensity (250 μM and 1 mM) was observed for HA-647 cells treated with carbonylation reaction conditions (Figure 8, Figure S16). This result also identified the localization of chemically carbonylated proteins within the cell membrane, cytoplasmic, and nuclear regions, indicating the potential of this method for carbonylation of proteins within diverse spatiotemporal localizations within a cell.

### Carbonylation Mediated Installation of Post-translational Modifications

**Figure 9:**
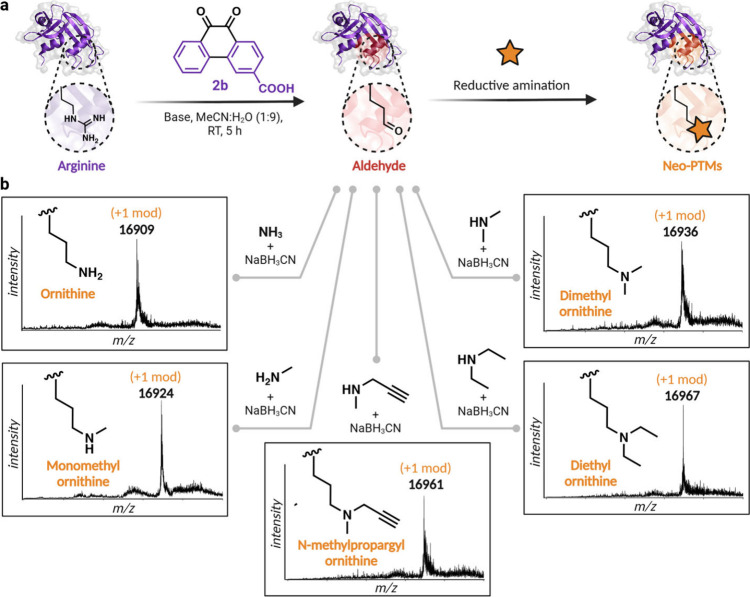
(a) Schematic showing the incorporation of various post-translational modifications through carbonylation of arginine and labeling of the generated aldehyde moiety. (b) Incorporation of various PTMs was by reductive amination of carbonylated arginine. Ornithine generated with ammonia, monomethylation of ornithine generated with *N*-methylamine, dimethylation of ornithine with *N*,*N*-dimethylamine, dimethylation of ornithine with *N*,*N*-diethylamine, and *N*-methylpropargylation of ornithine with *N*-methylpropargylamine. Created with BioRender.com, released under a Creative Commons Attribution-NonCommercial-NoDerivs 4.0 International license (Agreement number: YJ27PNAR65).

To showcase the broader applicability of the carbonylation platform, we proposed utilizing the aldehyde moiety generated through carbonylation as a reactive handle for incorporating neo-post-translational modifications (PTMs) onto proteins. Post-translational modifications (PTMs) of proteins enhance their structural and functional diversity beyond what is encoded by the genetic code.^41−43^ However, the broad range of chemically plausible side chains, both natural and unnatural, is difficult to access through conventional methods. In this study, we utilize a carbonylation strategy that converts arginine residues in proteins into aldehydes to serve as a flexible platform for introducing a wide variety of PTMs, both natural and synthetic. To demonstrate this, we modified myoglobin using our optimized reaction conditions with probe **2b** to introduce a single aldehyde handle on the protein. Homogeneously modified myoglobin was then subjected to reductive amination conditions (Figure 9a, Figure S17). By employing ammonia, *N*-methylamine, *N*,*N*-dimethyl amine, *N*-methyl propargylamine, and *N*,*N*-diethyl amine, we successfully generated ornithine and incorporated diverse PTMs on the side chain of ornithine, such as monomethylation, dimethylation, alkynylation, and diethylation (Figure 9b, Figure S17). All products were derived from a single, readily accessible aldehyde precursor on the protein through reductive amination. These modifications promote unique bonding patterns that could expand biological functions beyond those typical in natural proteins.

This approach to chemically editing arginine residues bypasses the rigid constraints of ribosomal and enzymatic processes, offering a powerful chemical tool for generating previously unattainable protein modifications. Notably, there are currently no other chemical methods available to selectively introduce these natural and unnatural PTMs onto specific amino acids on proteins in a targeted manner. Our platform represents a significant advancement in the ability to expand the functional repertoire of proteins.

## Conclusion

In summary, our pioneering work led to the development of a chemical platform for the chemoselective carbonylation of arginine within peptides and proteins. This chemical approach amalgamated the weak nucleophilicity of the guanidinium group with the low oxidative potential of the α carbon adjacent to the guanidine group, converting Arg to glutamate-5-semialdehyde. We achieved this modification by using the activated diketone substrate, 9,10-phenanthrenequinone, thereby increasing the reactivity of guanidine toward electrophilic diketones and increasing the oxidation potential of the α carbon adjacent to the guanidine group via the conjugation with phenanthrene groups. Substituting 9,10-phenanthrenequinone with EWGs further increases the reactivity of Arg to generate glutamate-5-semialdehyde. Using our chemical platform, we demonstrated the carbonylation of Arg to glutamate-5-semialdehyde in several proteins (7 examples). We further demonstrated that protein carbonylation enables the formation of new interactions such as Schiff-base and hydrate formation. The chemoproteomics exploration of cell lysate identified proteins possessing diverse biological roles as targets of arginine carbonylation under oxidative stress conditions. Furthermore, we uncovered the high abundance of flexible and turn-inducers such as proline, glycine, and alanine followed by glutamic acid surrounding arginine residues that are prone to oxidation which corroborates with known carbonylation sites. We showcased the applicability of the carbonylation platform for selectively introducing PTMs on proteins including formation of ornithine, methylated ornithine, and alkynylated ornithine from arginine. This represents a pioneering approach to achieve such modifications. To the best of our knowledge, no other method currently exists for the incorporation and chemoproteomic profiling of arginine carbonylation. Ongoing efforts in our lab aim to extend the utility of this chemical strategy for single-molecule protein sequencing of arginine using technologies such as fluorosequencing and nanopore sequencing.

## Data Availability

All data supporting the findings of this study are available within the Supporting Information.

## Supporting Information Available

Reaction procedures for the synthesis of 9,10-phenanthrenequinone analogs, procedure for the optimized carbonylation reaction, procedures for selective carbonylation of arginine in peptides and proteins to glutamate-5-semialdehyde, procedure for dose-dependent chemoproteomic profiling, procedure for tagging peptide and protein aldehydes with fluorophores, and procedure for the analysis of modified proteins by SDS PAGE and LC-MS/MS. Characterization of synthesized phenanthrenequinone analogs and the aldehyde obtained by reaction with a model small compound using ^1^H and ^13^C NMR (PDF)
Proteomics results for arginine carbonylation (XLSX)

## Abbreviations

PTMs: post-translational modifications
DFT: density functional theory
HPLC: high performance liquid chromatography
LC/MS: liquid chromatography/mass spectrometry
MCO: metal-catalyzed oxidation.
