# Supplementary material for: Chemical Carbonylation of Arginine in Peptides and Proteins
Source: J Am Chem Soc. 2025 Mar 15;147(12):10139–50. doi: 10.1021/jacs.4c14476 (PMC11951078; doi:10.1021/jacs.4c14476)
Supplement: Supplementary file 1 — ja4c14476_si_001.pdf [file ja4c14476_si_001.pdf]

Supporting Information

Chemical Carbonylation of Arginine in Peptides and Proteins

Lyndsey Prosser‡, Benjamin Emenike‡, Pinki Sihag‡, Rajendra Shirke, Monika Raj*

Table of Contents

I. General....2

II. Materials....2

III. Purification (HPLC)....2

IV. Instrumentation and sample analysis....2

V. Fmoc Solid-Phase Peptide Synthesis (Fmoc-SPPS)....3

VI. General procedure 1: Reaction of arginine containing peptides with probes....4

Figure S1: Optimization and NMR characterization of trapped arginine aldehyde....4

Figure. S2. NMR characterization of aldehyde product....6

Figure S3. Arginine modification of peptide with water-soluble 9,10-phenanthroquinone-3-carboxylic acid **2b**....8

Figure S4. Synthesis of 2,5-dinitrophenanthrene-9,10-dione, **2c** and 2,5-diaminophenanthrene-9,10-dione, **2e**....18

Figure. S5. Screening of electron donating and electron withdrawing analogs of the 9,10-phenanthroquinone probes (**2a-2f**)....21

Figure. S6. Carbonylation reaction generated fluorophore analogs....27

Figure. S7. Electrostatic potential of diverse 9,10 phenanthrenequinone analogs....27

Figure. S8. Chemoselectivity studies....28

Figure. S9. Peptide substrate scope....29

Figure S10. Protein optimization studies....31

Figure S11. Arginine carbonylation of proteins and hydroxylamine fluorophore labeling....56

Figure S12. Arginine carbonylation of cell lysate and hydroxylamine fluorophore labeling....58

Figure S13. Cell Lysate Chemoproteomics Analysis of Arginine Carbonylation Reaction on Cell Lysate....59

Figure S14. Sequence Motif analysis Sequence motif of modified arginine sites....61

Figure S15. Gene Ontology (GO) analysis of hyperreactive arginine sites....61

Figure S16. Confocal microscopy imaging of carbonylated T47D cells....62

Figure. S17. Carbonylation mediated installation of post-translational modifications....63

References....69

I. General.
All commercial materials (Sigma-Aldrich, Fluka and Novabiochem) were used without further purification. All solvents were reagent or HPLC (Fisher) grade. All reactions were performed under air in glass vials. Yields refer to chromatographically pure compounds; % conversions were obtained by comparing HPLC peak areas of products and starting materials. HPLC and MS were used to monitor reaction progress, and products were characterized using MS and NMR.

II. Materials.
Fmoc-amino acids, Rink amide resin, N,N′-iisopropylcarbodiimide(DIC), and N,N-diisopropylethylamine (DIPEA) were obtained from CreoSalus (Louisville, Kentucky). Piperidine, trifluoroacetic acid (TFA), and 1,8-diazabicyclo[5.4.0]undec-7-ene (DBU) were obtained from Alfa Aesar (Ward Hill, Massachusetts). N,N-dimethylformamide (DMF), dichloromethane (CH_2Cl_2), methanol (MeOH) and acetonitrile (ACN) were obtained from VWR (100 Matsonford Road Radnor, Pennsylvania). 1-Hydroxybenzotriazole hydrate (HOBt)), 9,10 phenanthrenequinone, and 9,10-Dioxo-9,10-dihydrophenanthrene-3-carboxylic acid were obtained from Sigma. 1,10-phenanthroline-5,6-dione and 3,8-Dibromo-1,10-phenanthroline-5,6-dione were obtained from Combi-Blocks.

III. Purification (HPLC).
The purification of peptides was performed using high performance liquid chromatography (HPLC) on an Agilent 1100 series HPLC equipped with a C-18 reverse phase column with a particle size of 5 µm. All separations involved a mobile phase of 0.1% formic acid in water (solvent A) and 0.1 % formic acid in acetonitrile (solvent B). The HPLC method used a linear gradient of 0-80% solvent B over 40 minutes at ambient temperature with a flow rate of 1 mL/min. The eluent was monitored by absorbance at 220 nm.

IV. Instrumentation and sample analysis.

NMR. ^{1}H and ^{13}C spectra were acquired at 25 °C in DMSO-d_6, $CDCl_3$ using an Agilent DD2 (400 MHz) spectrometer with a 3-mm He triple resonance (HCN) cryoprobe. All ^{1}H NMR chemical shifts (δ) were referenced relative to the residual DMSO-d_6 peak at 2.50 ppm, $CDCl_3$ peak at 7.26 ppm or internal tetramethylsilane (TMS) at 0.00 ppm. ^{13}C NMR chemical shifts were referenced to DMSO-d_6 at 39.52 ppm and $CDCl_3$ at 77.2 ppm. ^{13}C NMR spectra were proton decoupled. NMR spectral data are reported as chemical shift (multiplicity, coupling constants (J), integration). Multiplicity is reported as follows: singlet (s), broad singlet (br s), doublet (d), doublet of doublets (dd), doublet of triplets (td), triplet (t) and multiplet (m). Coupling constant (J) in hertz (Hz).

Analytical HPLC. Analytical HPLC chromatography (HPLC) was performed on an Agilent 1200 series HPLC equipped with a 5 mm C-18 reversed-phase column. The reaction was monitored by analytical reverse phase HPLC using a gradient of water versus acetonitrile. All separations involved mobile phase with 0.1 % formic acid in water (solvent A) and 0.1 % formic acid in acetonitrile (solvent B). Analytical HPLC method used for purification of peptides a linear gradient of 0-80% solvent B over 40 min at room temperature with a flow rate of 1.0 mL min^{-1}. The peptide

reactions were analyzed by HPLC, and MS. Protein reactions were analyzed by MS and LCMS/MS. HPLC was carried out with 0.1% formic acid: water (solvent A): acetonitrile (solvent B) at detection wavelength 220 nm.

LC/MS. High resolution LC-MS conditions for all purified peptides: Analyses were performed on an ultraperformance LC system (ACQUITY, Waters Corp., USA) coupled with a quadrupole time-of-flight mass spectrometer (Q-ToF Premier, Waters) with electrospray ionization (ESI) in positive mode using Mass lynx software (V4.1) or high-performance LC system (Agilent, 1100 series) coupled with triple quadrupole.

LC-MS (Agilent technologies 6460) with electrospray ionization (ESI) in positive mode using Agilent mass hunter (10.0). Unless otherwise mentioned a sample was injected either onto a C4 column (Phenomenex Aeris™ 3.6 µm WIDEPORE C4 200 Å, LC Column 50 x 2.1 mm) with a 400 µL/min flow rate of mobile phase of solution A (90 % H_2O, 10 % acetonitrile and 0.1 % formic acid (FA)) and solution B (95 % acetonitrile, 5 % H_2O, and 0.1 % formic acid) beginning gradient- Time- 0 min 10 % B; 5 min 28 % B; 20 min 38 % B; 22 min 90 % B; C18 column (ACQUITY UPLC BEH 1.7 µm 1x 50 mm) with a 200 µL/min flow rate of mobile phase of solution A (90 % H_2O, 10 % acetonitrile and 0.1 % formic acid) and solution B (90 % acetonitrile, 10 % H_2O, and 0.1 % formic acid) beginning gradient- Time- 1 min 0% B; 1-10 min 100% B for chromatography analysis (or) directly injected with mobile phase 90 % H_2O: 10 % ACN, 0.1% formic acid at 400 µL/min flow rate in ESI positive mode.

HRMS. High resolution MS data were acquired on Thermo Exactive Plus using a heated electrospray source. The solution was infused at a rate of 10-25 µL/min/electrospray using 3.3 KV. The typical settings were Capillary temp 320 ºC. S-lens RF level was between 30-80 with an AGC setting of 1 E6. The maximum injection time was set to 50 ms. Spectra were taken at 140,000 resolutions at m/z 200 using Tune software and analyze with Thermo's Freestyle software.

V. Fmoc Solid-Phase Peptide Synthesis (Fmoc-SPPS).[1]

Peptides were synthesized manually on a 0.25 mmol scale using Rink amide resin. Resin was swollen with CH_2Cl_2 for 1 h at room temperature. Fmoc was deprotected using 20% piperidine in DMF for 5 min to obtain a deprotected peptide-resin. First, Fmoc protected amino acid (1.25 mmol/5 equiv.) was coupled using HObt (1.25 mmol, 5 equiv.) and DIC (1.25 mmol, 5 equiv.) in DMF for 15 min at room temperature. Followed by the reaction of the N-terminus of all peptides with anthracene-9-carboxylic acid (0.63 mmol, 2.5 equiv) using HOBt (1.25 mmol, 5 equiv.) and DIC (1.25 mmol, 5 equiv.) in DMF to the peptide loaded resin for six hours. Peptides were cleaved from the resin using a cocktail of 94:2:2:2, trifluoroacetic acid: anisole: thioanisole: ethanedithiol (EDT) for 2 h. The resin was removed by filtration and the resulting solution was concentrated. The residue was diluted with ACN/water mixture. The resulting solution was purified by HPLC.

VI. General procedure 1: Reaction of arginine containing peptides with probes.

To 1 mg (1 mM) of arginine containing peptides (**1b-1d**) dissolved in 500 µL of DI water, was added K_3PO_4 (0.012 M, 10 equiv.). Followed by the addition of 9,10-phenanthrenequinone analogs (**2a-2f**) (1.3 equiv.) dissolved in DMSO. The overall reaction volume is 1000 µL with 9:1 ratio of H_2O:DMSO. The reaction mixture was stirred for 6 h at 37 °C and subsequently injected into the HPLC for determining the % conversion of arginine peptides to the aldehyde peptide products and the mass confirmed by LC-MS.

Figure S1: Optimization and NMR characterization of trapped arginine aldehyde

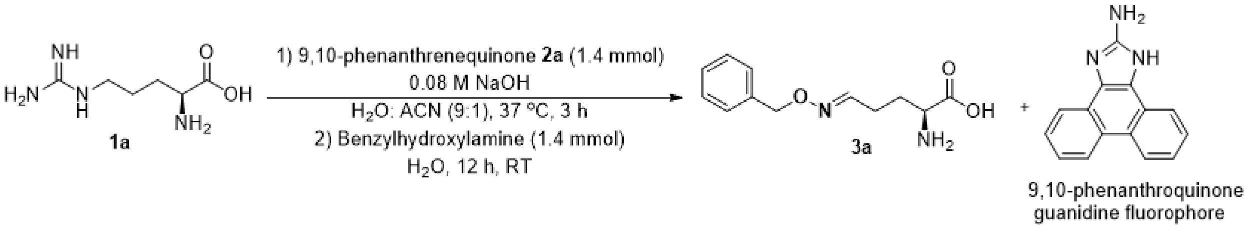

Procedure: 50 mg of L-Arginine **1a** (0.28 mmol) and 9,10-phenanthrenequinone **2a** (5 equiv, 1.44 mmol) was dissolved in 10 mL of 0.08 M NaOH in 9:1 H_2O:ACN. The reaction was stirred for 3 h at 37 °C. Upon completion, excess 9,10 phenanthrenequinone was extracted using DCM. The aqueous layer was concentrated under vacuum and filtered to remove fluorophore by-product. The filtered aqueous layer containing aldehyde was then acidified to pH 3 using 1 M HCl. Benzylhydroxylamine (5 equiv, 1.44 mmol) was added to the solution and the reaction was left to stir for 12 h. The 2-amino-5-((benzyloxy)imino)pentanoic acid oxime product **3a** was purified using HPLC. 15 mg (24% yield) of the desired product **3a** (mixture of *E* and *Z* isomer) was isolated and analyzed by NMR. The ratio of *E:Z* mixture was observed to be 1:0.6.

^{1}H NMR (400 MHz, D_2O): δ 7.49 (t, *J* = 5.7 Hz, 1H), 7.36 – 7.28 (m, 8H), 6.80 (t, *J* = 5.5 Hz, 0 .6H), 5.04 (s, 1.2H), 4.99 (s, 2H), 3.75 (dt, *J* = 14.8, 6.3 Hz, 1.7H), 2.54 – 2.36 (m, 1.4H), 2.32 – 2.22 (m, 1.9H), 1.98 (m, 3.5H).
^{13}C NMR (101 MHz, D_2O): δ 172.8, 152.9, 152.6, 137.1, 136.9, 128.7, 128.4, 128.4, 128.3, 75.5, 75.2, 53.0, 26.7, 26.4, 25.0, 21.5.

^{1}H NMR of compound 3a

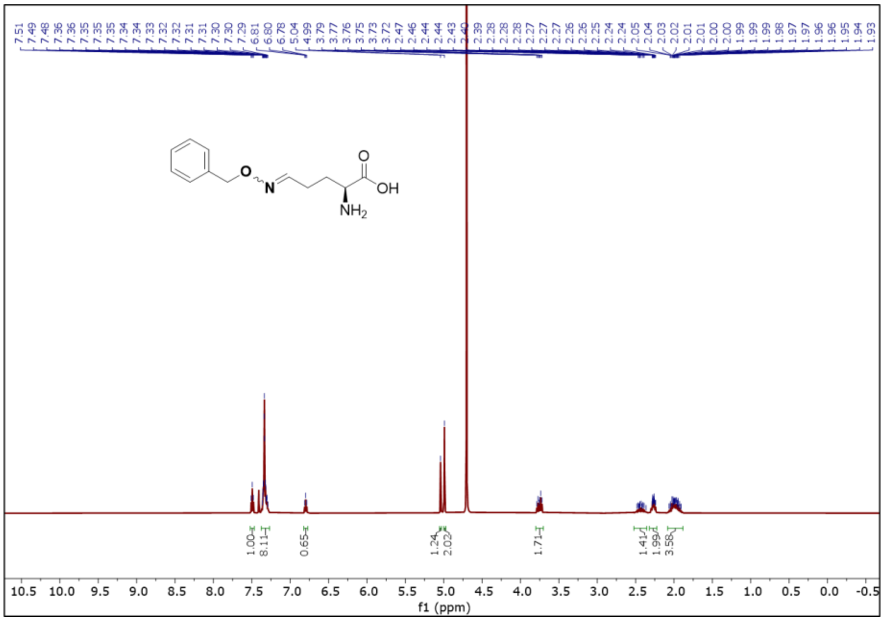

^{13}C NMR of compound 3a

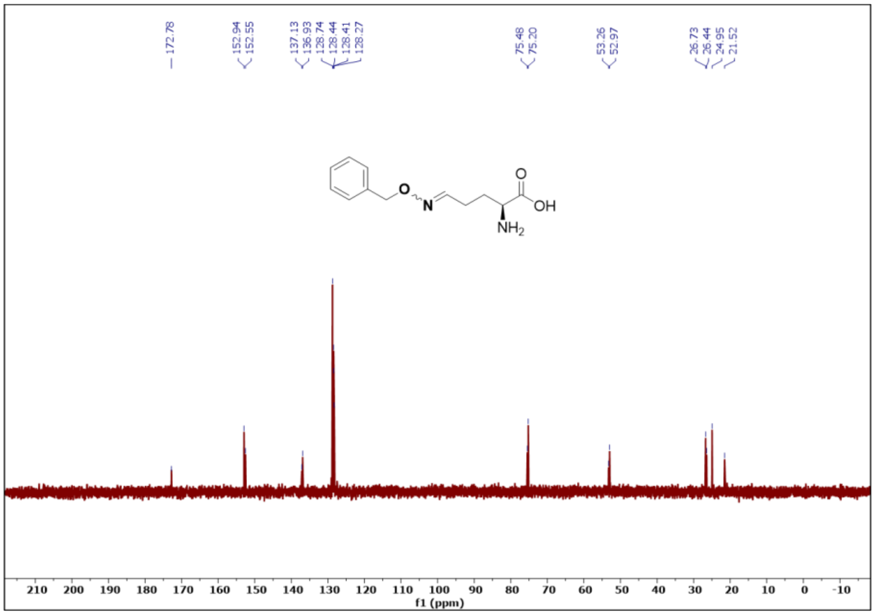

Figure. S2. NMR characterization of aldehyde product.

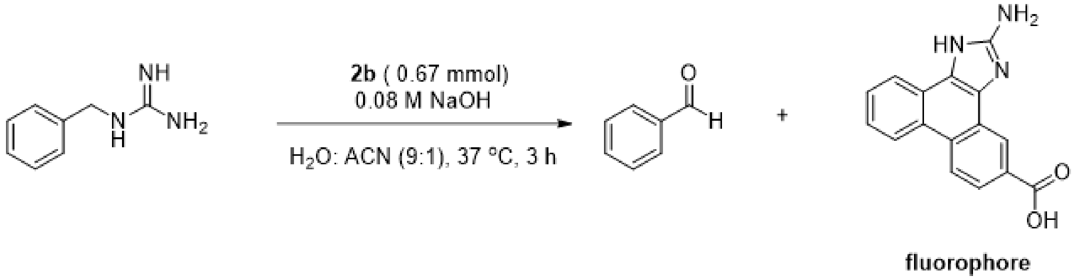

Procedure: Benzyl guanidine (100 mg, 0.67 mmol, 1 equiv.) and 9,10-dioxo-9,10-dihydrophenanthrene-3-carboxylic acid **2b** (152 mg ,0.67 mmol, 1 equiv.) was dissolved in 10 mL of 0.08 M NaOH in 9:1 H_2O:ACN. The reaction was stirred for 3 h at 37 °C. Upon completion, the reaction was then extracted with pentane (2 x 15 mL) and the organic layer was concentrated and dried under vacuum. The resulting benzaldehyde product was a clear liquid (50 mg, 70% yield).

^{1}H NMR of benzaldehyde (400 MHz, Acetone): δ 10.0 (s, 1H), 8.0–7.9 (m, 2H), 7.7–7.7(m, 1H), 7.6 (td, *J* = 7.5, 2.3 Hz, 2H). **^{13}C NMR of benzaldehyde (101 MHz, Acetone)**: δ 154.4, 127.1, 126.7, 126.5, 125.7, 124.3, 123.7, 121.6, 121.2.

^{1}H NMR of fluorophore (400 MHz, DMSO): δ 9.3(s, 1H), 8.8 (d, J = 8.3 Hz, 1H), 8.3 (d, J = 8.2 Hz, 2H), 8.1(dd, J = 8.4, 1.5 Hz, 1H), 7.7 (ddd, J = 8.1, 7.0, 1.1 Hz, 1H), 7.6 (ddd, J = 8.4, 7.0, 1.4 Hz, 1H), 6.3 (s, 2H). **^{13}C NMR of fluorophore (101 MHz, DMSO):** δ 154.5, 126.7, 126.3, 126.1, 125.3, 123.9, 123.2, 121.1, 120.8.

^{1}H NMR of benzaldehyde product

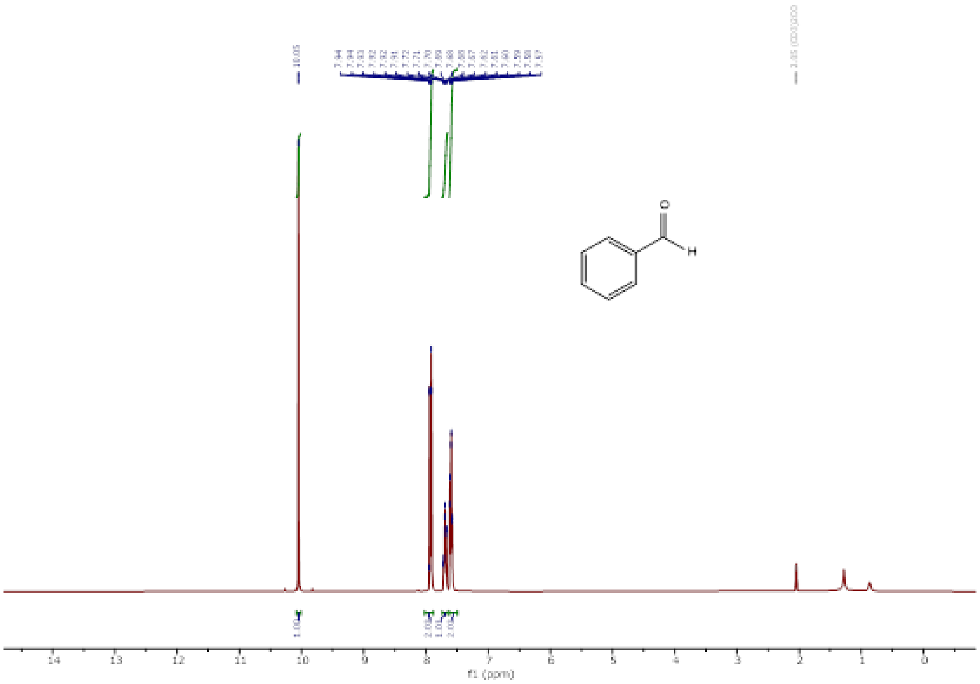

^{13}C NMR of benzaldehyde product

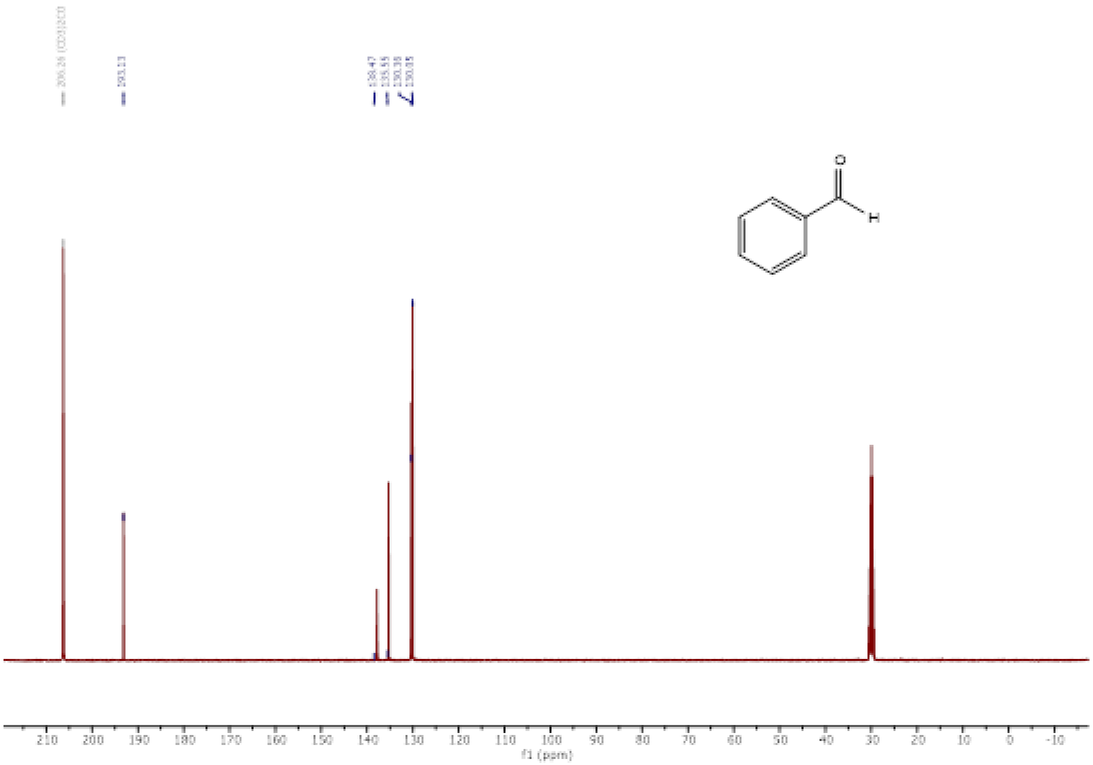

^{1}H NMR of Fluorophore

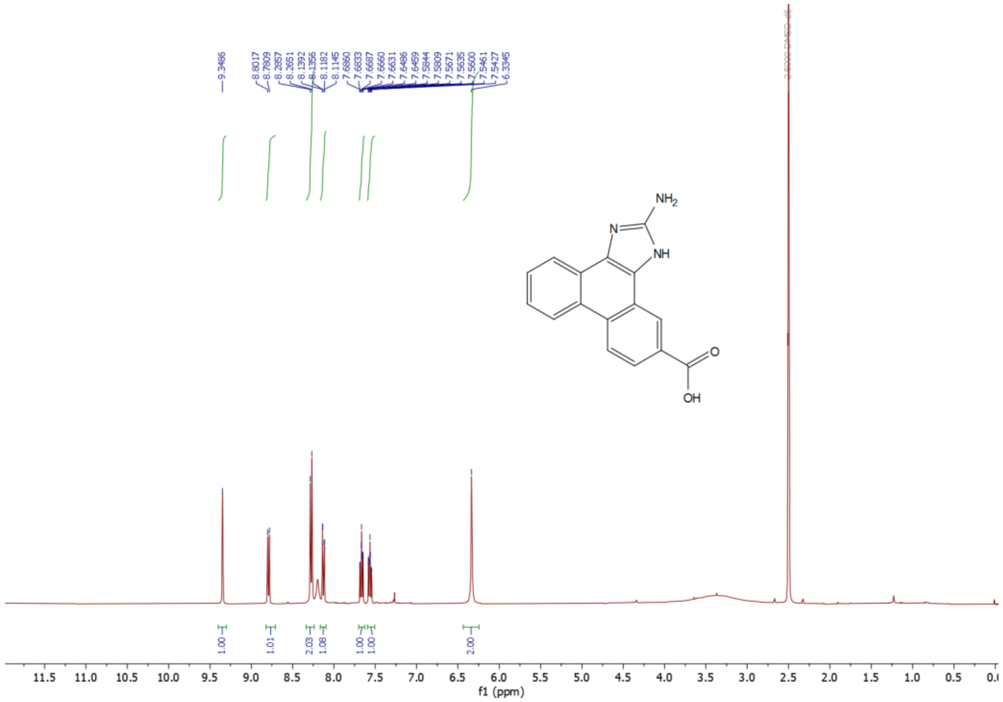

^{13}C NMR of Fluorophore

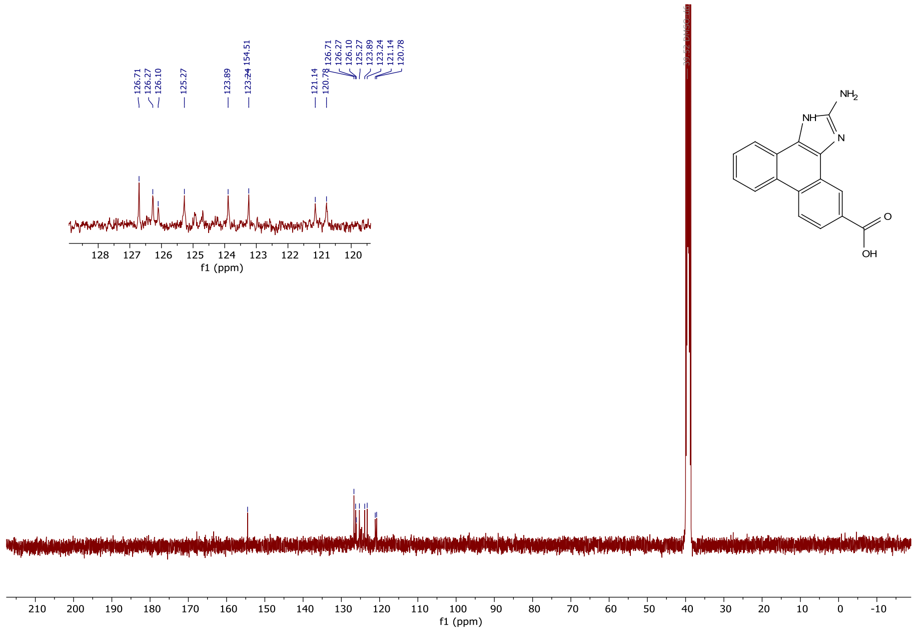

Figure S3. Arginine modification of peptide with water-soluble 9,10-phenanthroquinone-3-carboxylic acid **2b**

Optimization of peptide reaction:

Procedure for the reaction of peptide 1b with probe 2b: To 1 mg (1 mM) of arginine containing peptide **1b** dissolved in 500 µL of DI water, was added different concentrations of bases (table S1). 1.3 equiv. of 9,10-phenanthrenequinone analog **2b** dissolved in DMSO was added to the reaction mixture. The overall reaction volume was 1000 µL with a 9:1 ratio of H_2O:DMSO. The reaction mixture was stirred for 3 h at 37 °C and subsequently injected into the HPLC for determining the % conversion of arginine peptide **1b** to the different peptide products **3b**, **3b'** and **4a,** along with fluorophore byproduct. Reaction was analyzed by LC-MS.

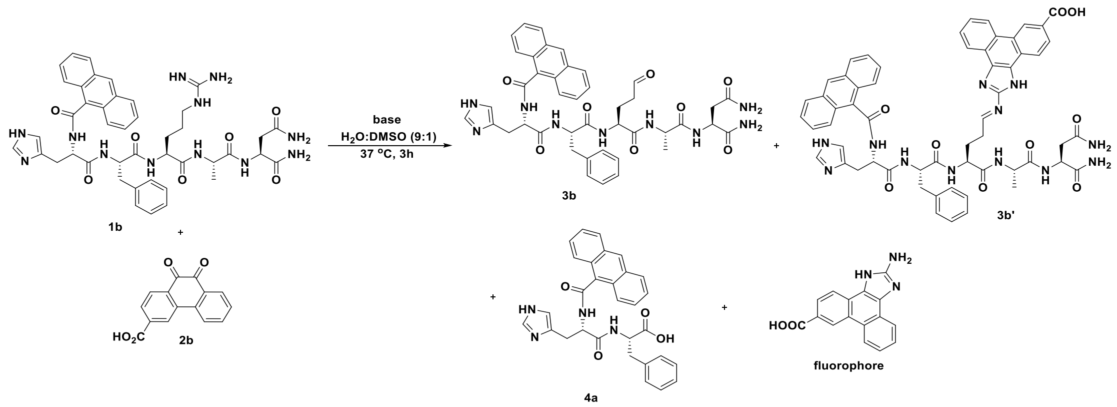

Table S1: Optimization table for reaction of peptide 1b with 9,10-Dioxo-9,10-dihydrophenanthrene-3-carboxylic acid 2b.

Entry	Base	Conc. (M)	Peptide 1b	Peptide 3b	Peptide 3b’	Peptide 4a
1	NaOH	0.004 M	75	18	21	5
2	NaOH	0.008 M	33	13	36	10
3	NaOH	0.012 M	36	25	28	11
4	NaOH	0.02M	22	17	44	15
5	NaOH	0.04 M	37	11	32	18
6	NaOH	0.08 M	19	33	18	29
7	K_3PO_4	0.012 M	13	46	25	16
8	Cs_2CO_3	0.012 M	51	12	26	9
9	$NaHCO_3$	0.012 M	nr	nr	nr	nr
10	NaOAc	0.012 M	nr	nr	nr	nr

Peptide 1a: LCMS: m/z 847.3978 (calcd $[M+H]^+$ = 847.4004), m/z 424.2026 (calcd $[M+H]^+$ = 424.1962), (HPLC analysis at 220 nm). Retention time in HPLC: 13.5.

HPLC trace of peptide 1b

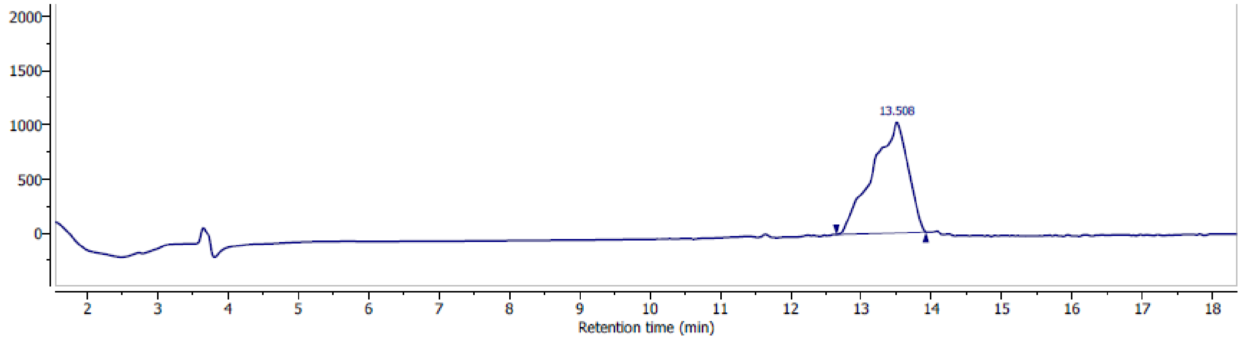

MS-trace of peptide 1b

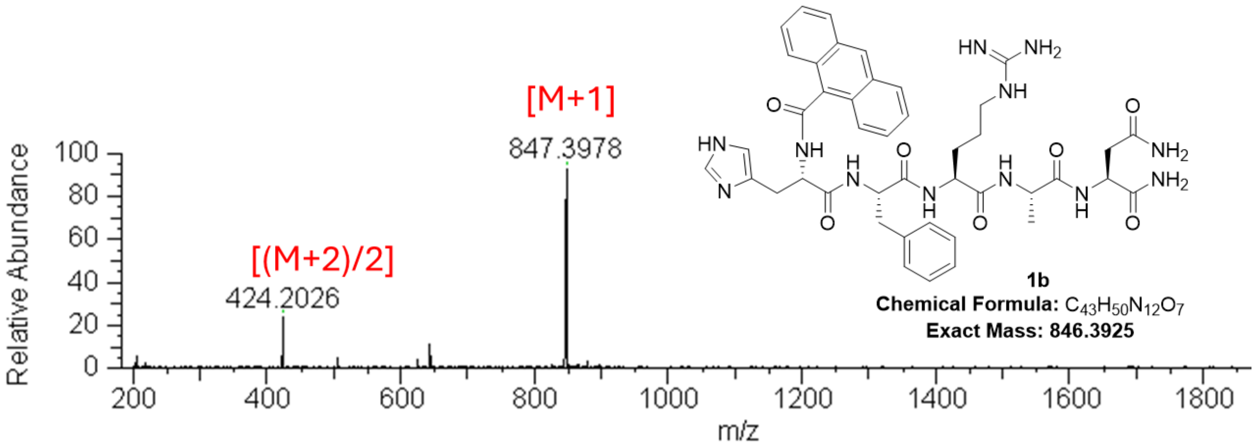

HPLC trace of 9,10-Dioxo-9,10-dihydrophenanthrene-3-carboxylic acid 2b

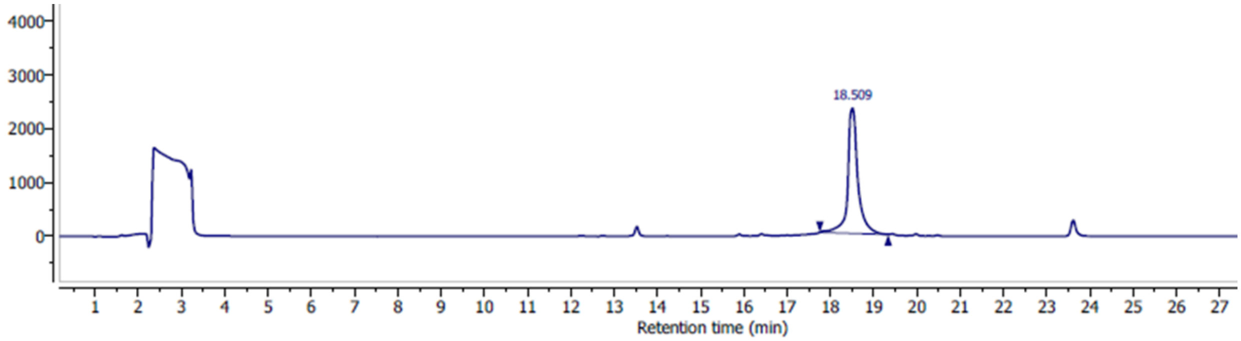

Reaction condition entry 1

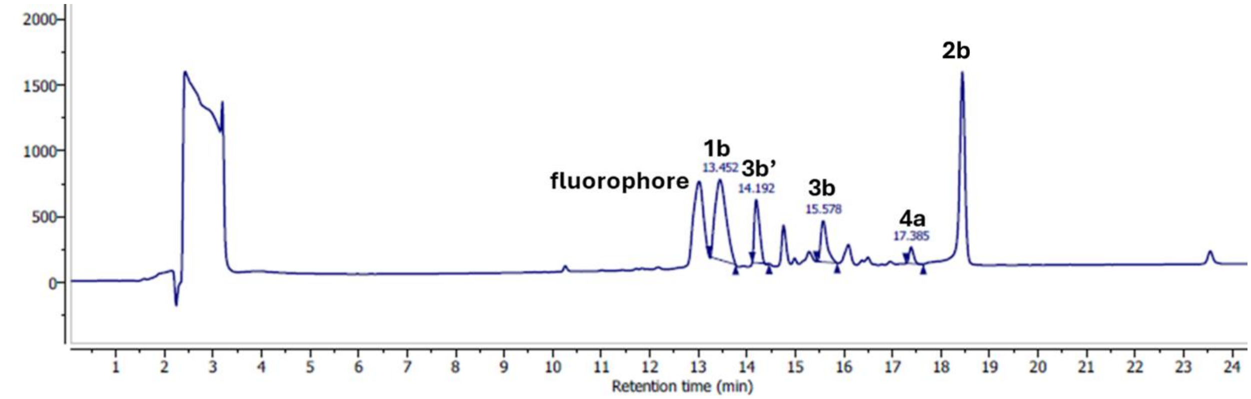

Reaction condition entry 2

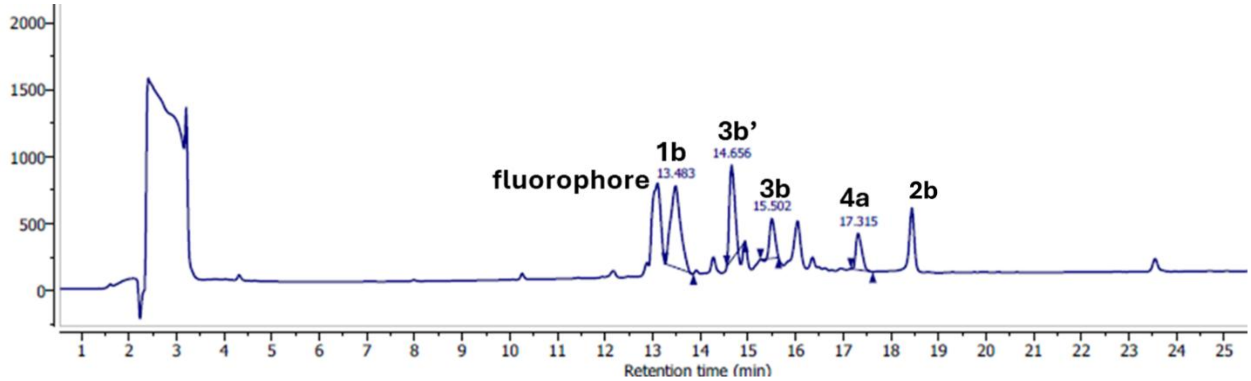

Reaction condition entry 3

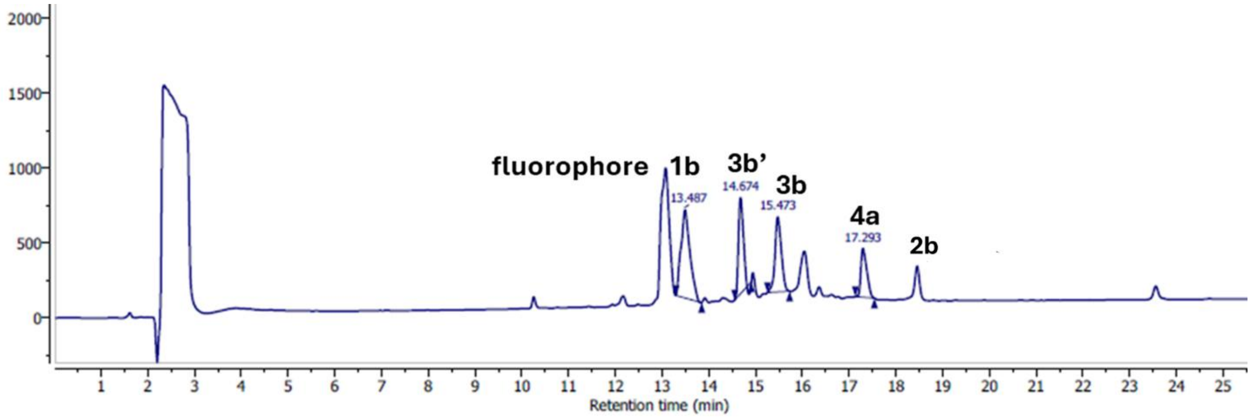

Reaction condition entry 4

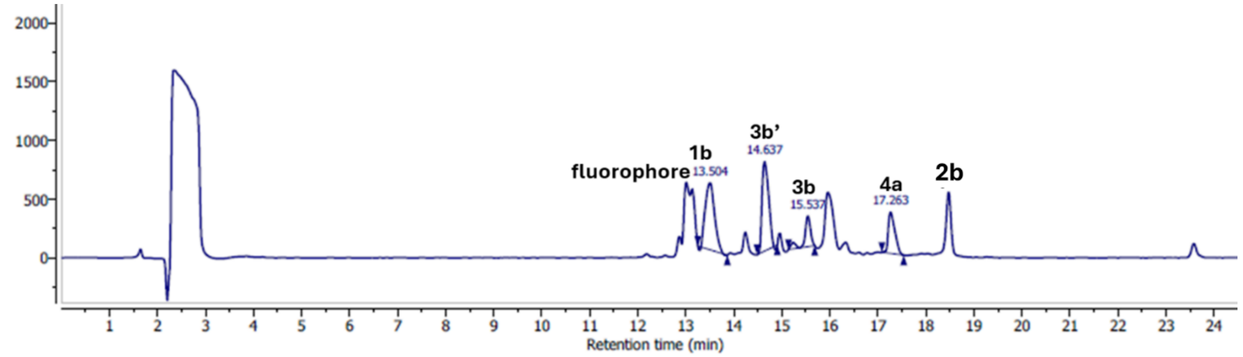

Reaction condition entry 5

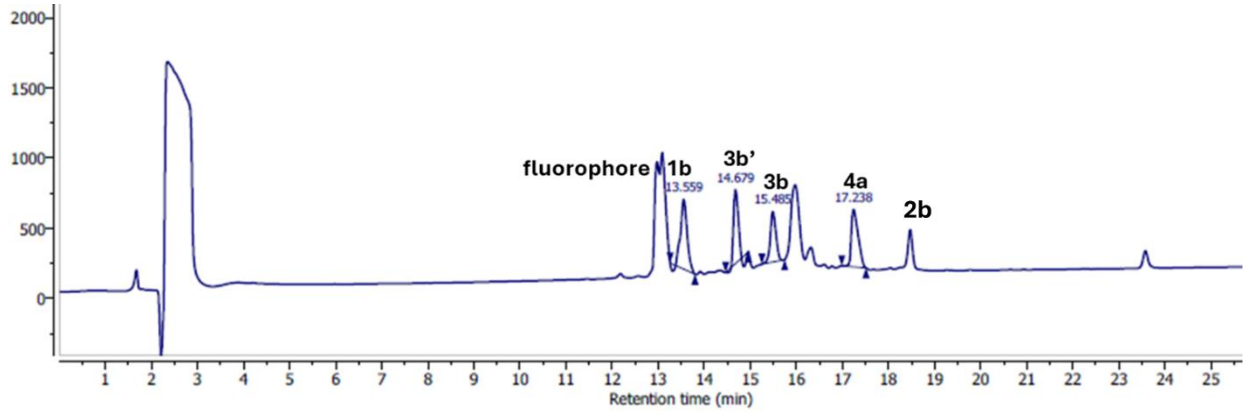

Reaction condition entry 6

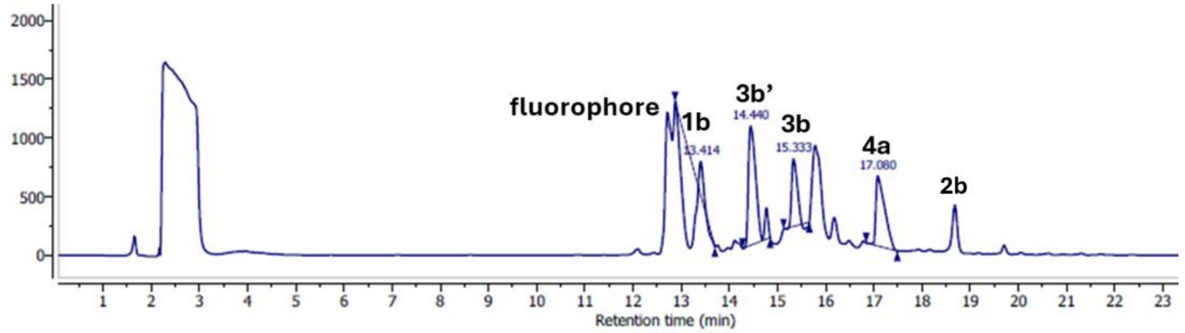

Reaction condition entry 7

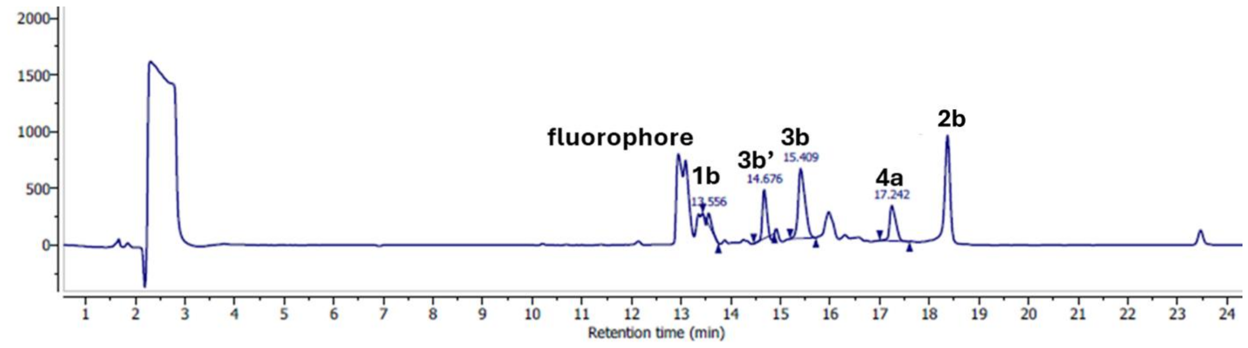

Reaction condition entry 8

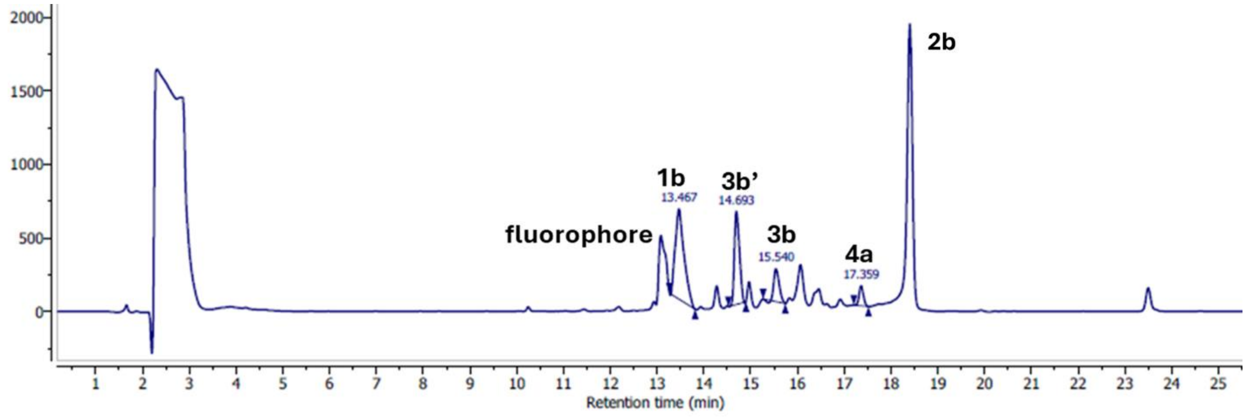

Reaction condition entry 9

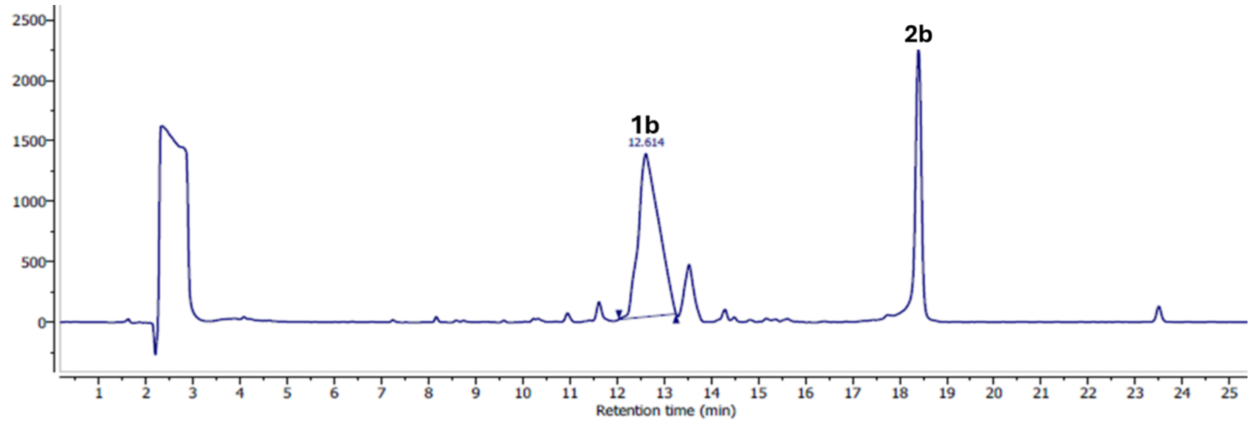

Reaction condition entry 10

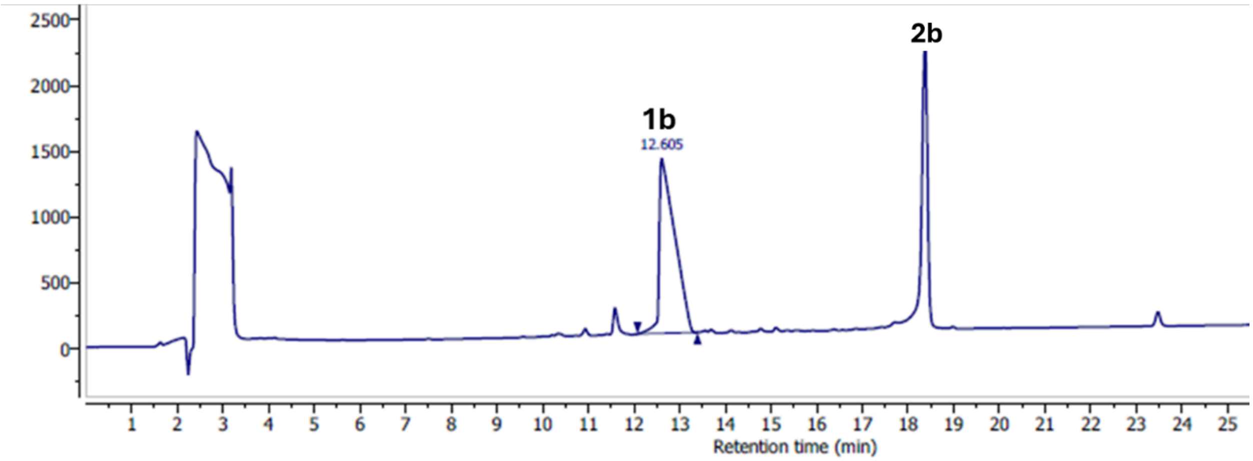

LCMS of Peptide 3b: LCMS: m/z 804.3466 (calcd $[M+H]^+$ = 804.3469), m/z 826.3277 (calcd $[M+Na]^+$ = 826.3289), (HPLC analysis at 220 nm). Retention time in HPLC: 15.4.

MS-trace of peptide 3b

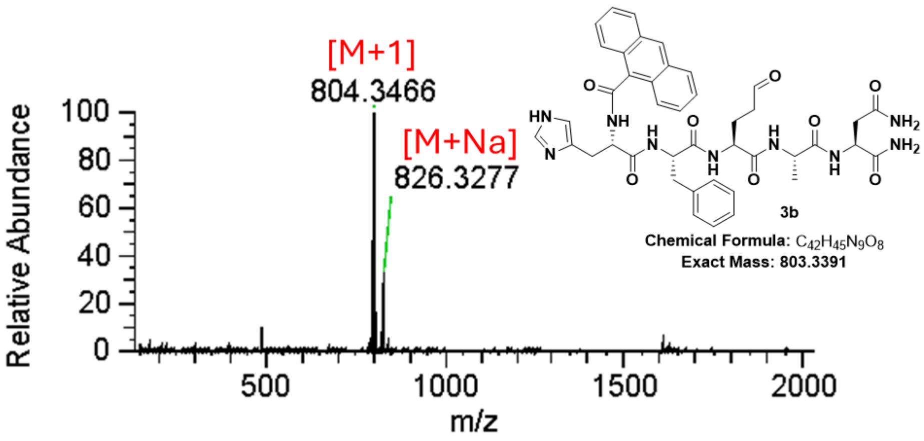

LCMS of Peptide 3b': LCMS: m/z 1063.4199 (calcd $[M+H]^+$ = 1063.4215), m/z 532.2136 (calcd $[M+2]/2^+$ = 532.7068), (HPLC analysis at 220 nm). Retention time in HPLC: 14.6.

MS-trace of peptide 3b'

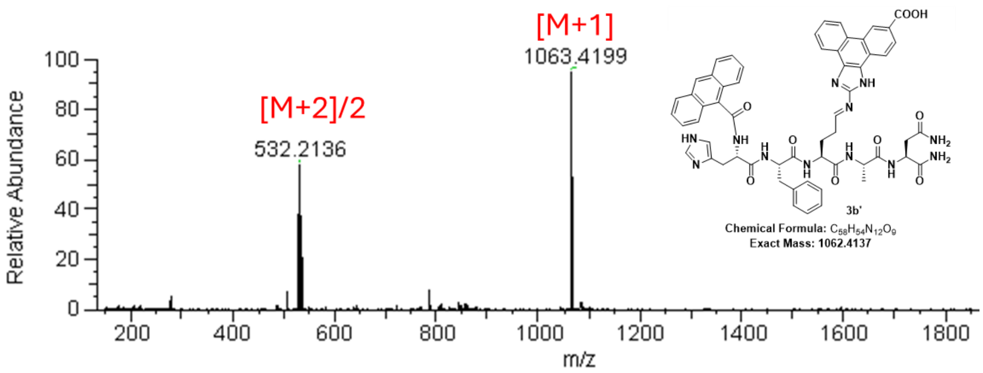

LCMS of Peptide 4b: LCMS: m/z 507.2028 (calcd $[M+H]^+$ = 507.2032), (HPLC analysis at 220 nm). Retention time in HPLC: 17.2.

MS-trace of peptide 4b

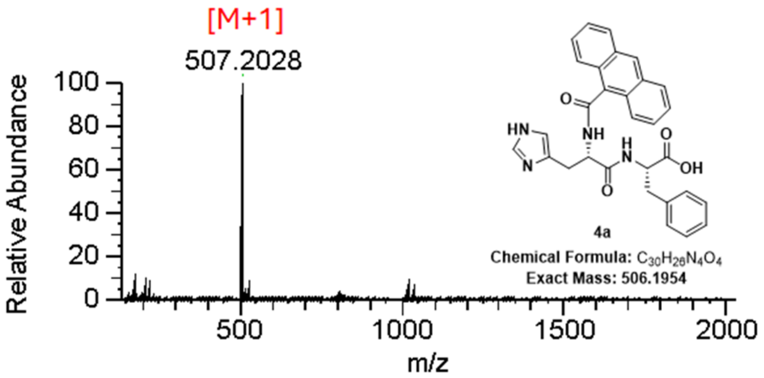

LCMS of fluorophore: LCMS: m/z 277.0851 (calcd $[M+H]^+$ = 277.0930), (HPLC analysis at 220 nm). Retention time in HPLC: 13.0.

MS-trace of fluorophore

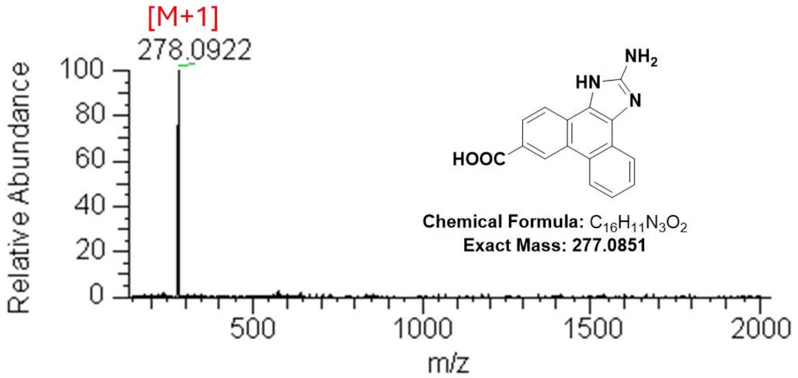

Proposed mechanism for cleavage product 4a:

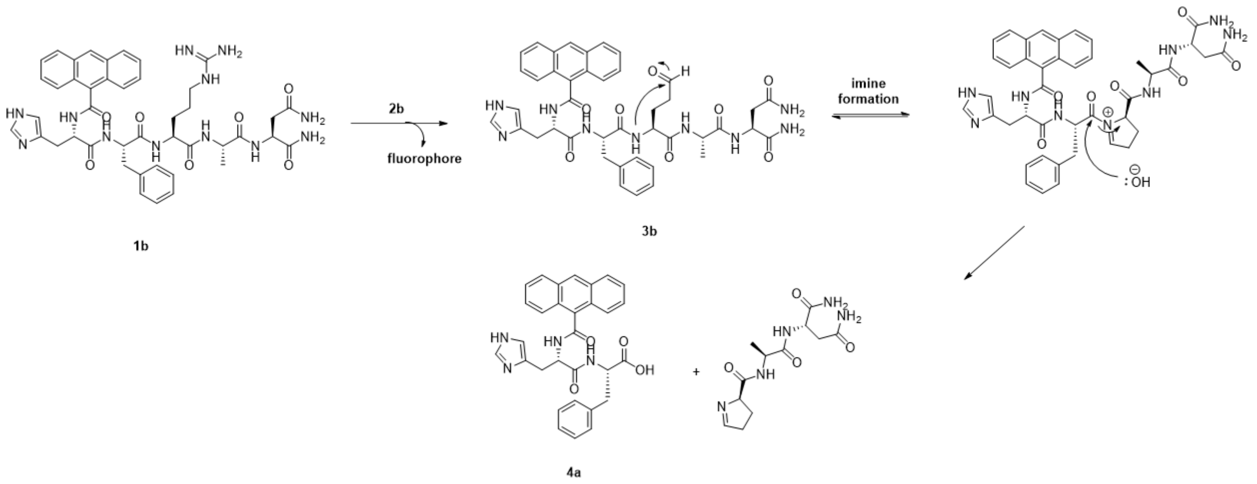

Cleavage of C-Terminal Arg:

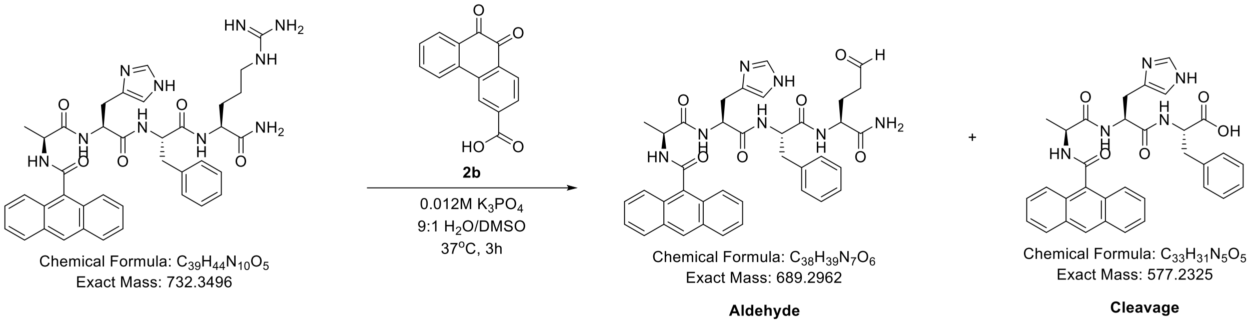

Procedure: To 1 mg (1 mM) of arginine containing peptide dissolved in 500 µL of DI water, was added K_3PO_4 (0.012 M).**2b** (1.3 equiv) dissolved in DMSO was added to the reaction mixture. The overall reaction volume was 1000 µL with a 9:1 ratio of H_2O:DMSO. The reaction mixture was stirred for 3 h at 37 °C and subsequently injected into the HPLC for determining the % conversion of arginine containing peptide Anthracene-AHFR to aldehyde and cleavage product, along with fluorophore byproduct. Reaction was analyzed by LC-MS. Conversion of Anthracene-AHFR to aldehyde was calculated to be 27%. Conversion of Anthracene-AFHR to cleavage product was calculated to be 13%.

HPLC Trace of Reaction

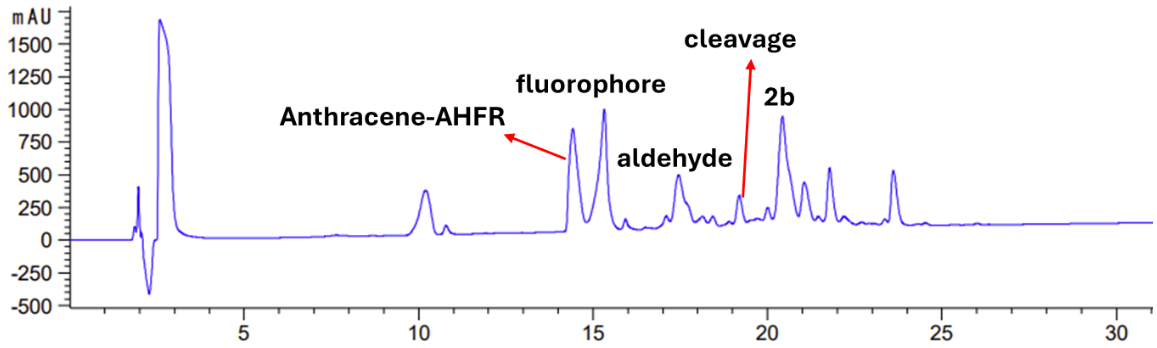

HRMS of Anthracene-AHFR Peptide

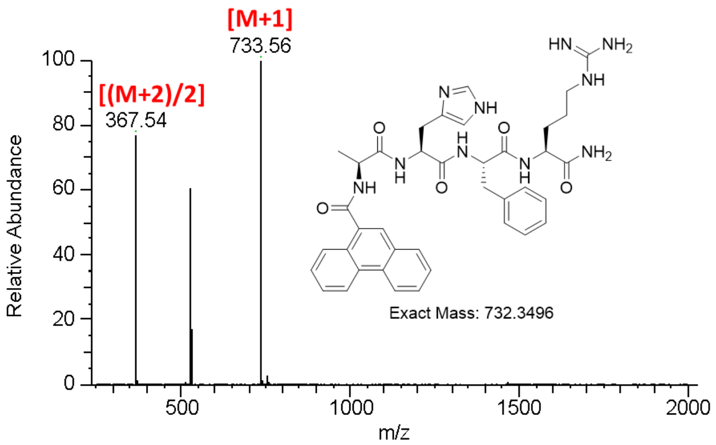

HRMS of Aldehyde Product

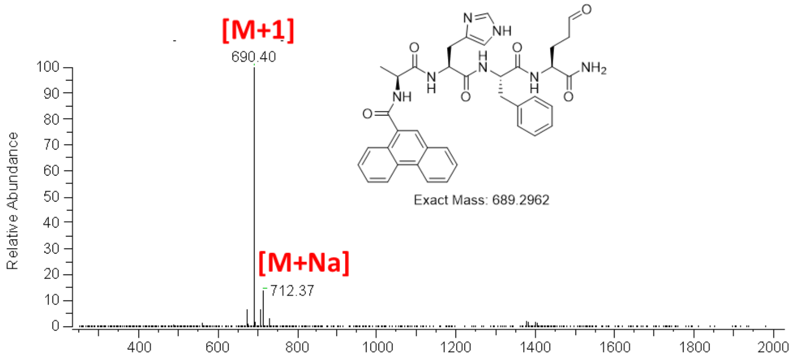

HRMS of Cleavage Product

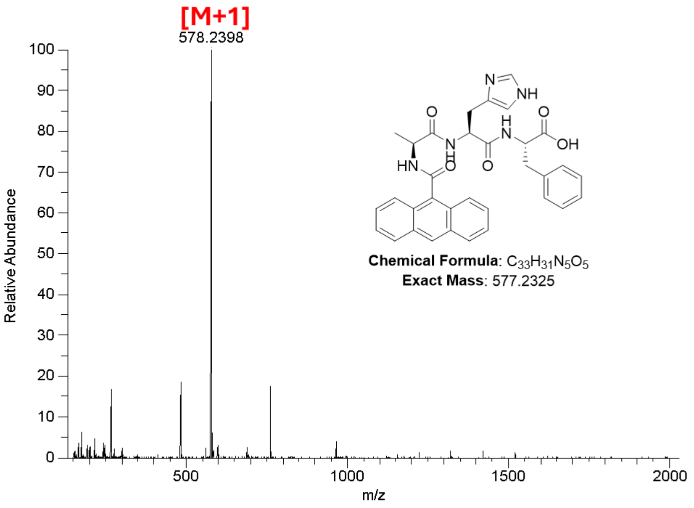

Figure S4. Synthesis of 2,5-dinitrophenanthrene-9,10-dione, **2c** and 2,5-diaminophenanthrene-9,10-dione, **2e**.

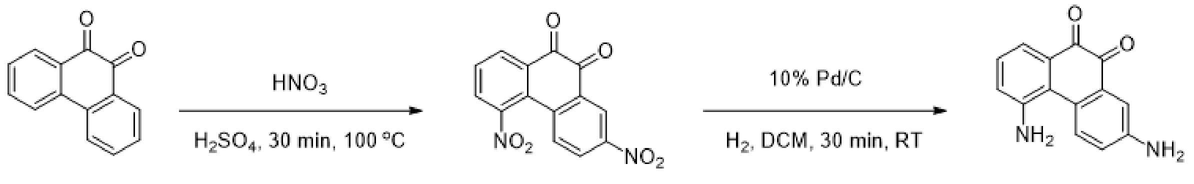

2a 2c 2e

Procedure: 9,10 phenanthrenequinone **2a** (1 g, 4.8 mmol, 1 equiv.) was dissolved in 9 mL of 12 M H_2SO_4 and 10 mL of 70% HNO_3 and stirred at 100 °C for 30 minutes. The reaction was neutralized using 50 mL of DI H_2O and 5 g of NaOH to recrystalize 5-dinitrophenanthrene-9,10-dione **2c,** and further purification done by silica column using a 20% gradient of EtOAc/hexane. To synthesize **2e**, **2c** was reacted with 10% Pd/C in DCM under hydrogen gas for 30 min. Solid Pd/C was filtered from the reaction and **2e** was purified by silica column using 20% gradient of EtOAc/Hexanes.

^{1}H NMR of 2,5-dinitrophenanthrene-9,10-dione (2c): (400 MHz, $CDCl_3$) δ 8.9 (d, *J* = 2.6 Hz, 1H), 8.5 (dd, *J* = 8.9, 2.6 Hz, 1H), 8.4 (dd, *J* = 7.8, 1.5 Hz, 1H), 8.0 (dd, *J* = 7.9, 1.5 Hz, 1H), 7.8 (t, *J* = 7.9 Hz, 1H), 7.7 (d, *J* = 8.9 Hz, 1H).

^{13}C NMR of 2,5-dinitrophenanthrene-9,10-dione (2c): (101 MHz, $CDCl_3$) δ 178.8, 178.7, 137.1, 133.7, 132.5, 132.1, 131.6, 130.1, 129.4, 127.8, 125.7.

^{1}H NMR of 2,5-diaminophenanthrene-9,10-dione (2e): (400 MHz, DMSO) δ 8.2 (d, *J* = 8.7 Hz, 1H), 7.2 (dd, *J* = 7.3, 1.5259 Hz, 1H), 7.1 (d, *J* = 2.8 Hz, 1H), 7.1 (dd, *J* = 8.0, 1.5 Hz, 1H), 7.1 – 7.0 (m, 1H), 6.9 (dd, *J* = 8.7, 2.8 Hz, 1H), 5.7 (s, 2H), 5.5(s, 2H).

^{13}C NMR of 2,5-diaminophenanthrene-9,10-dione (2e): (101 MHz, DMSO) δ 182.1, 147.7, 145.7, 130.9 130.0, 127.8, 126.8, 125.1, 124.3, 121.2, 120.0, 118.9, 112.9.

^{1}H NMR of 2,5-dinitrophenanthrene-9,10-dione (2c)

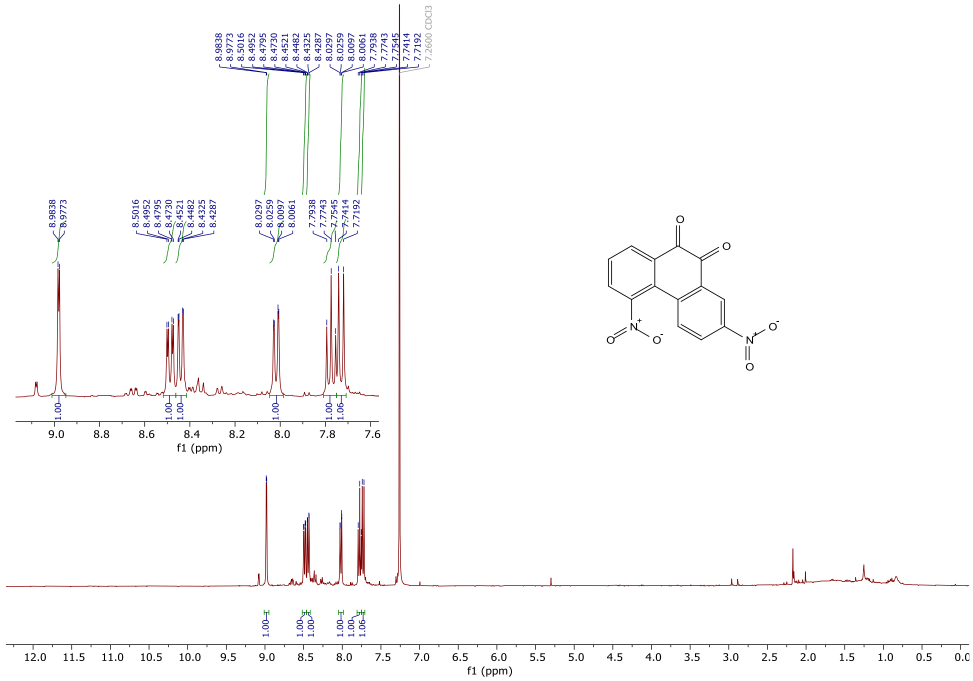

^{13}C NMR of 2,5-dinitrophenanthrene-9,10-dione (2c)

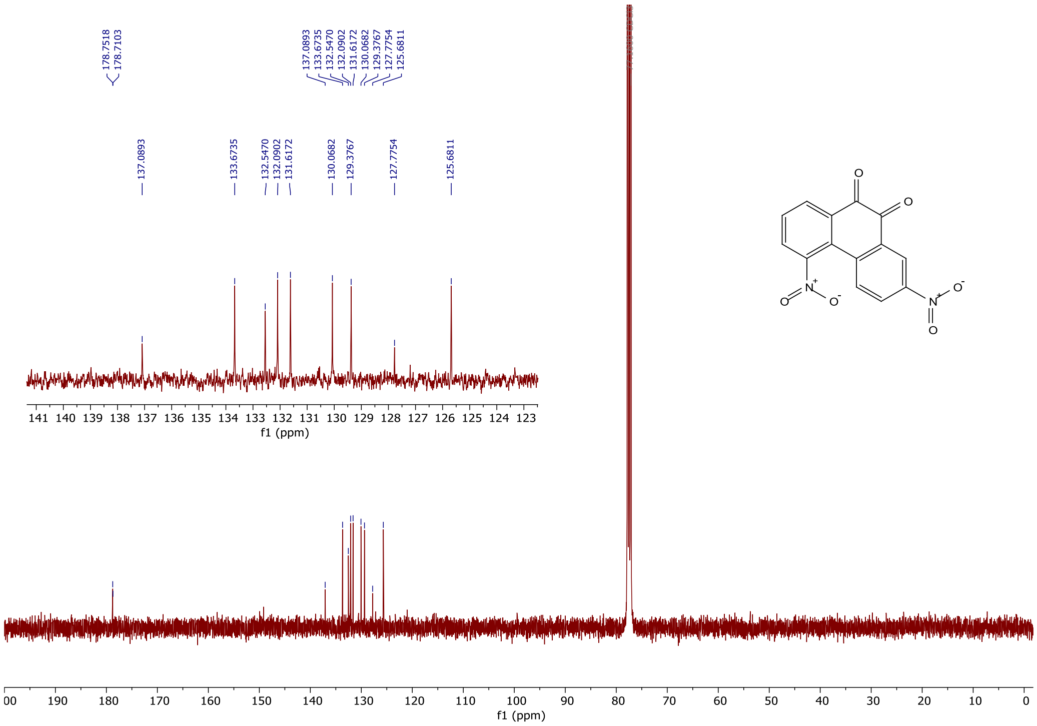

^{1}H NMR of 2,5-diaminophenanthrene-9,10-dione (2e)

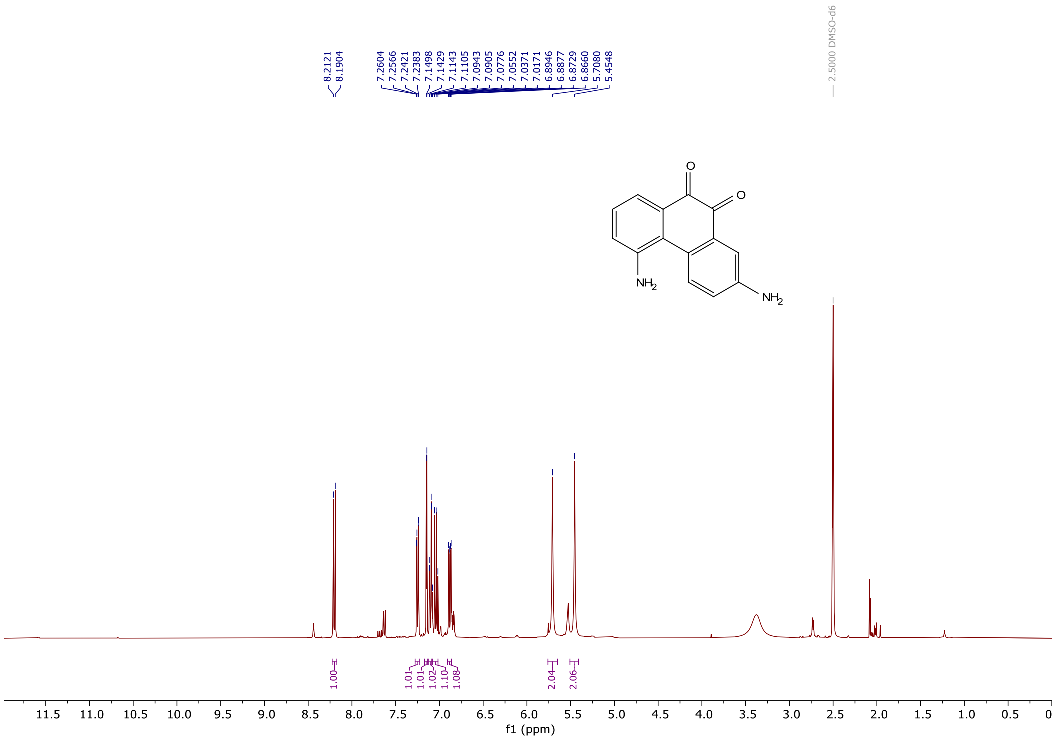

^{13}C NMR of 2,5-diaminophenanthrene-9,10-dione (2e)

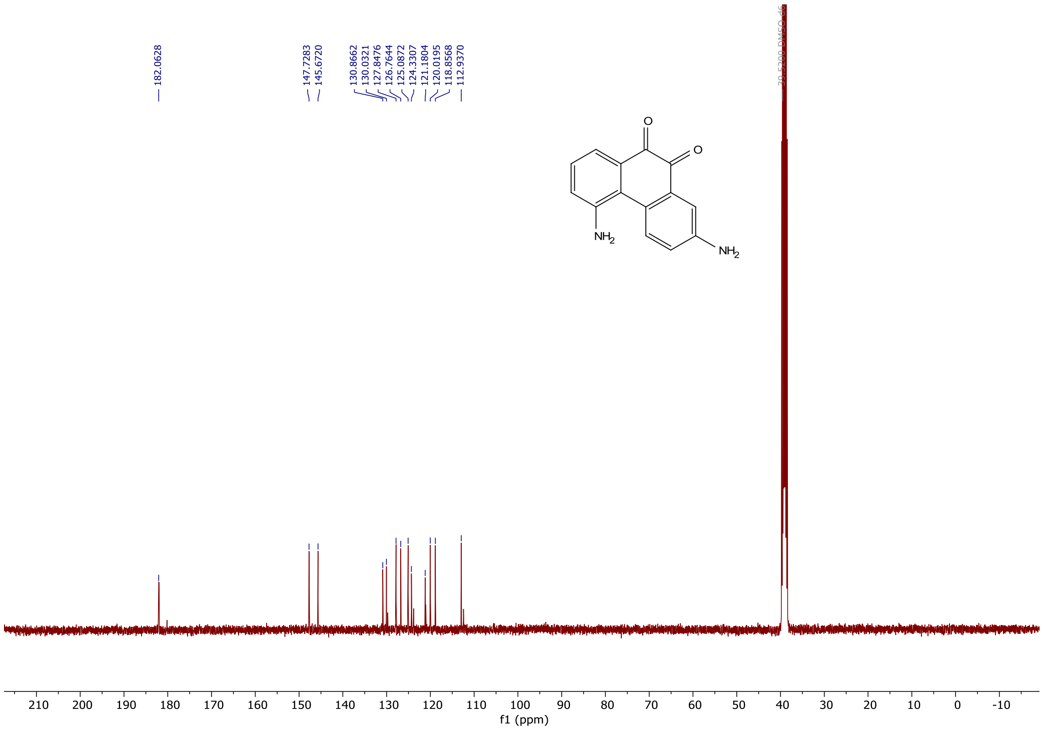

Figure. S5. Screening of electron donating and electron withdrawing analogs of the 9,10-phenanthroquinone probes (**2a-2f**).

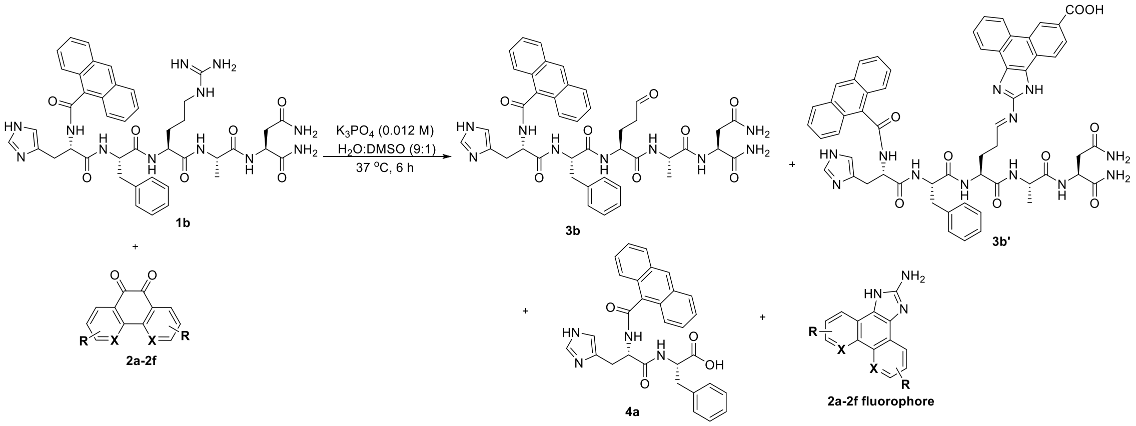

Procedure for the reaction of peptide 1b with different probes (2a-2f): To 1 mg (1 mM) of arginine containing peptide **1b** dissolved in 500 µL of DI water, was added K_3PO_4 (0.012 M). 1.3 equiv. of various probes **2a-2f** (1.3 equiv) dissolved in DMSO was added to the reaction mixture. The overall reaction volume was 1000 µL with a 9:1 ratio of H_2O:DMSO. The reaction mixture was stirred for 6 h at 37 °C and subsequently injected into the HPLC for determining the % conversion of arginine peptide **1b** to the different peptide products **3b**, **3b'** and **4a,** along with fluorophore byproduct. Reaction was analyzed by LC-MS.

Procedure for probe degradation: To determine the amount of probe degradation under the reaction conditions, 3 mg of probes (**2a-2f**) were dissolved in 100 µL of DMSO followed by addition of K_3PO_4 (0.012 M). DI water was added to get the total reaction volume of 1000 µL with a 9:1 ratio of H_2O:DMSO. The reaction mixture was stirred for 6 h at 37 °C and subsequently injected into the HPLC to determine the degradation of the probes (**2a-2f**) in the reaction.

Table S2. Reactivity screening of probes 2c-2f:

Entry	Probe structure	% conversion of peptide 3b
**2a**	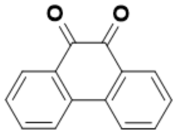	nr*
**2b**	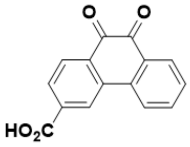	46%

**2c**	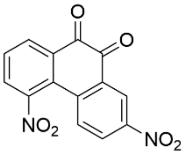	50[a]
**2d**	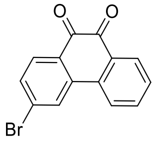	~6%
**2e**	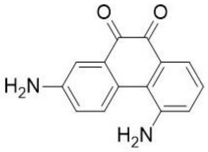	nr
**2f**	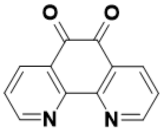	52%<br>71% (10 h)

nr *= no reaction,* ***nr**** *= no reaction under optimized condition but reaction condition was modified to show the generation of the fluoro-phore byproduct,* ***nd*** *= not determined as reaction decomposed rapidly* [a] *reaction was evaluated using 0.01 M NaOH as the base as reaction decomposed in K3PO4 due to high reactivity of probe.*

HPLC Trace of Probes (2a-2f), probe degradations, and reaction of probes with peptide 1b

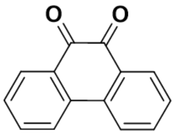

2a

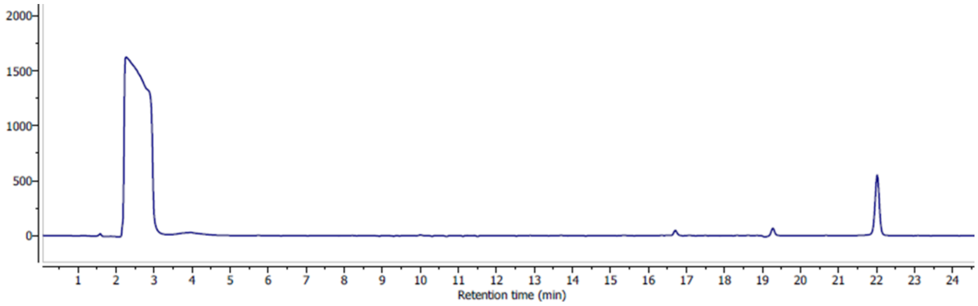

Probe degradation of **2a**

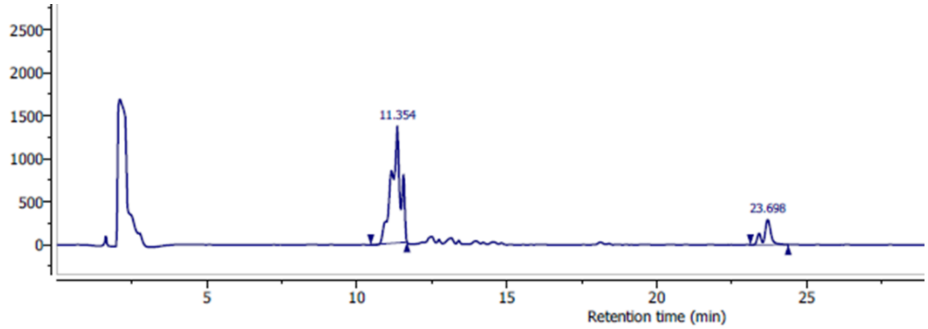

Reaction of **2a** with **1b**

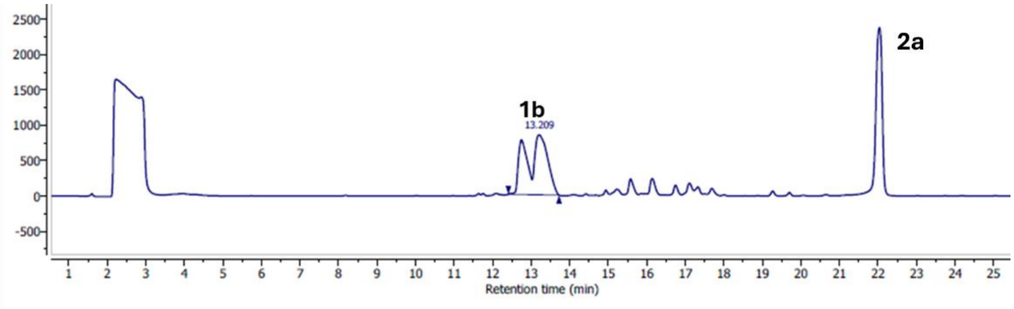

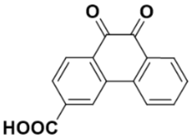

2b

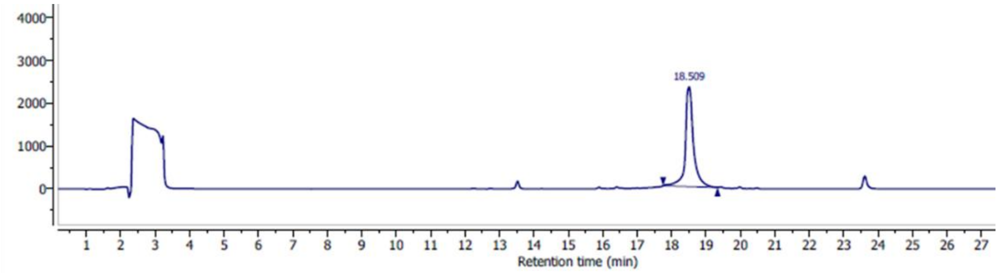

Probe degradation of **2b**

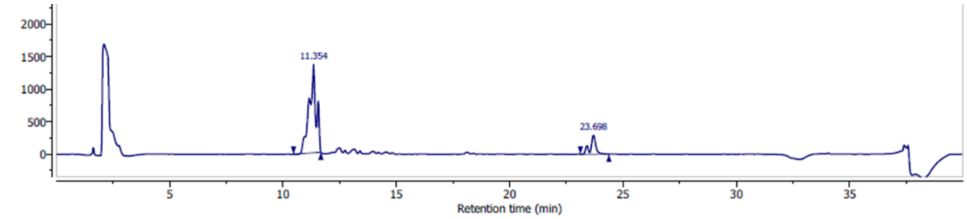

Reaction of **2b** with **1b**

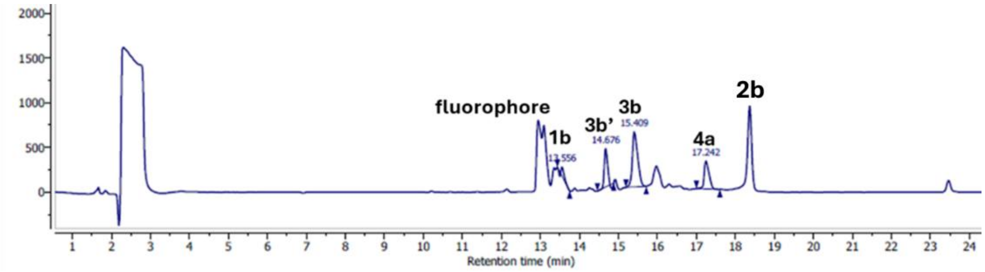

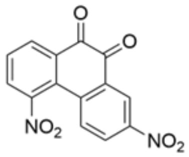

2c

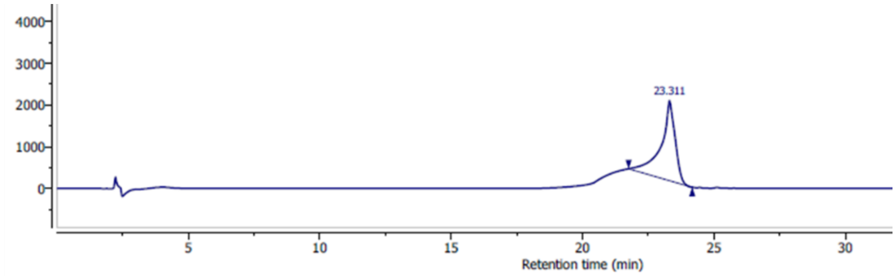

Probe degradation of **2c**

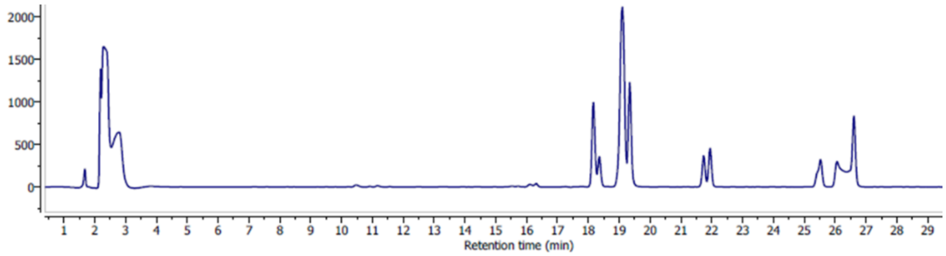

Reaction with **2c** with **1b**

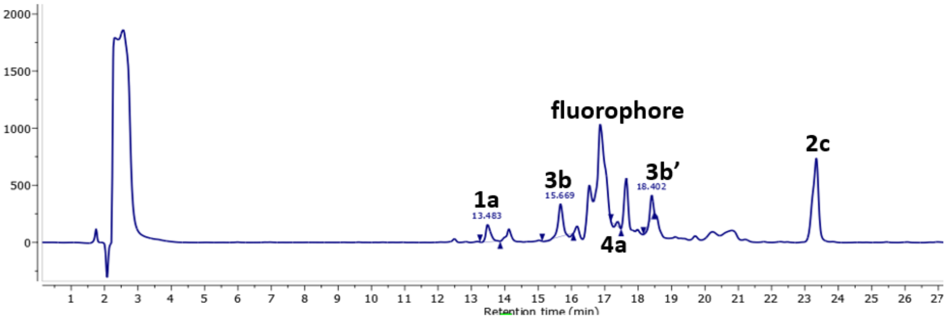

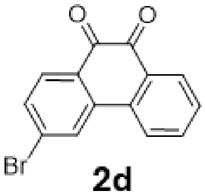

2d

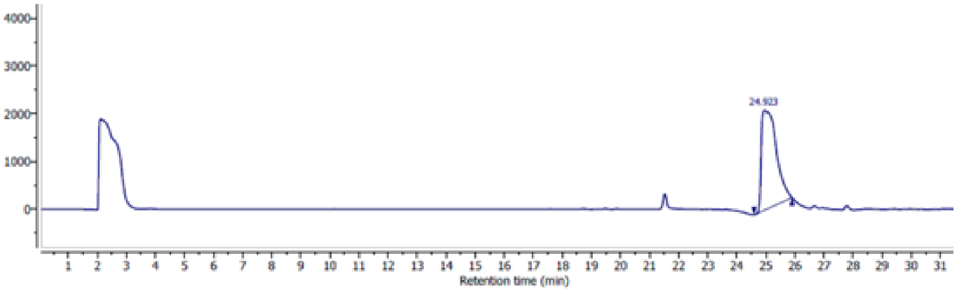

Probe degradation of **2d**

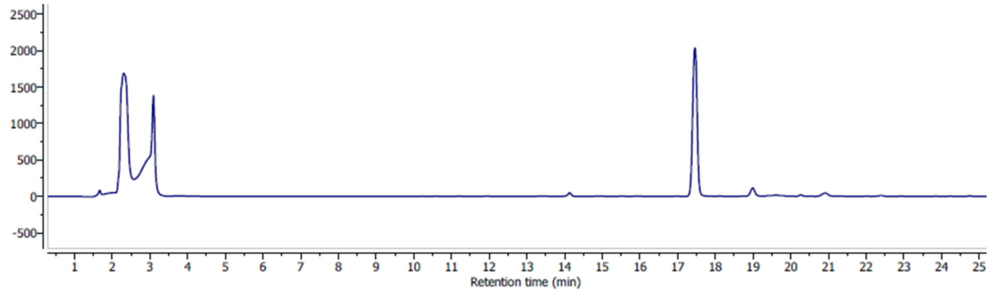

Reaction of **2d** with **1b**

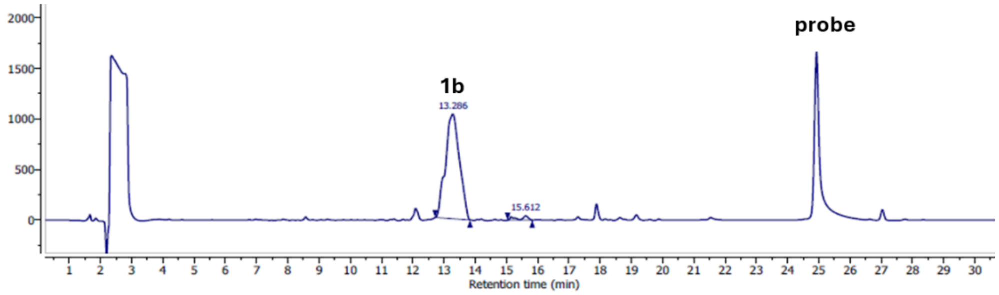

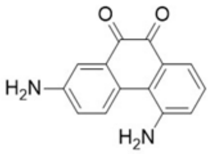

2e

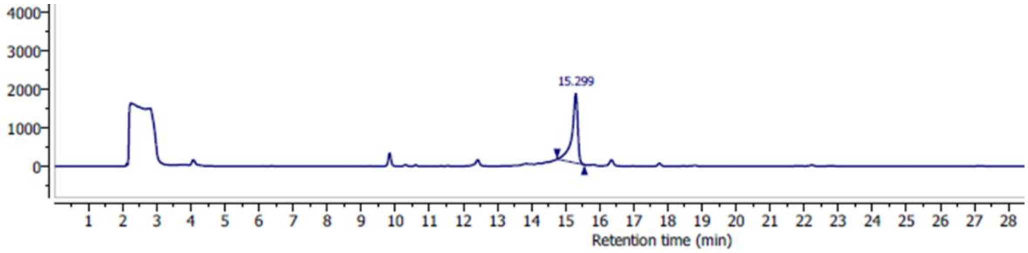

Probe degradation of **2e**

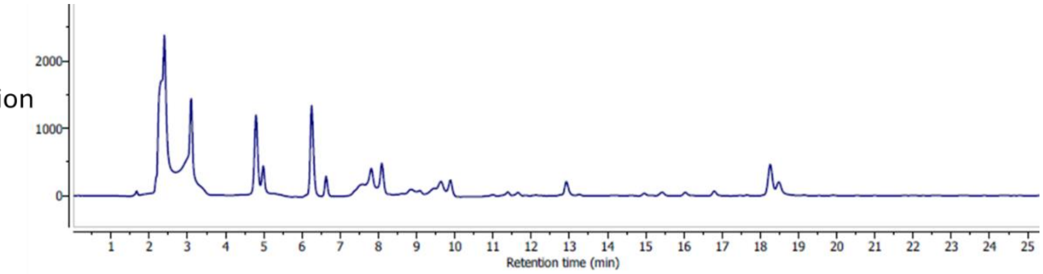

Reaction of **2e** with **1b**

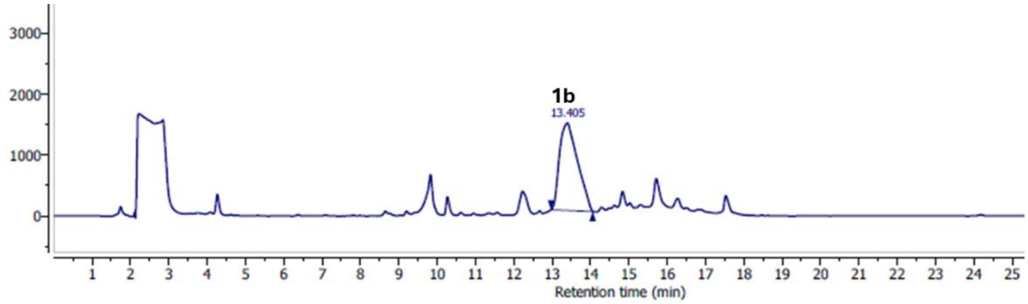

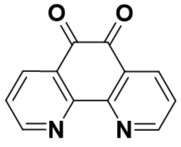

2f

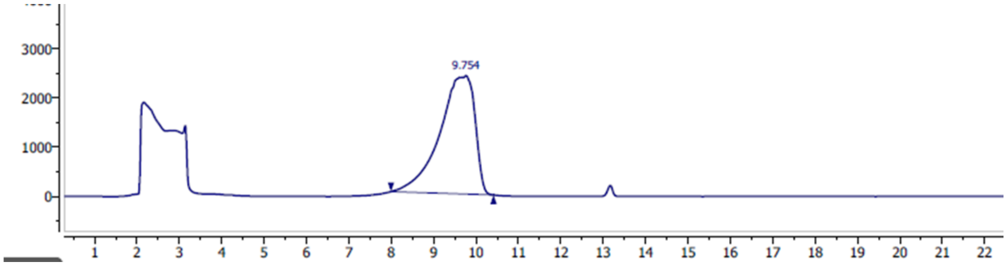

Probe degradation of **2f**

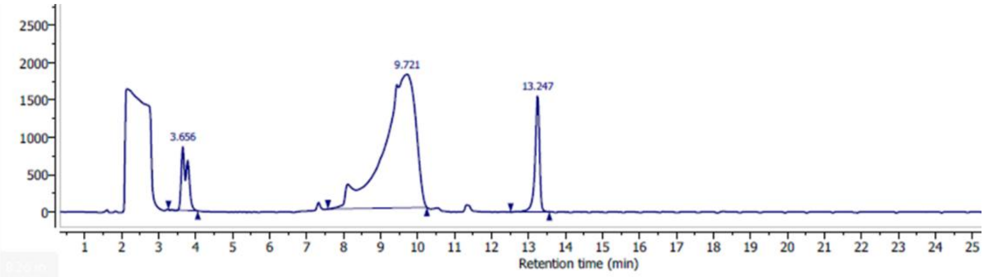

Reaction of **2f** with **1b**

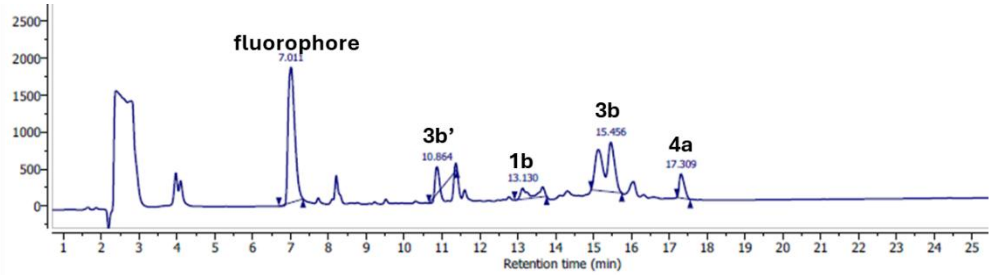

Reaction of **2f** with **1b** (10 h)

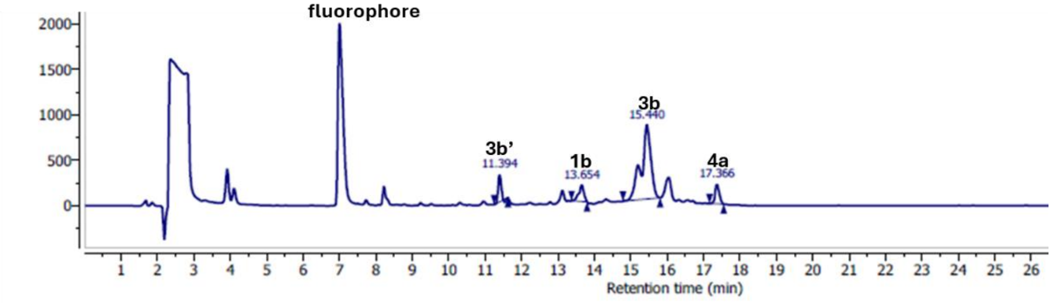

LCMS of 2f-fluorophore: LCMS: m/z 236.0929 (calcd $[M+H]^+$ = 236.0936), (HPLC analysis at 220 m). Retention time in HPLC: 7.2.

MS-trace of 2f-fluorophore

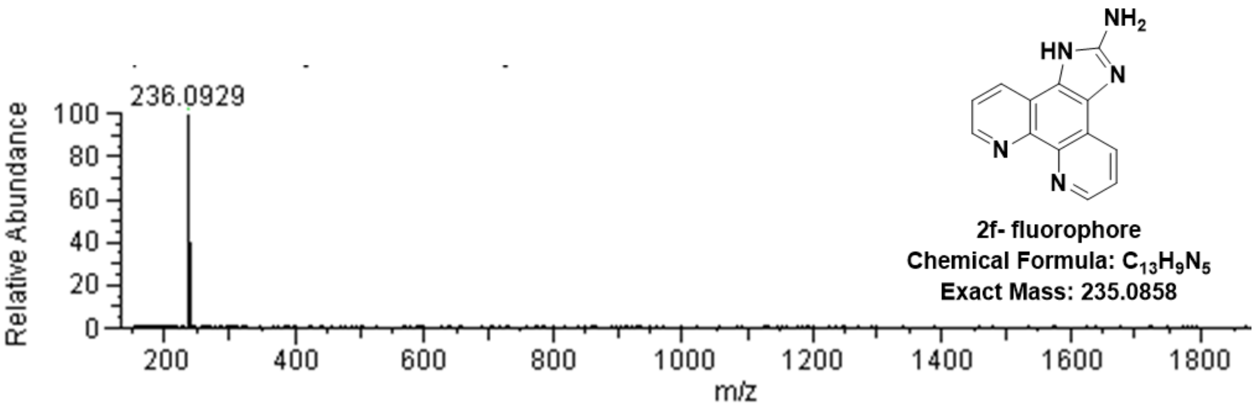

Figure. S6. Carbonylation reaction generated fluorophore analogs

Below is a visual representation of fluorophore formation when guanidine is reacted with 9, 10 phenanthrenequinone analogs (2a-2f). The visual representation of reaction (before and after) is shown below.

Before fluorophore formation:

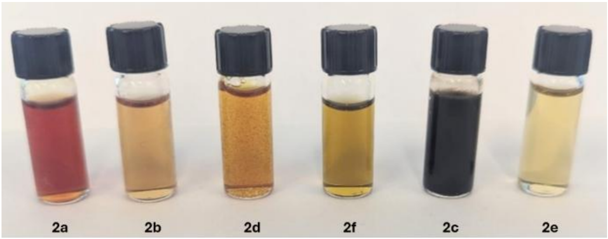

After fluorophore formation:

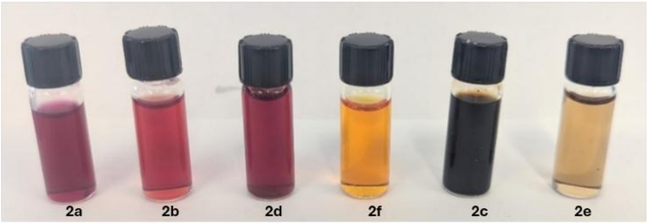

Figure. S7. Electrostatic potential of diverse 9,10 phenanthrenequinone analogs

Optimization and frequency calculation of all structures were evaluated at the (B3LYP-D3BJ) 6-31G+DP level of theory. Solvent effects were also calculated using the polarizable continuum model (PCM). Analysis of the electrostatic potential (ESP) identified an increasing electrophilicity of electron withdrawing analogs of 9,10 phenanthrenequinone (2b, 2c, 2f).

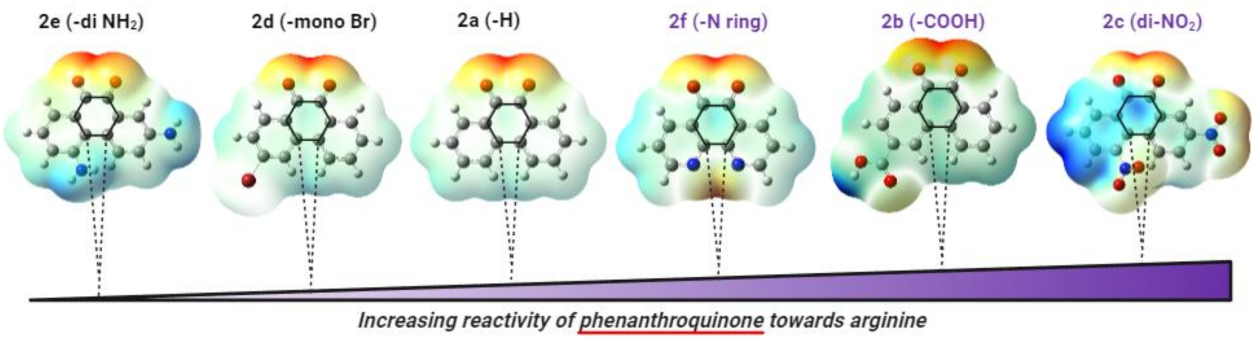

Figure. S8. Chemoselectivity studies.

Reaction of peptide **1c** (anthracene-KNVWCMDS) with 9,10 phenanthrenequinone analog **2b** was carried out using **general procedure 1**.

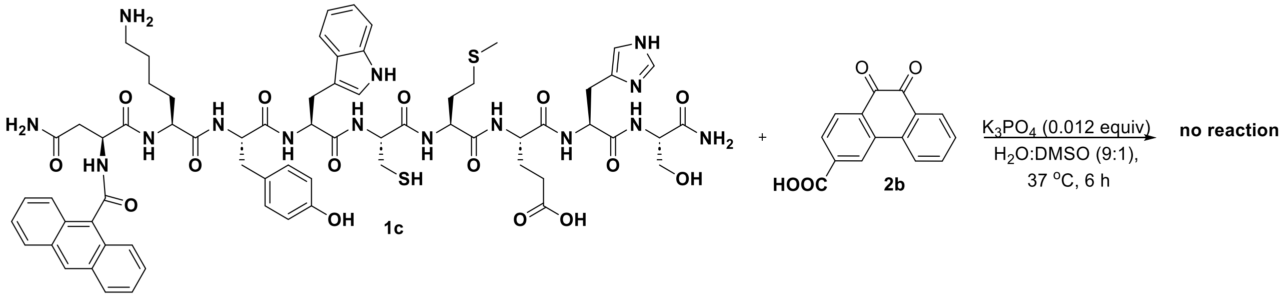

LCMS of Peptide 1c: LCMS: m/z 1400.5508 (calcd $[M+H]^+$ = 1400.5556), m/z 1401.5538 (calcd $[M+2]^+$ = 1401.5634), m/z 700.7795 (calcd $[(M+2)/2]^+$ = 700.7739), (HPLC analysis at 220 nm). Retention time in HPLC: 16.9.

HPLC trace of peptide 1c

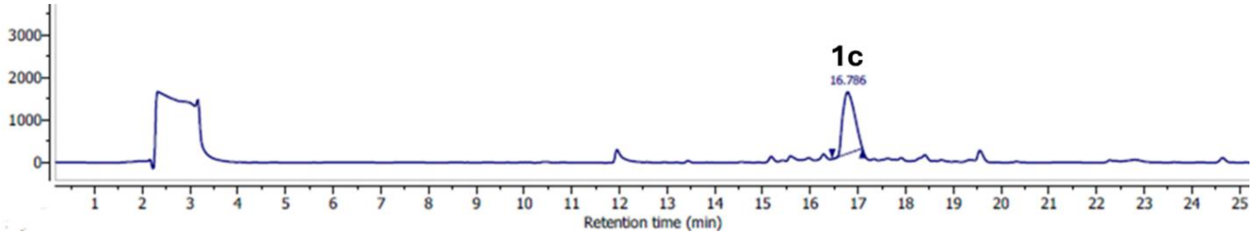

MS-trace of peptide 1c

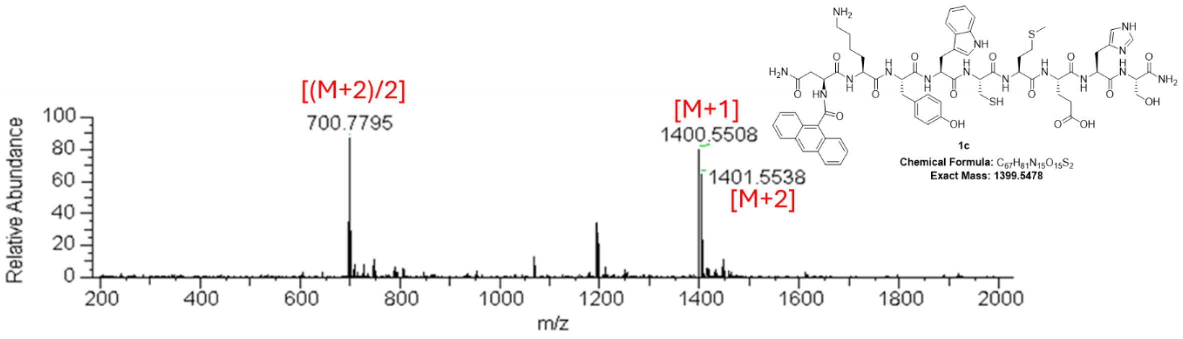

HPLC trace of reaction: peptide 1c with probe 2b

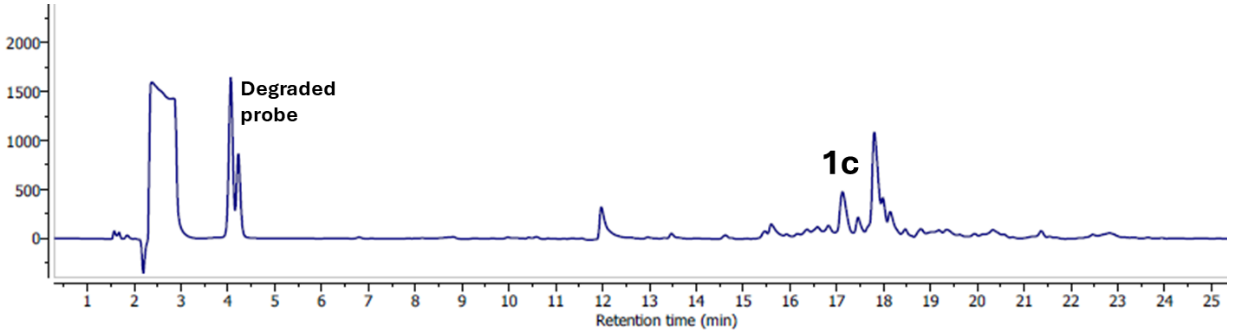

Figure. S9. Peptide substrate scope.

Reaction of peptide **1d** (**anthracene-HRW**) with 9,10 phenanthrenequinone analog **2f** was carried out using general procedure 1.

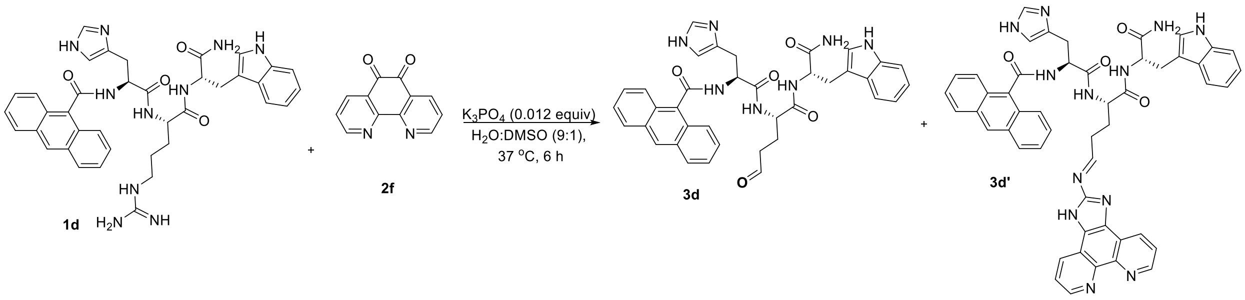

HPLC trace of peptide 1d

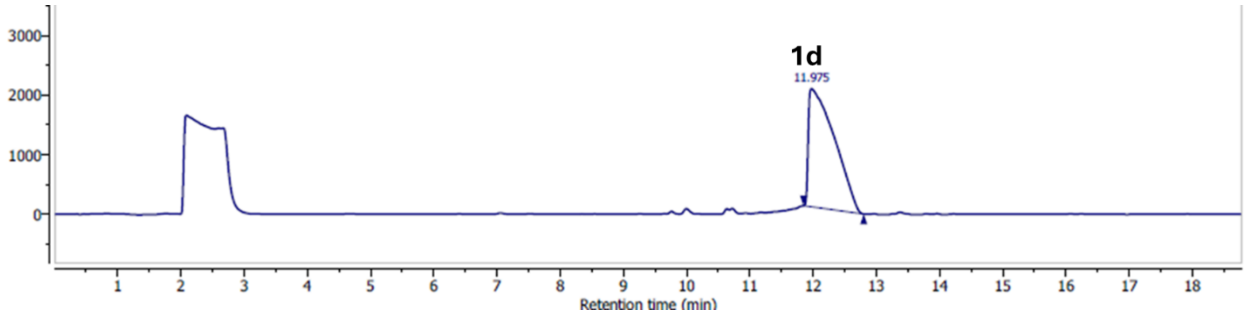

LCMS of Peptide 1d: LCMS: m/z 701.3306 (calcd $[M+H]^+$ = 701.3312), m/z 702.3334 (calcd $[M+2]^+$ = 702.3390), m/z 351.1689 (calcd $[(M+2)/2]^+$ = 351.1667), (HPLC analysis at 220 nm). Retention time in HPLC: 11.97.

MS-trace of peptide 1d

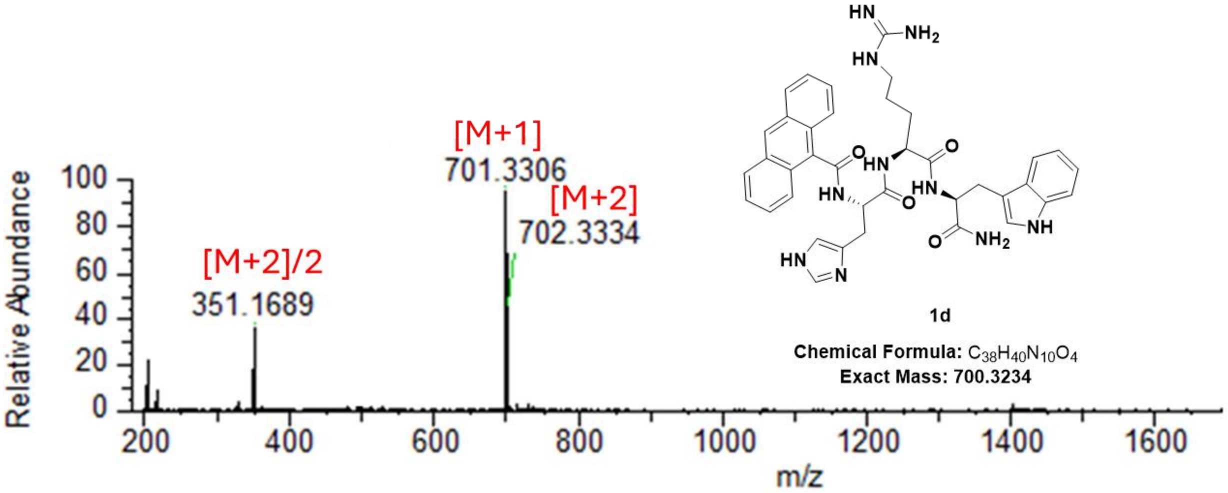

HPLC trace of reaction: peptide 1d with probe 2f

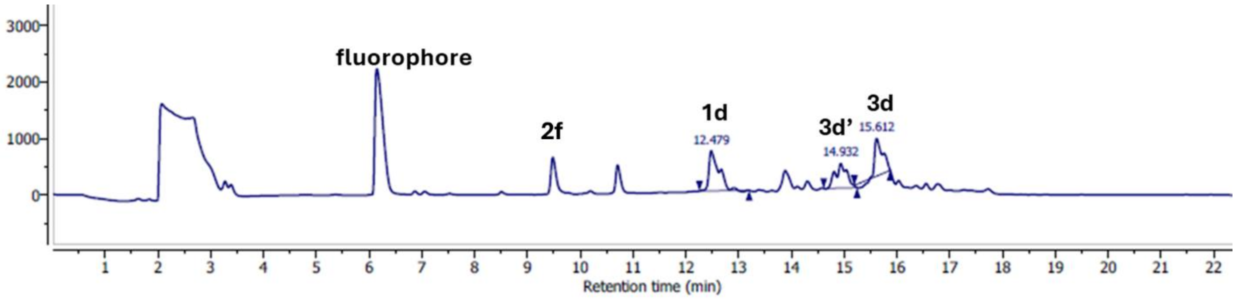

LCMS of Peptide 3d: LCMS: m/z 658.2772 (calcd $[M+H]^+$ = 658.2778), m/z 680.2589 (calcd $[M+Na]^+$ = 680.2597), (HPLC analysis at 220 nm). Retention time in HPLC: 15.612.

MS-trace of peptide 3d

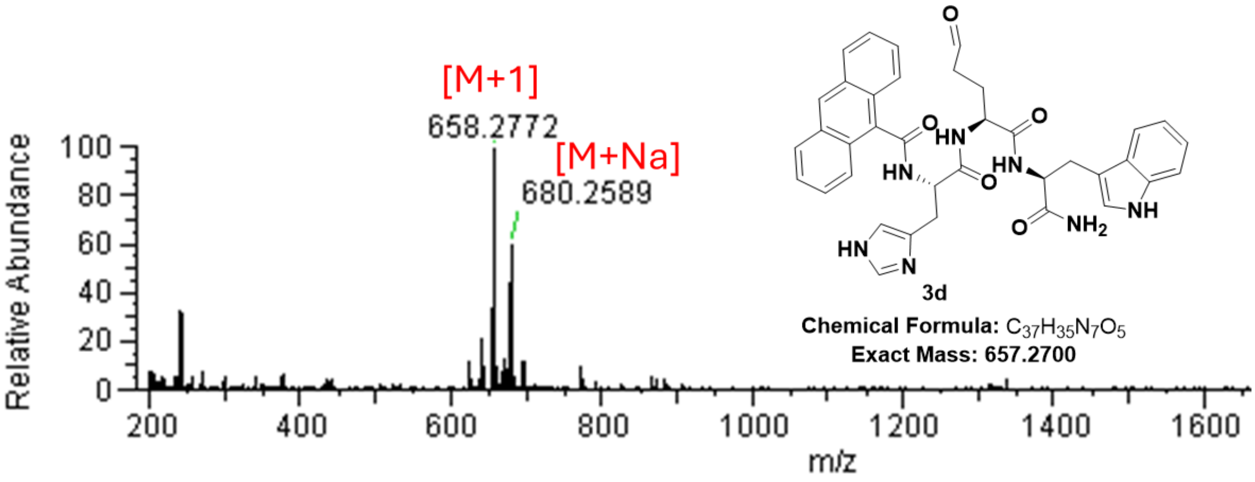

LCMS of Peptide 3d': LCMS: m/z 875.3523 (calcd $[M+H]^+$ = 875.3530), m/z 438.1798 (calcd $[(M+2)/2]^+$ = 438.1726), (HPLC analysis at 220 nm). Retention time in HPLC: 14.9.

MS-trace of peptide 3d'

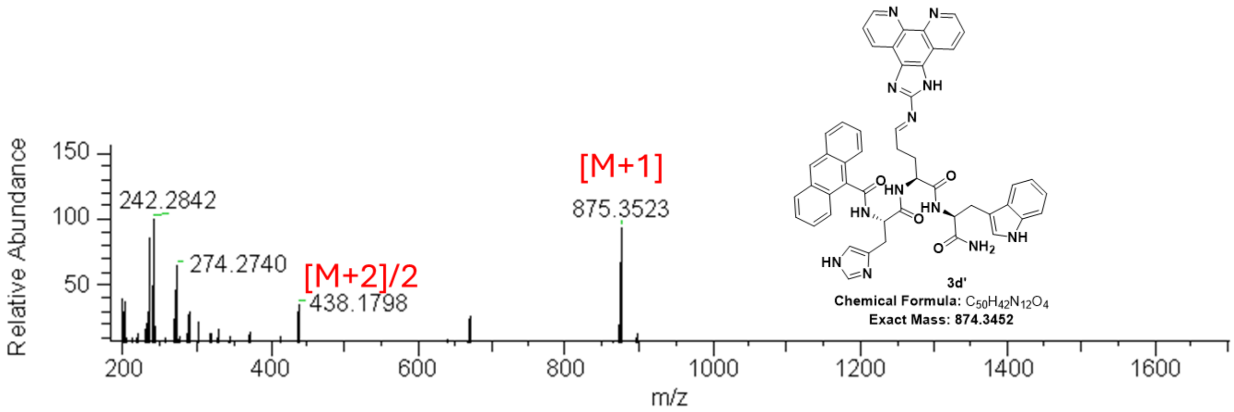

Figure S10. Protein optimization studies.

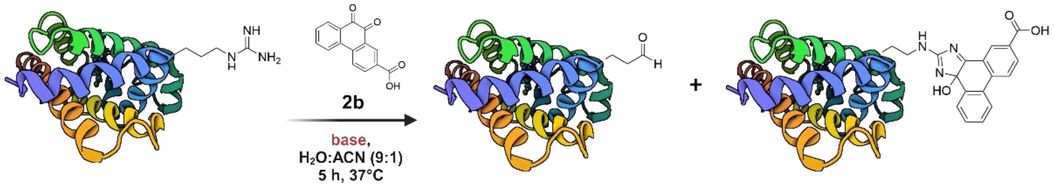

General Procedure: Myoglobin (equine heart) (2 mg, 240 µM) and 9,10-dihydrophenanthrene-3-carboxylic acid **2b** (0.06 mg, 480 µM) were dissolved in 500 µL of H_2O:ACN (9:1) solution in

the presence of different **bases.** Reaction was stirred at 37 °C for 5 h. Reactions were neutralized to pH 5 using 1 M HCl and purified using Amicron 3000 kDa molecular weight cutoffs filters. Modified proteins were resuspended in 0.1% formic acid in water and analyzed by LCMS. Aldehyde products are listed as modified; all conversions also include imine formation of aldehyde products. One condition showed a small conversion to the probe attached which is labeled as PA*. The four tested base conditions are shown below:

Entry	Probe	Base	Concentration
1	**2b**	NaOH	0.08 M
2	**2b**	NaOH	0.1 M
3	**2b**	DBU	13.4 x 10^{-3} M
4	**2b**	DBU	53.6 x 10^{-3} M

Results of Optimization Entry 1: 75% conversion

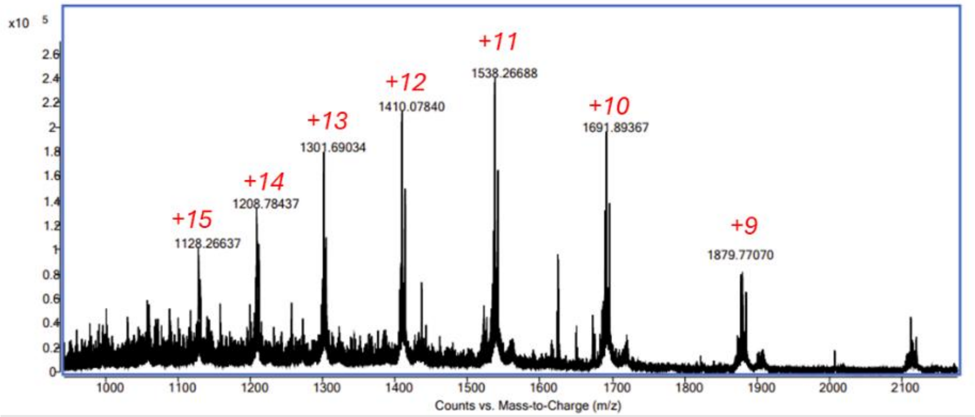

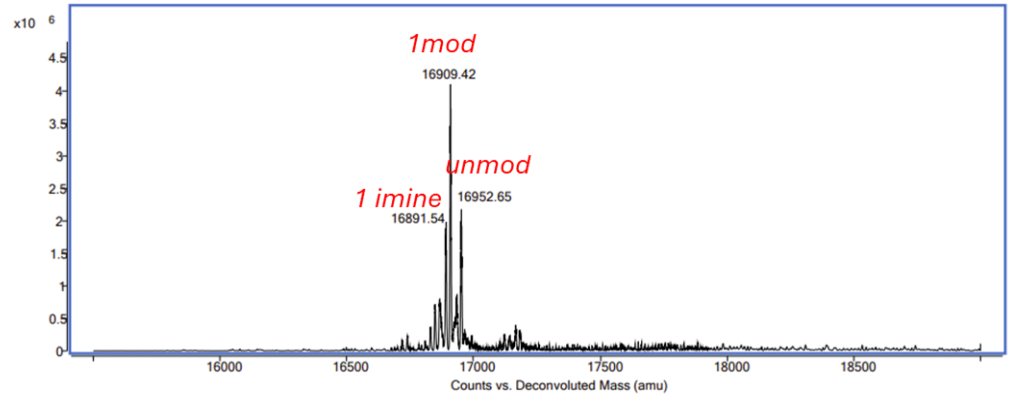

Results of Optimization Entry 2: 73% conversion

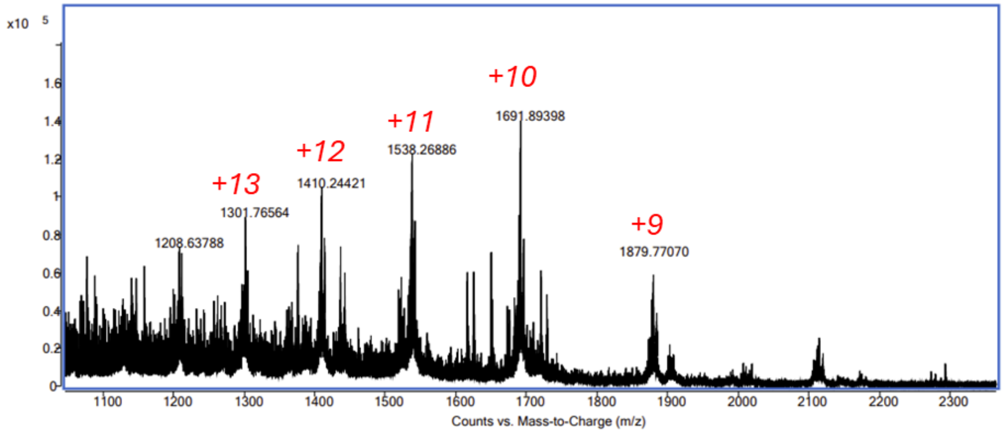

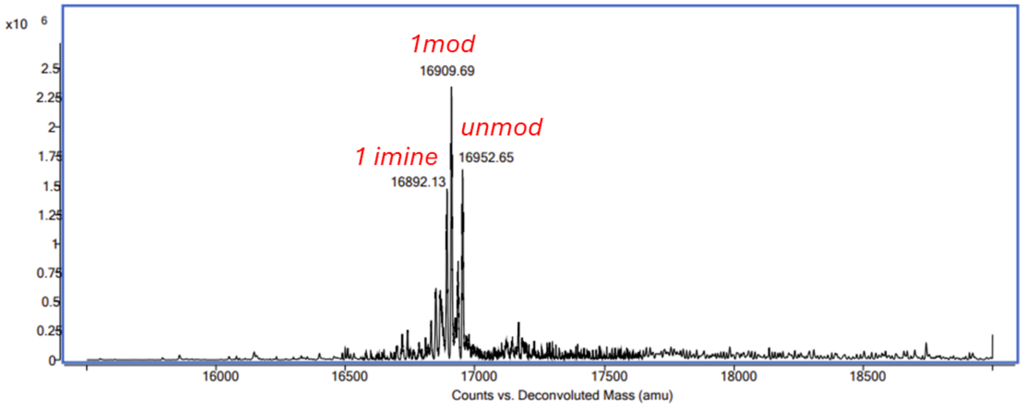

Results of Optimization Entry 3: 55% conversion to aldehyde, 13% conversion to probe attached

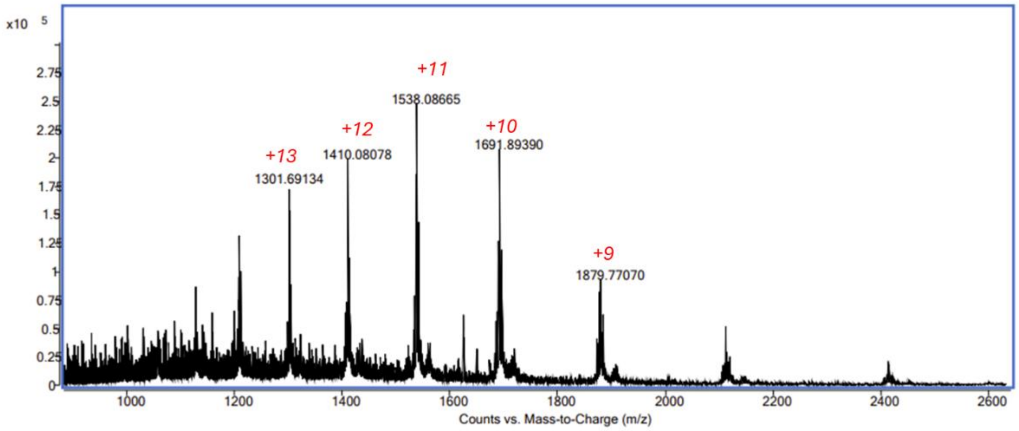

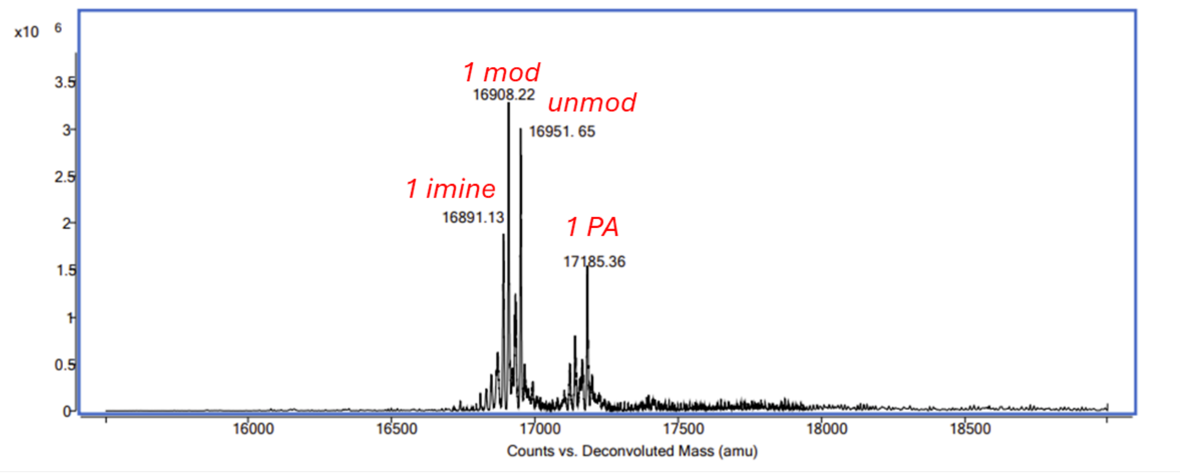

Results of Optimization Entry 4: 72% conversion

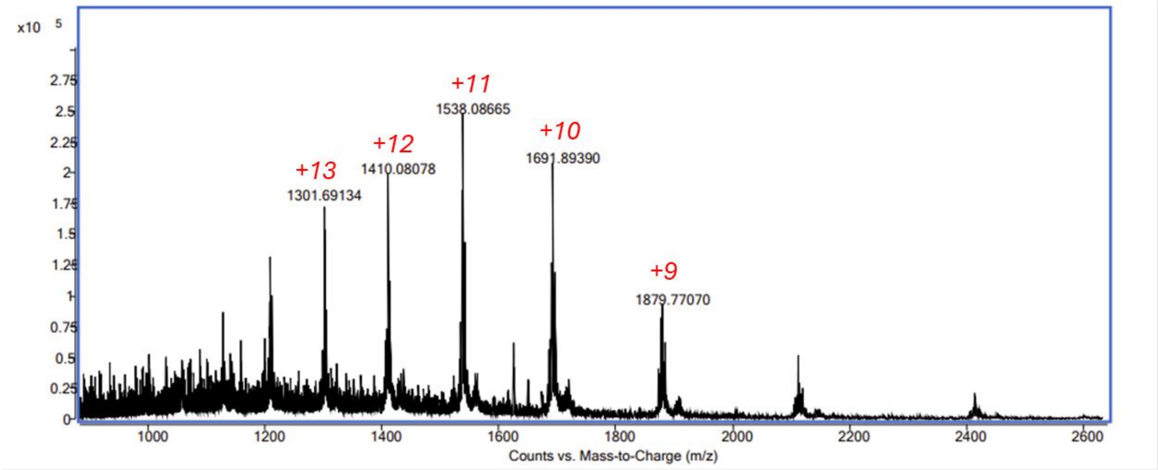

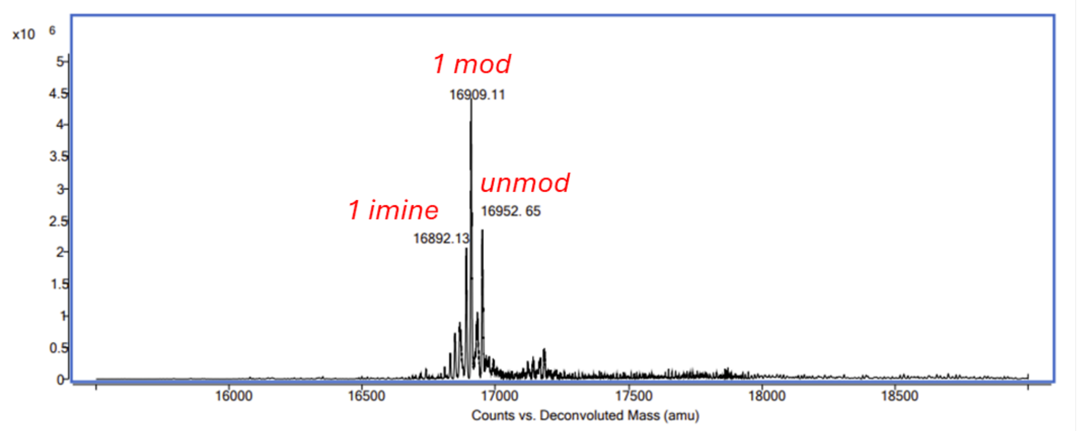

MS/MS analysis of modified digested Myoglobin (equine heart):

Myoglobin (equine heart) showed single modification at R31, below is 2 peptide spectra matches of this modification. Myoglobin contains 2 Arg residues.

*Identified Peptide Fragment (**1 site**): VEADIAGHGQEVLI**R**LFTGHPETLEKFDK (Sequence: AA 17-45, **R31**)*

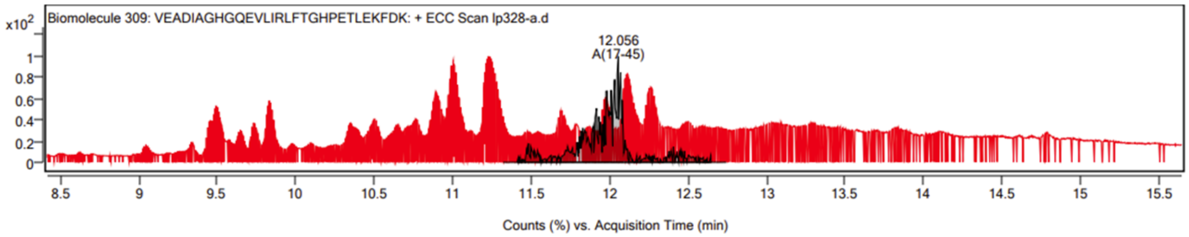

Mass Spectrum (with MFE spectrum, if available)

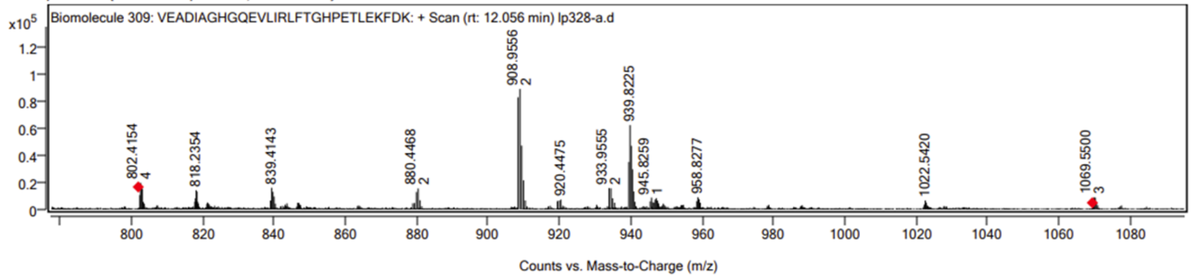

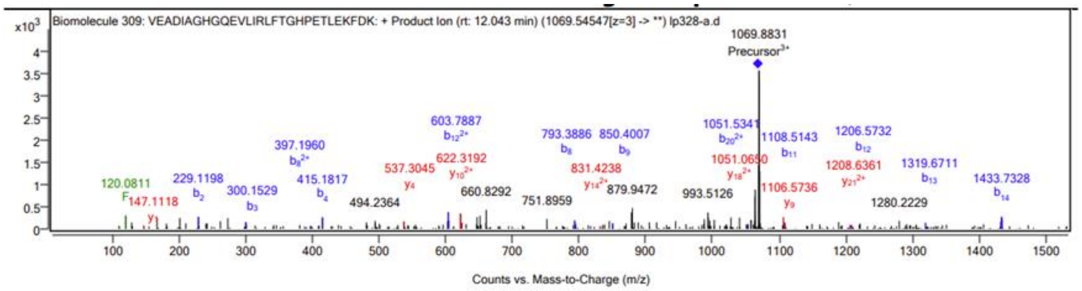

Fragment Spectrum Peaks

m/z	Diff (ppm)	Abund	Ion	Z
147.1118	6.63	75	y1	
537.3045	-2.52	167	y4	
1106.5736	-0.68	260	y9	
622.3192	0.41	346	y10	
831.4238	7.81	59	y14	
1051.0650	8.70	68	y18	
1208.6361	0.82	88	y21	
229.1198	-6.62	272	b2	
300.1529	8.22	154	b3	
415.1817	1.48	259	b4	
793.3886	-5.89	190	b8	
850.4007	5.50	126	b9	
1108.5143	-3.94	143	b11	
1206.5732	1.43	83	b12	
1319.6711	-9.14	139	b13	
1433.7328	9.53	276	b14	
397.1960	-1.04	60	b8	
603.7887	3.96	378	b12	
1051.5341	6.54	104	b20	
110.0723	-9.14	70	H	
120.0811	-3.03	301	F	
1069.5462	3.99	1426	Precursor	
1069.8831	1.71	3564	Precursor	
1070.2170	2.21	3534	Precursor	
1070.5559	-1.99	3318	Precursor	
1070.8855	2.58	1296	Precursor	

*Identified Peptide Fragment (**1 site**): ADIAGHGQEVLI**R**LFTGHPETLEK (Sequence: AA 19-42, **R31**)*

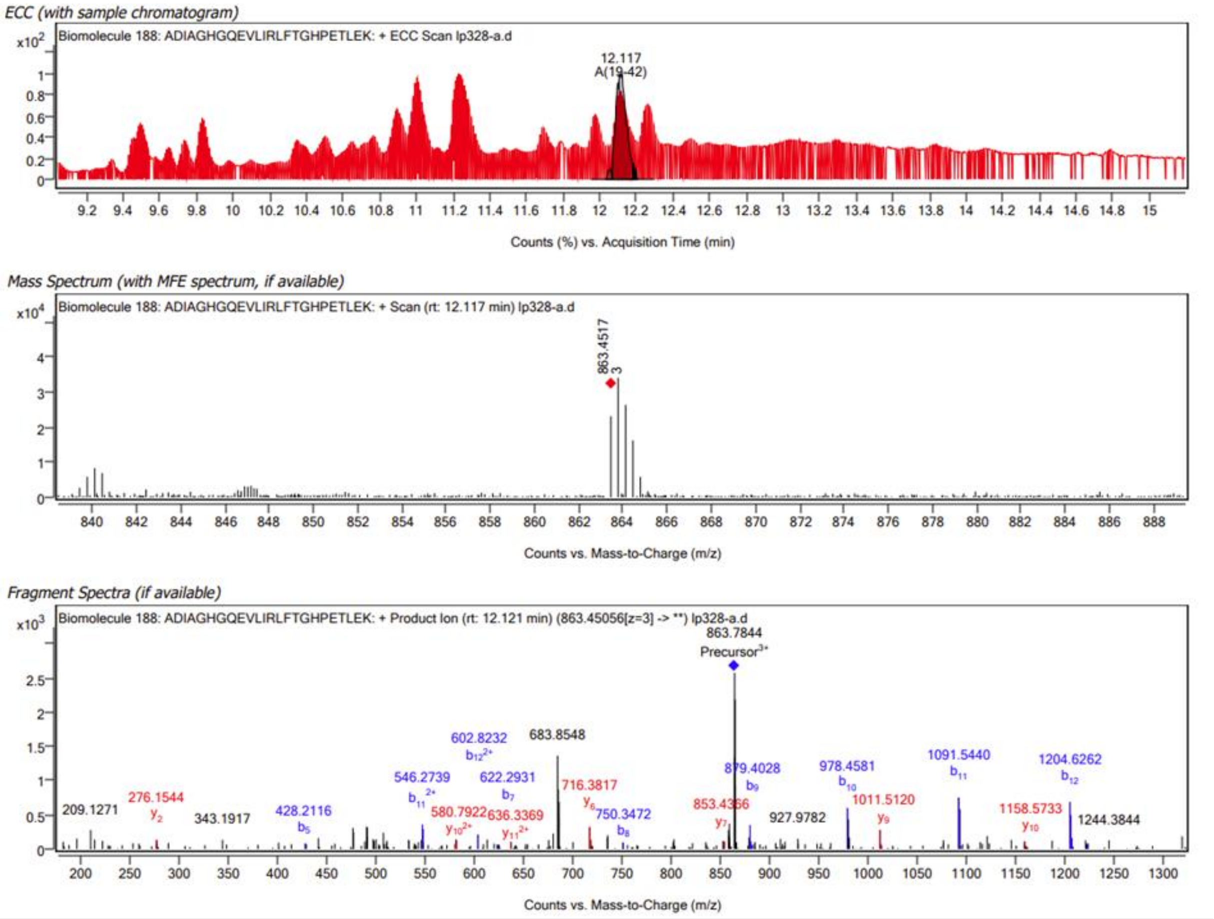

Fragment Spectrum Peaks

m/z	Diff (ppm)	Abund	Ion
276.1544	3.79	135	y2
716.3817	1.08	321	y6
853.4366	5.60	110	y7
1011.5120	-1.44	279	y9
1158.5733	4.87	112	y10
580.7922	7.44	129	y10
636.3369	-2.74	104	y11
428.2116	5.60	71	b5
622.2931	1.98	65	b7
750.3472	7.68	96	b8
879.4028	-8.24	349	b9
978.4581	5.98	599	b10
1091.5440	3.68	754	b11
1204.6262	4.88	689	b12
546.2739	6.78	359	b11
602.8232	-5.89	212	b12
1222.1298	-7.78	80	b23
863.4498	0.27	1335	Precursor
863.7844	0.03	2578	Precursor
864.1179	1.22	2186	Precursor
864.4496	4.33	938	Precursor

Modification of Aprotinin

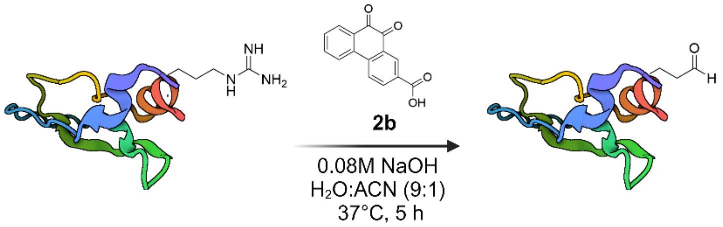

General Procedure: Aprotinin (0.8 mg, 240 μM) and 9,10-dihydrophenanthrene-3-carboxylic acid **2b** (0.06 mg, 480 μM) was dissolved in 500μL of H_2O:ACN (9:1) solution with 0.08 M NaOH. Reaction was stirred at 37 °C for 5 h. Reactions were neutralized to pH 5 with 1 M HCl and purified using Sigma 3000 kDa molecular weight cutoffs. Modified protein was resuspended in 0.1% formic acid in water and analyzed by LCMS. Aldehyde product is listed as modified, all conversions include aldehyde and imine product. The conversion was found to be >95% (+2 modification).

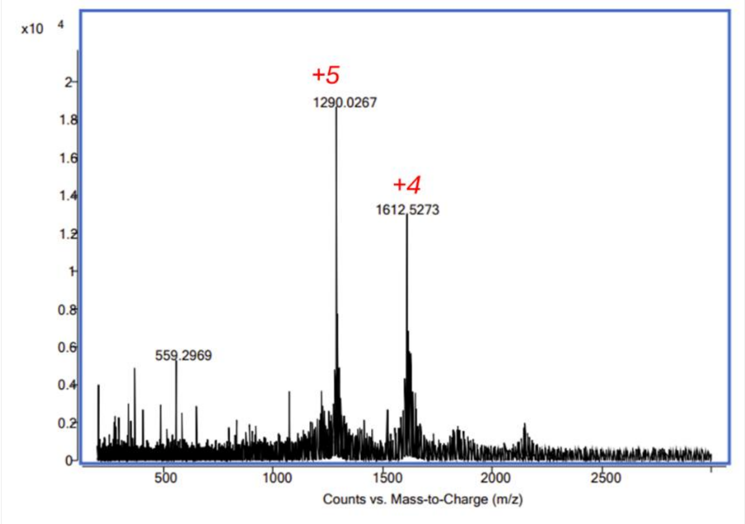

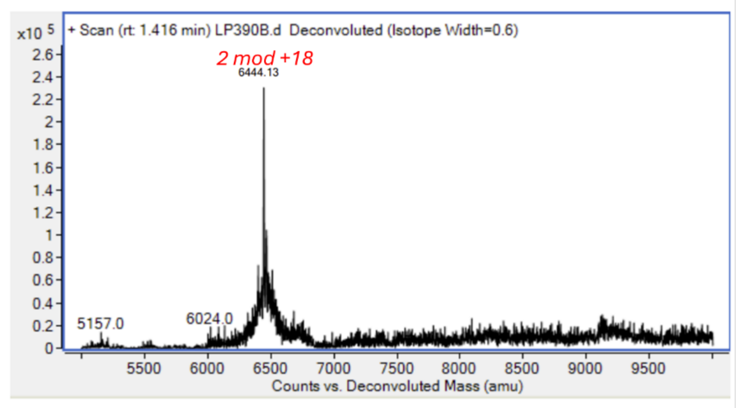

MS/MS analysis of digested modified Aprotonin:

Aprotinin showed two modifications at **R39** and **R53**, below is 2 peptide spectra matches for each site modification. Aprotonin has a total of 6 Arg residues.

*Identified Peptide Fragment (**1 site**): FVYGGC**R**AKR (Sequence: AA 33-42, **R39**).*

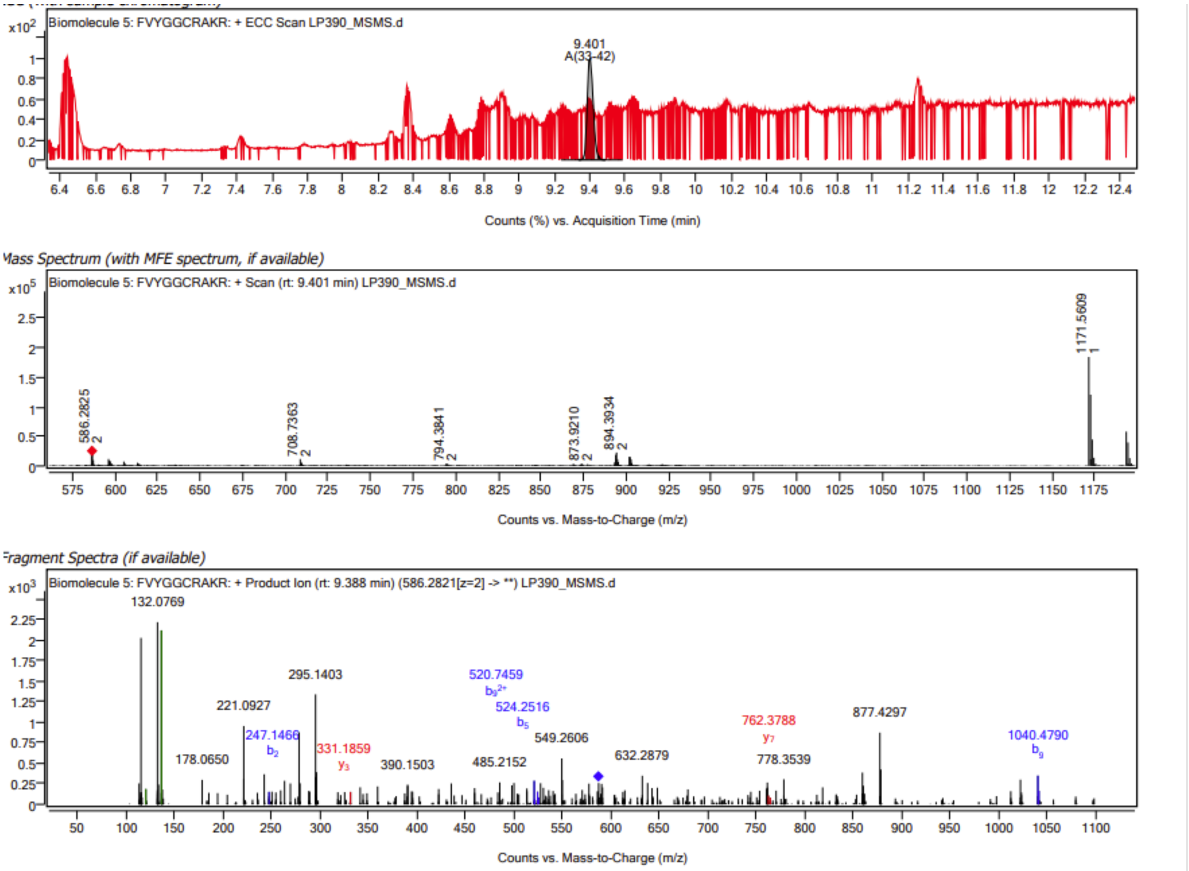

Fragment Spectrum Peaks

m/z	Diff (ppm)	Abund	Ion	Z
331.1859	35.32	151	y3	
762.3788	-29.50	107	y7	
247.1466	-10.10	152	b2	
524.2516	-2.40	154	b5	
1040.4790	18.46	350	b9	
520.7459	13.11	288	b9	
120.0791	14.36	187	F	
136.0769	-8.68	2124	Y	

*Identified Peptide Fragment (**1 site**): YNAKAGLCQTFVYGGC**R**: (Sequence: AA 23-39, **R39**)*

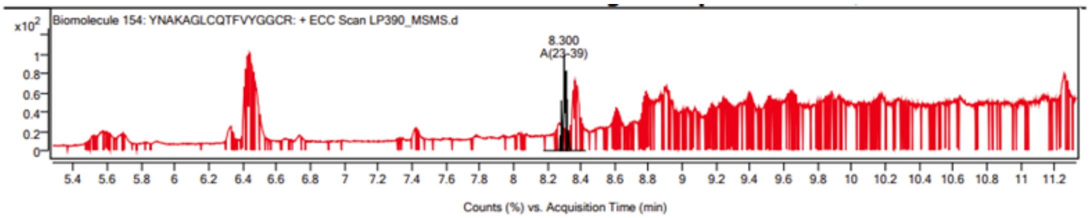

Mass Spectrum (with MFE spectrum, if available)

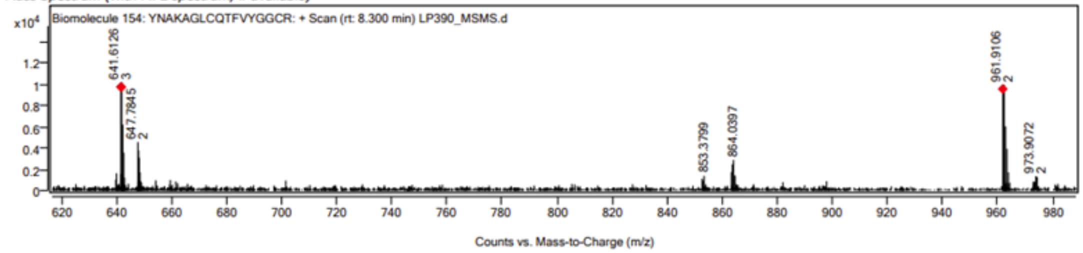

Fragment Spectra (if available)

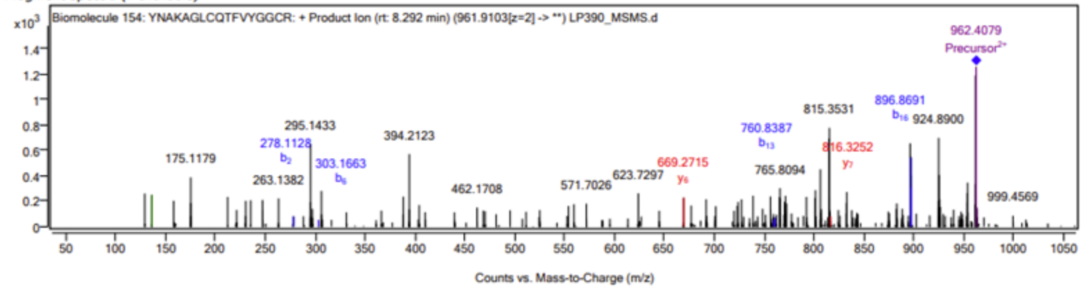

Fragment Spectrum Peaks

m/z	Diff (ppm)	Abund	Ion	Z
669.2715	-24.86	228	y6	
816.3252	-2.31	81	y7	
278.1128	2.78	81	b2	
303.1663	-34.94	53	b6	
760.8387	32.40	74	b13	
896.8691	20.12	545	b16	
136.0735	16.24	250	Y	
962.4079	8.62	1255	Precursor	

*Identified Peptide Fragment (**site 2**): NNFKSAEDCM**R**TCGG: (Sequence: AA 43-57, **R53**)*

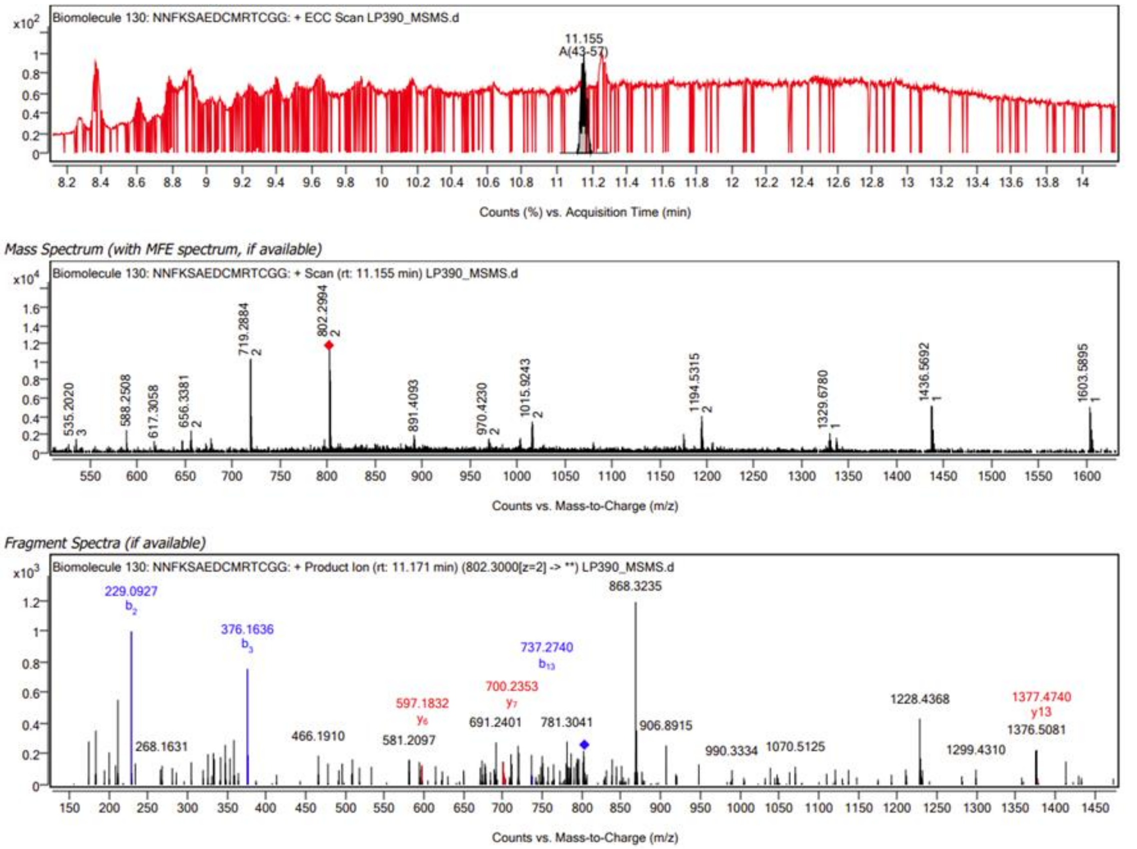

Fragment Spectrum Peaks

m/z	Diff (ppm)	Abund	Ion	Z
597.1832	29.29	127	y6	
1377.4740	27.55	42	y13	
229.0927	1.97	999	b2	
376.1636	-5.59	753	b3	
737.2740	2.47	53	b13	

*Identified Peptide Fragment (**site 2**): NNFKSAEDCM**R**TCG (Sequence: AA 43-56, **R53**)*

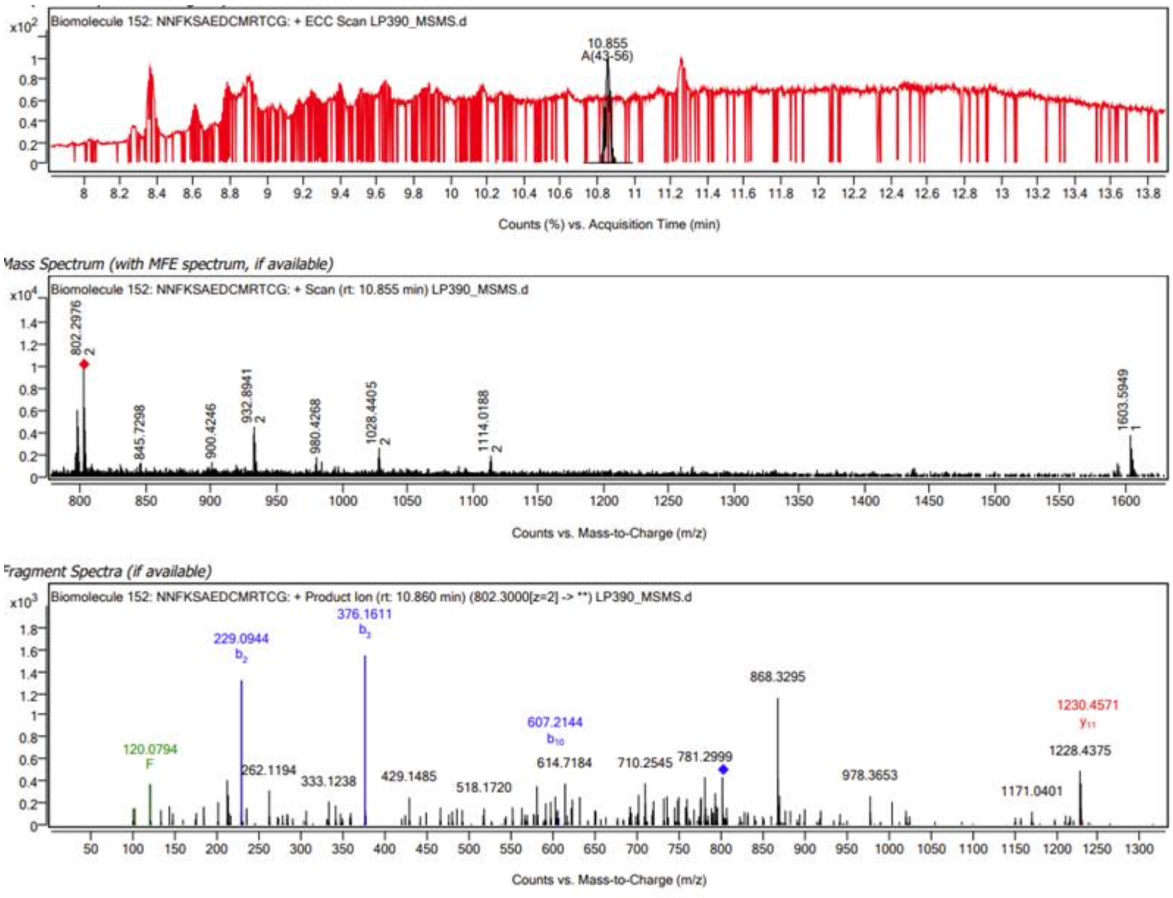

Fragment Spectrum Peaks

m/z	Diff (ppm)	Abund	Ion	Z
1230.4571	-10.98	49	y11	
229.0944	-5.34	1318	b2	
376.1611	1.25	1545	b3	
607.2144	32.66	61	b10	
102.0527	21.93	147	E	
120.0794	11.54	370	F	

Procedure for homogenous modification of aprotinin: Aprotinin (0.8 mg, 240 µM) and 9,10-dihydrophenanthrene-3-carboxylic acid 2b (0.045 mg, 312 µM) was dissolved in 400 µL of H_2O:ACN (9:1) solution with 0.08 M NaOH. Reaction was stirred at 37 °C for 3 h. Reactions were neutralized to pH 5 with 1 M HCl and purified using Sigma 3000 kDa molecular weight cutoffs. Modified protein was resuspended in 0.1% formic acid in water and analyzed by LCMS. Aldehyde product is listed as modified. The conversion was found to be > 70% (+1 modification).

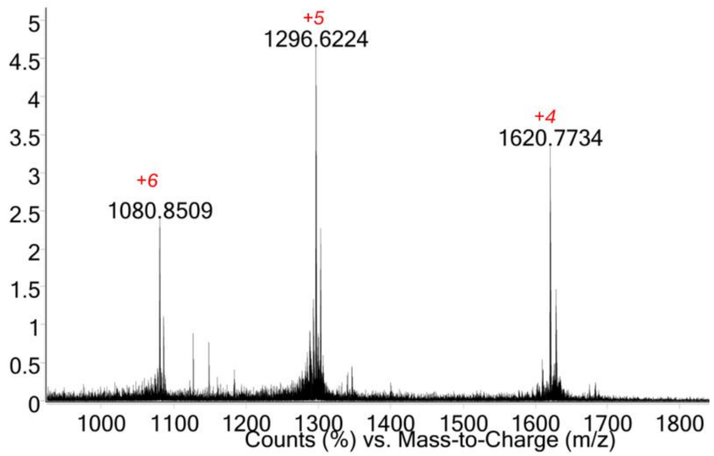

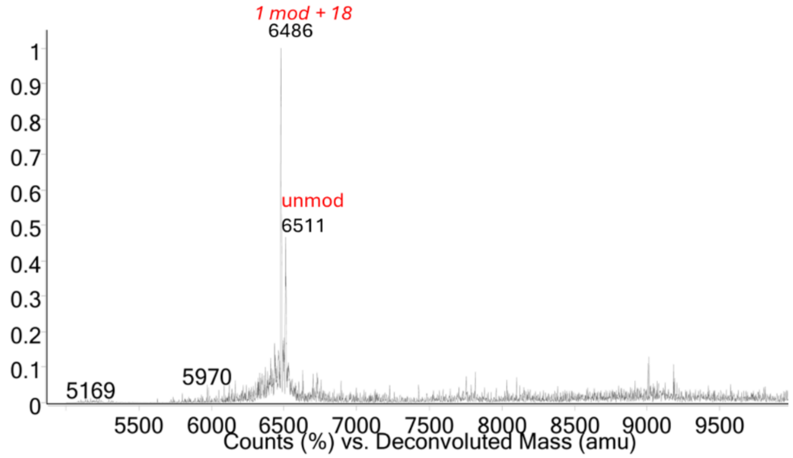

Ubiquitin Modification

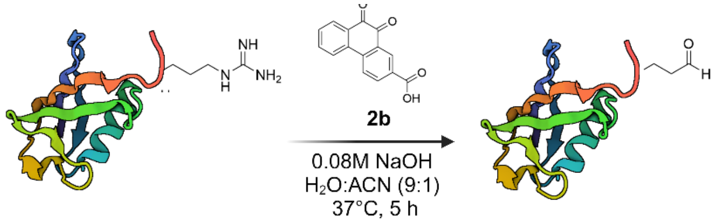

General Procedure: Ubiquitin (1 mg, 240 µM) and 9,10-dihydrophenanthrene-3-carboxylic acid **2b** (0.06 mg, 480 µM) was dissolved in 500µL of H_2O:ACN (9:1) solution with 0.08 M NaOH. Reaction was stirred at 37 °C for 5 h. Reaction was neutralized to pH 5 with 1 M HCl and purified using Sigma 3000 kDa molecular weight cutoffs. Modified protein was resuspended in 0.1% formic acid in water and analyzed by LCMS. Aldehyde products are listed as modified, all conversions include aldehyde and imine product. The conversion was found to be >68% (+1 modification).

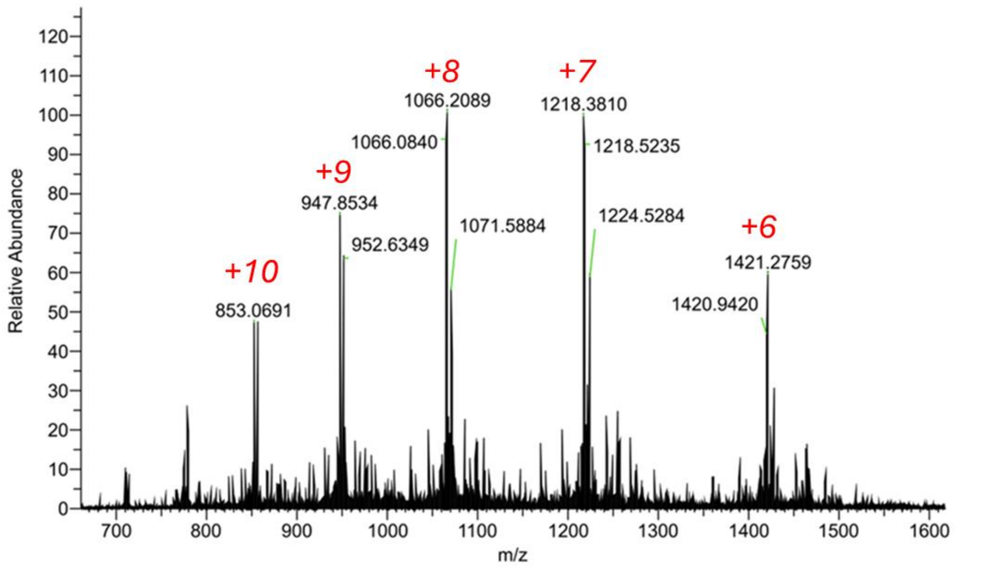

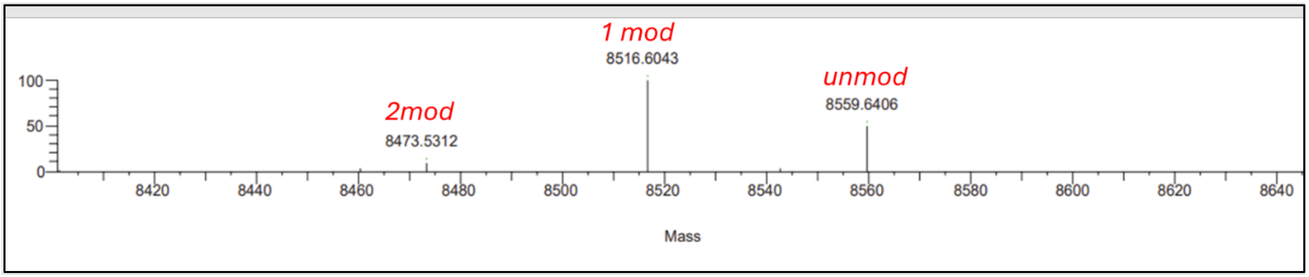

MS/MS analysis of digested modified Ubiquitin:

Ubiquitin showed to have modification at 2 sites to sum up a single modification. Modification was observed at positions **R42** and **R54**. There are a total 4 Arg in Ubiquitin.

*Identified Peptide Fragment (**1 site**): EGIPPDQQ**R**LIFAGK (Sequence: AA 34-48, **R42**)*

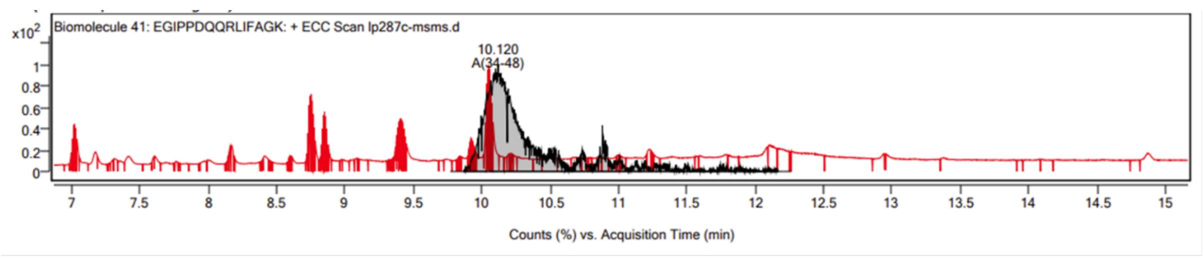

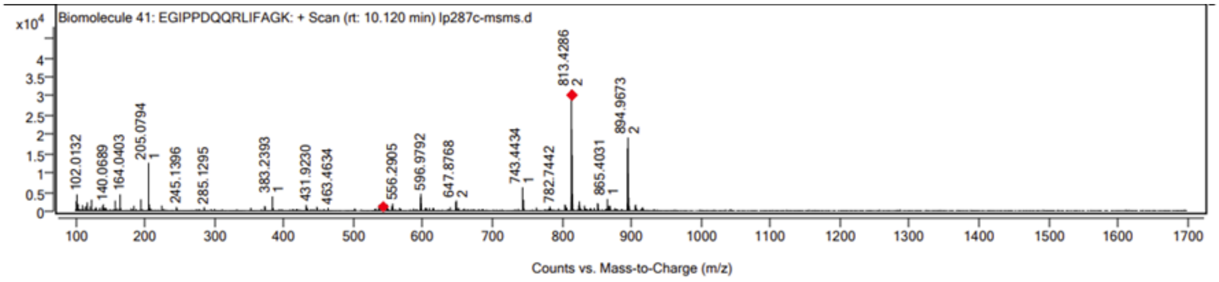

Fragment Spectra (if available)

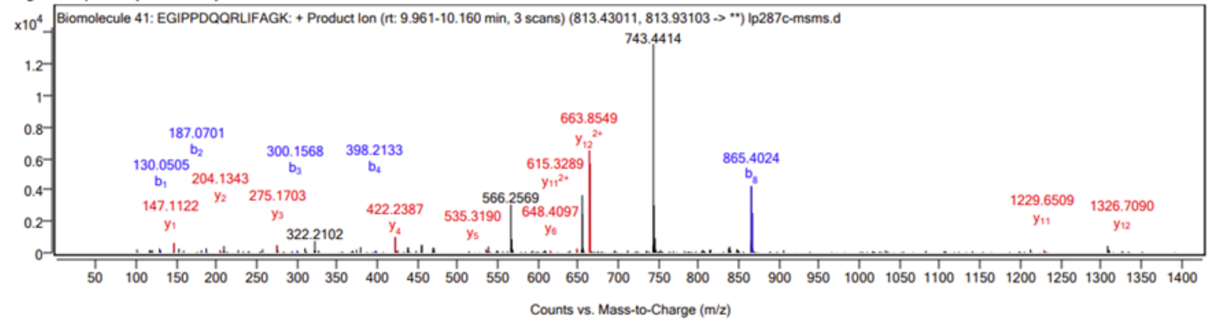

Fragment Spectrum Peaks

m/z	Diff (ppm)	Abund	Ion	Z
147.1122	4.01	609	y1	
204.1343	-0.07	132	y2	
275.1703	3.96	472	y3	
422.2387	2.53	993	y4	
535.3190	9.05	217	y5	
648.4097	-2.80	245	y6	
1229.6509	1.25	155	y11	
1326.7090	-2.84	80	y12	
615.3289	1.55	114	y11	
663.8549	2.07	6517	y12	
130.0505	-4.59	146	b1	
187.0701	6.65	99	b2	
300.1568	-4.77	137	b3	
398.2133	-4.38	114	b4	
865.4024	3.00	4241	b8	
101.0700	9.25	187	Q	
120.0797	8.87	162	F	
813.4237	8.17	152	Precursor	
813.9321	-0.14	125	Precursor	
814.4265	8.79	199	Precursor	

*Identified Peptide Fragment (**1 site**): QLEDG**R**TLSDYNIQK (Sequence: AA 49-63, **R54**)*

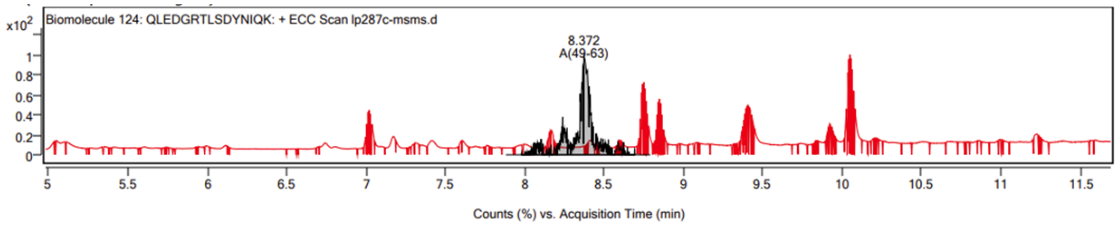

Mass Spectrum (with MFE spectrum, if available)

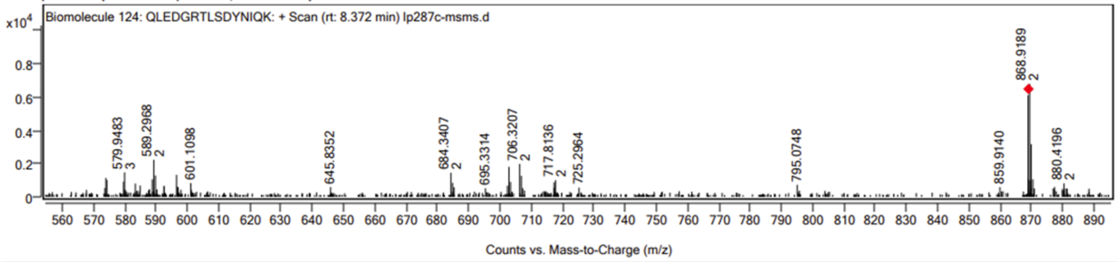

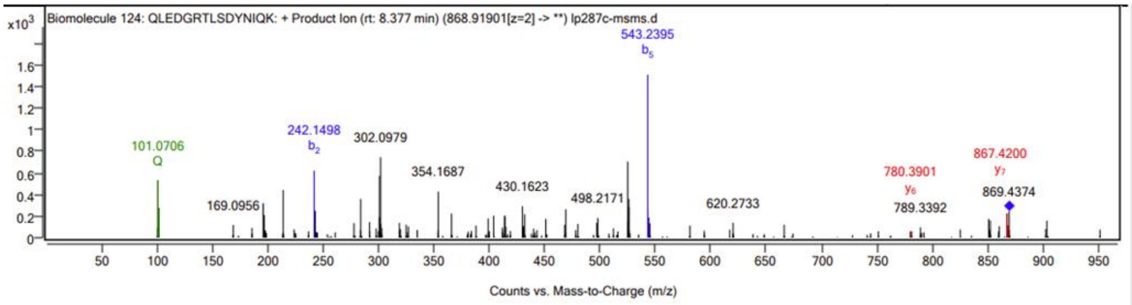

Fragment Spectrum Peaks

m/z	Diff (ppm)	Abund	Ion	Z
780.3901	-1.87	58	y6	
867.4200	0.80	226	y7	
242.1498	0.45	621	b2	
543.2395	2.59	1507	b5	
101.0706	3.69	533	Q	
102.0543	6.00	274	E	

Modification of RNAse

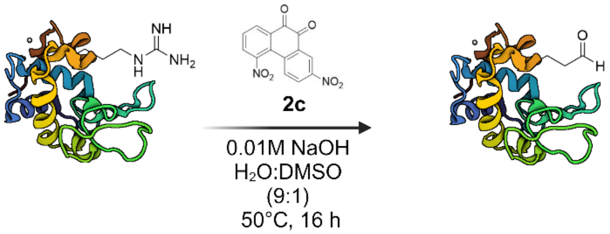

General Procedure: RNAse (1 mg, 70 µM) and 2,5-dinitrophenanthrene-9,10-dione **2c** (0.08 mg, 280 µM) was dissolved in 1 mL of H_2O:DMSO(9:1) solution with 0.01 M NaOH. Reaction was stirred at 50 °C for 16 h. Reaction was neutralized to pH 5 with 1 M HCl and purified using Sigma 3000 kDa molecular weight cutoffs. Modified protein was resuspended in 0.1% formic acid in water and analyzed by LCMS. Aldehyde products are listed as modified, all conversions include aldehyde and imine product. The conversion was found to be ~70% (+1, +2 and +3 modification).

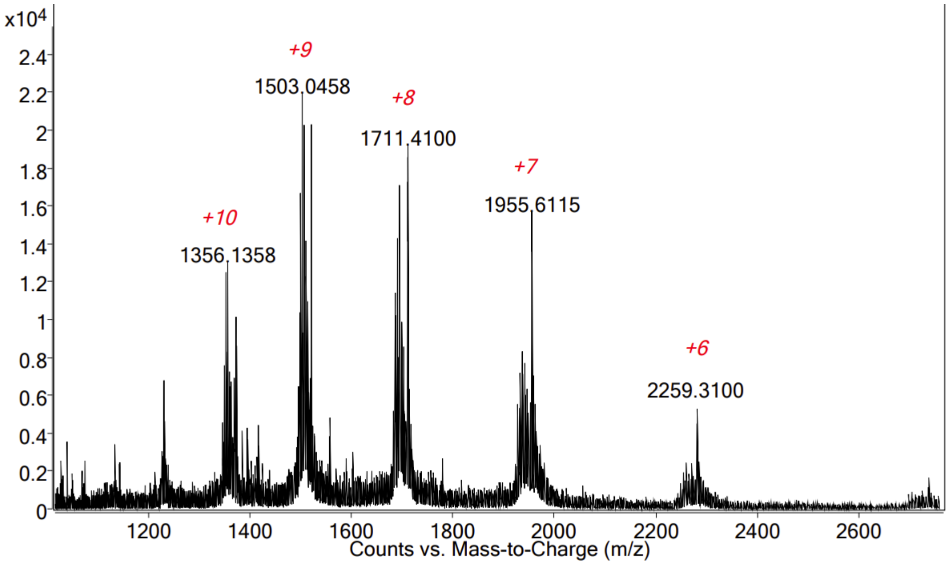

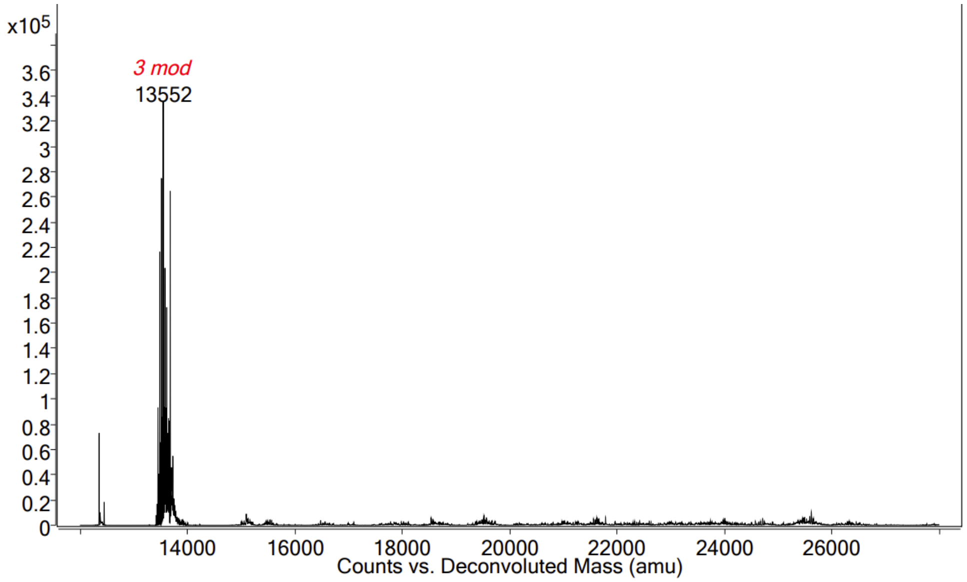

Lysozyme (chicken) modification

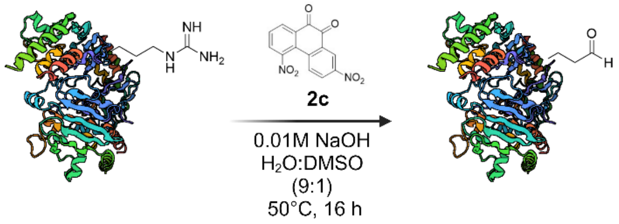

General Procedure: Lysozyme (chicken) (1 mg, 70 µM) and 2,5-dinitrophenanthrene-9,10-dione **2c** (0.08 mg, 280 µM) was dissolved in 1 mL of H_2O:DMSO(9:1) solution with 0.01 M NaOH. Reaction was stirred at 50 °C for 16 h. Reaction was neutralized to pH 5 with 1 M HCl and purified using Sigma 3000 kDa molecular weight cutoffs. Modified protein was resuspended in 0.1% formic acid in water and analyzed by LCMS. Aldehyde products are listed as modified, all conversions include aldehyde and imine product. The conversion was found to be >95% (+1, +2, +3 and +4 modification).

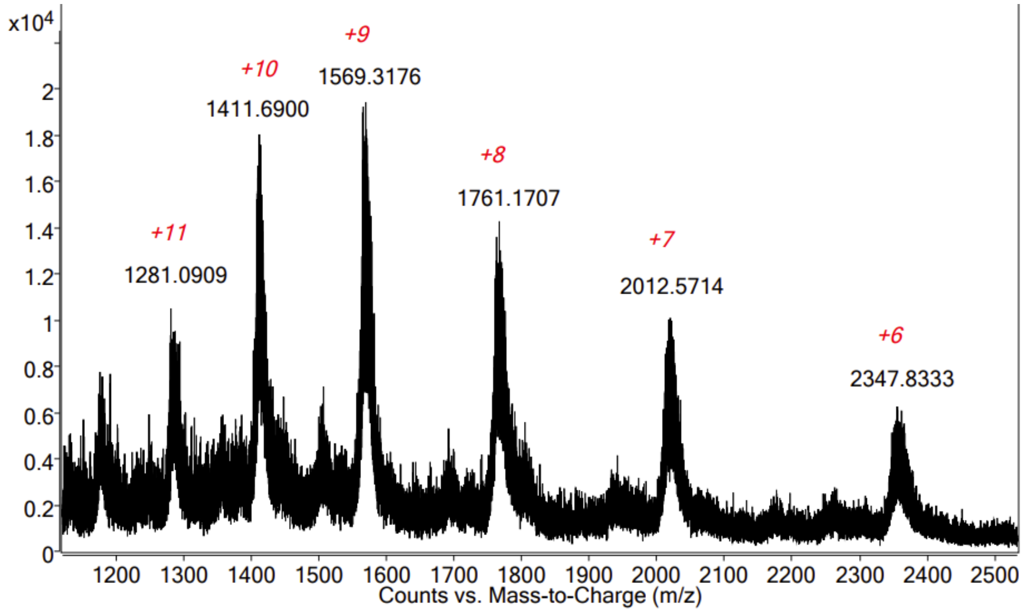

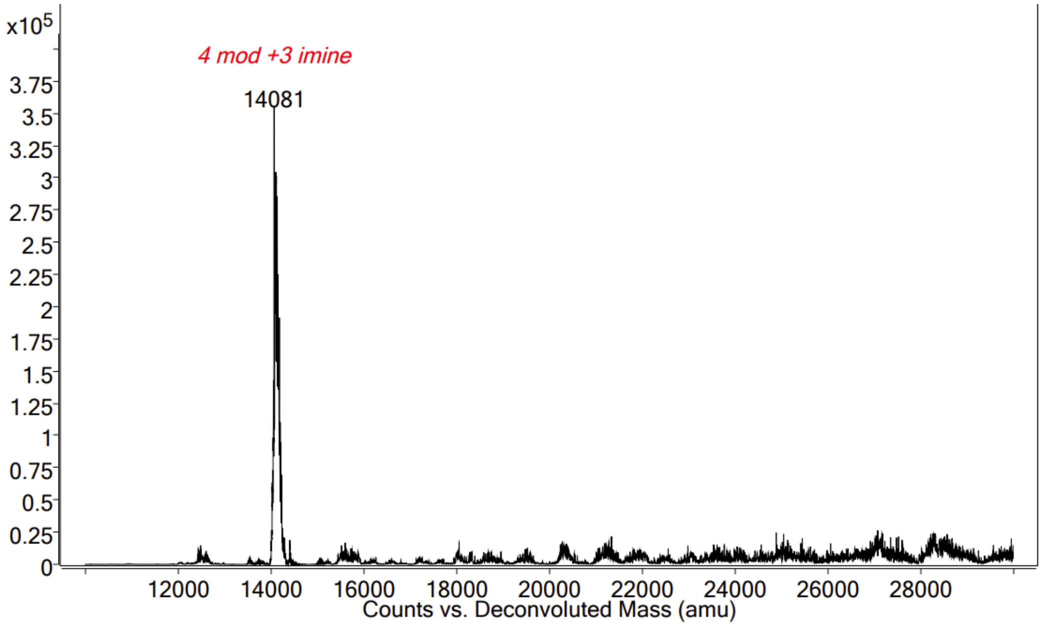

Modification of Chymotrypsinogen

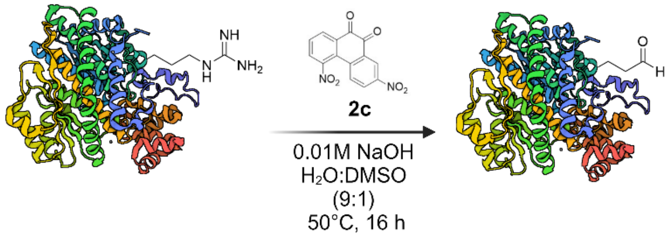

General Procedure: Chymotrypsinogen (1.8 mg, 70 µM) and 2,5-dinitrophenanthrene-9,10-dione **2c** (0.08 mg, 280 µM) was dissolved in 1 mL of H_2O:DMSO(9:1) solution with 0.01 M NaOH. Reaction was stirred at 50 °C for 16 h. Reaction was neutralized to pH 5 with 1 M HCl and purified using Sigma 3000 kDa molecular weight cutoffs. Modified protein was resuspended in 0.1% formic acid in water and analyzed by LCMS. Aldehyde products are listed as modified, all conversions include aldehyde and imine product. The conversion was found to be >95% (+1, +2 and +3 modification).

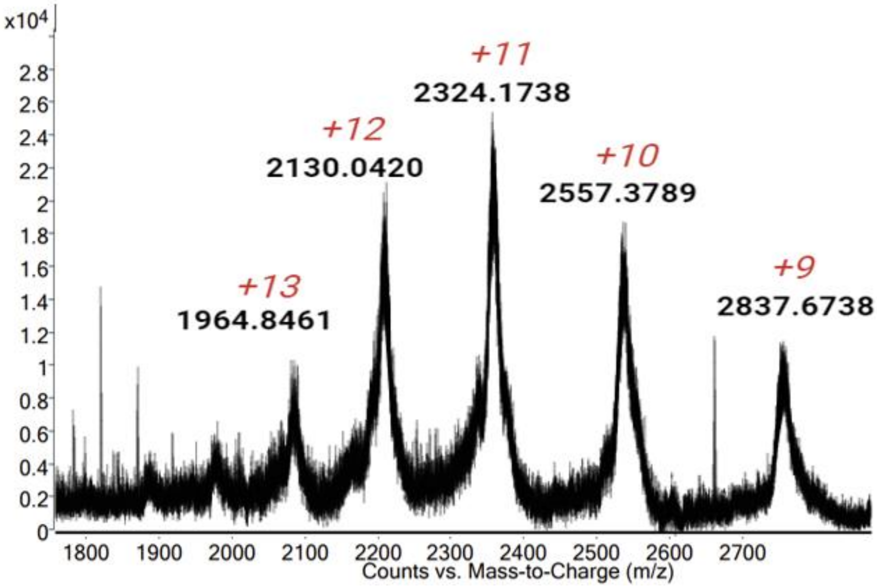

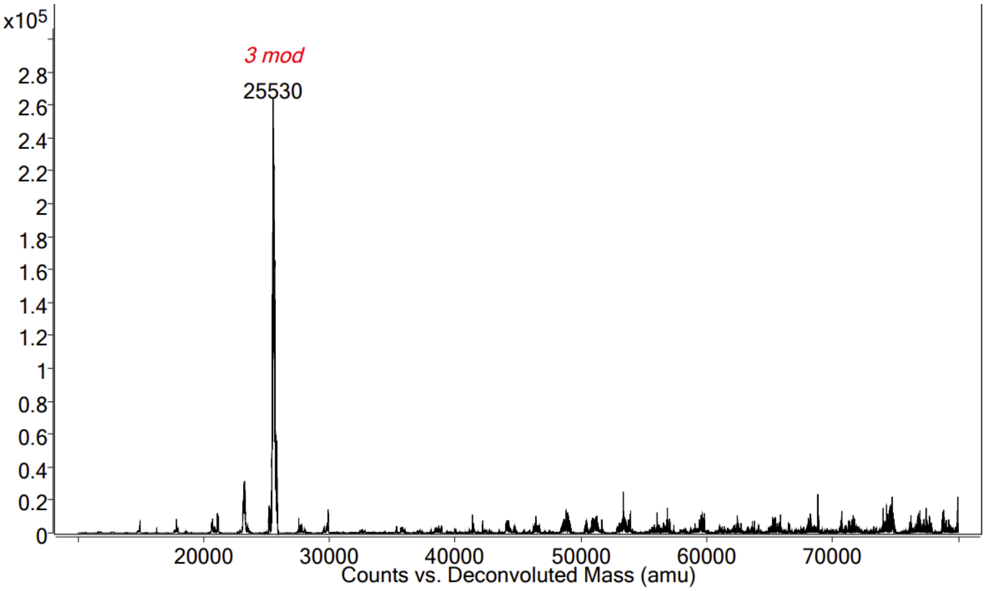

Modification of Apotransferrin (human)

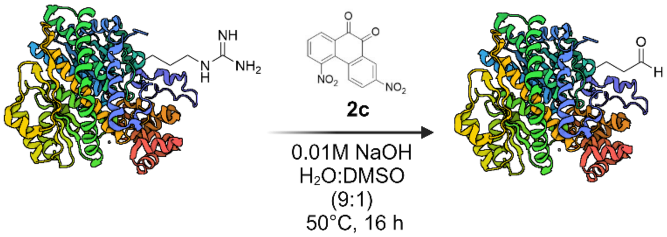

General Procedure: Apotransferrin (human) (5.6 mg, 70 µM) and 2,5-dinitrophenanthrene-9,10-dione **2c** (0.08 mg, 280 µM) was dissolved in 1 mL of H_2O:DMSO(9:1) solution with 0.01 M NaOH. Reaction was stirred at 50 °C for 16 h. Reaction was neutralized to pH 5 with 1 M HCl and purified using Sigma 3000 kDa molecular weight cutoffs. Modified protein was resuspended in 0.1% formic acid in water and analyzed by LCMS. Aldehyde products are listed as modified, all conversions include aldehyde and imine product. The conversion was found to be >95% (+1, +2, +3 and +4 modification).

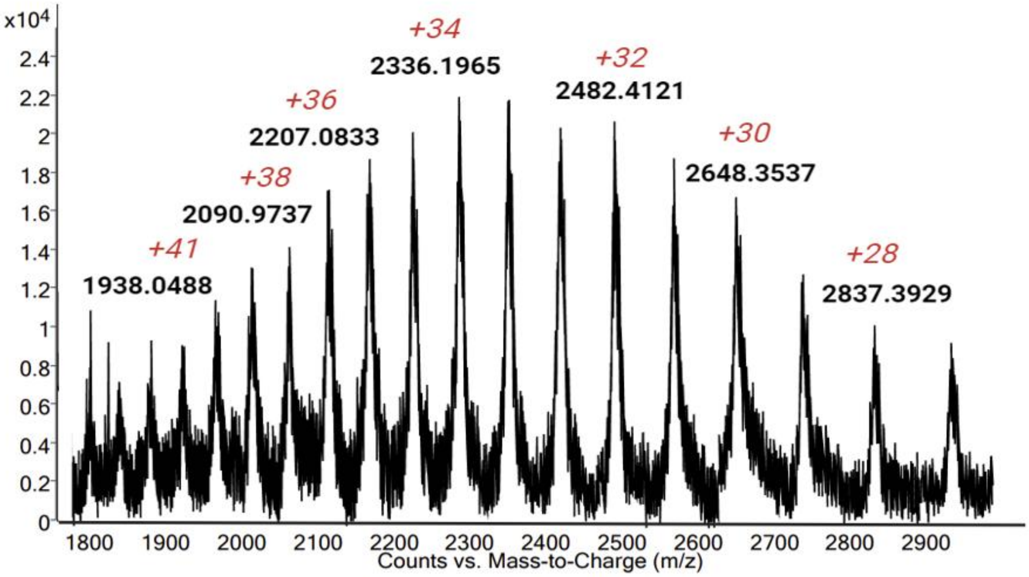

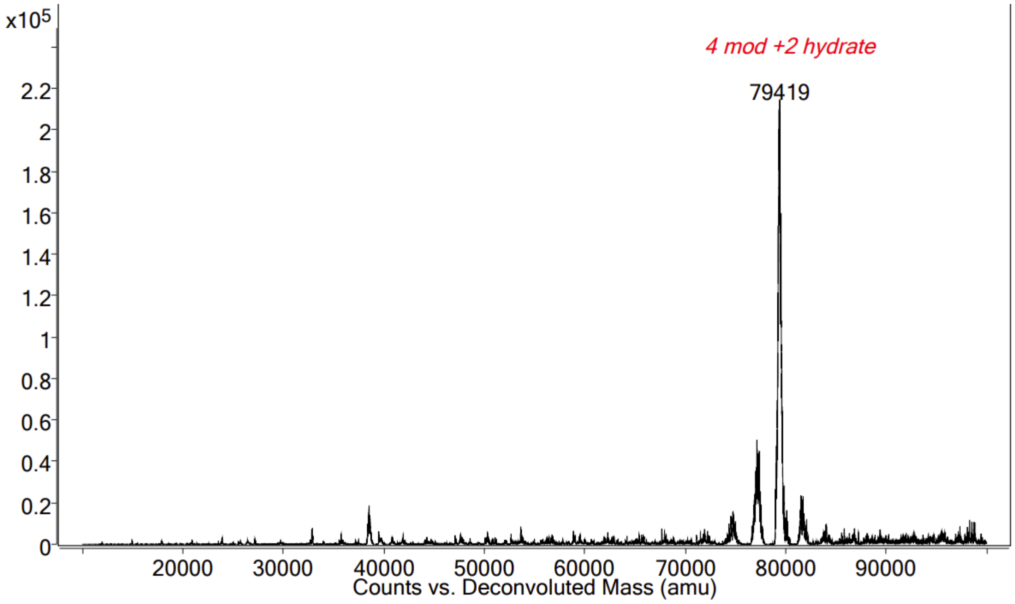

Procedure for homogenous modification of Cytochrome c: Cytochrome c (2 mg, 323 µM) and 2,5-dinitrophenanthrene-9,10-dione 2c (0.07 mg, 470 µM) was dissolved in 500 mL of H2O:DMSO(9:1) solution with 0.04 M NaOH. Reaction was stirred at 45 °C for 7 h. Reaction was neutralized to pH 5 with 1 M HCl and purified using Sigma 3000 kDa molecular weight cutoffs. Modified protein was resuspended in 0.1% formic acid in water and analyzed by LCMS. Aldehyde products are listed as modified. The conversion was found to be >60% (+1 modification).

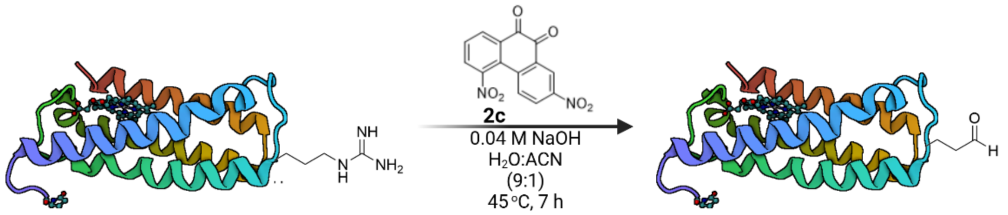

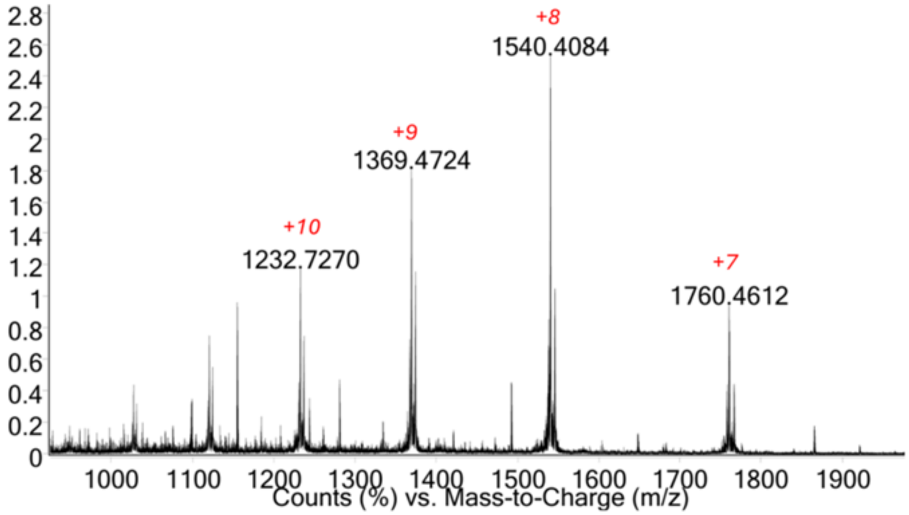

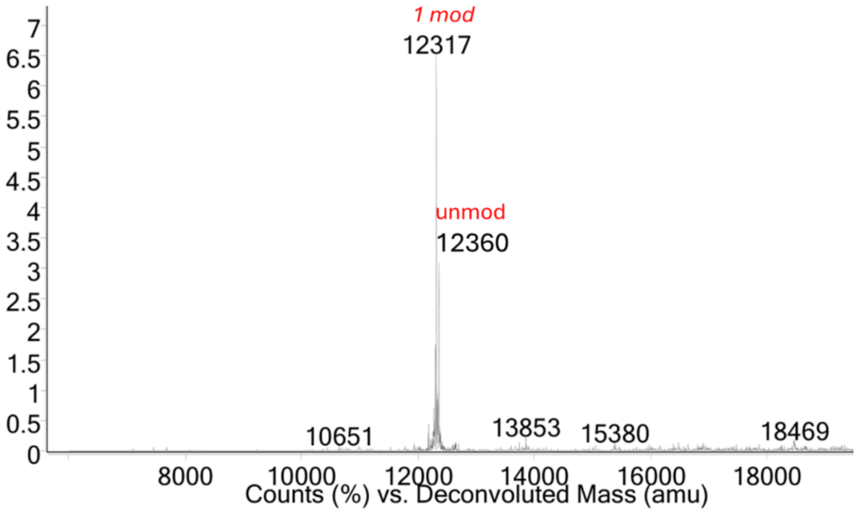

Small molecule analysis of Imine and Hydrate Formation with Aldehyde

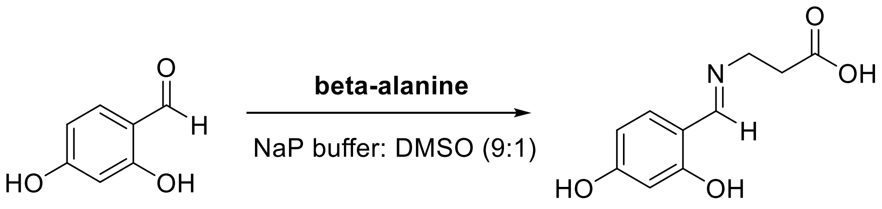

Procedure: 2,4 dihydroxybenzaldehyde (10 mg, 0.07 mmol) was dissolved in 50 microliters of DMSO-d6 and diluted to 600µL total volume with 20 mM Phosphate buffer. Beta-alanine (4 mg, 0.36 mmol) was then added, and the solution was allowed to stir at room temperature for two hours. 20 mM phosphate

buffer was prepared by dissolving 40 mg of dibasic sodium phosphate and 7 mg of monobasic sodium phosphate in 10 mL of D_2O. Various pH conditions of the phosphate buffer was prepared by adding 0.1M HCl for pH 4.5 buffer, and varying amounts of 0.8M NaOH for pH 9 and pH 12.

^{1}H NMR of 2,4 dihydroxybenzaldehyde and beta-alanine in pH 4.5 NaP buffer (27% imine)

^{1}H NMR of 2,4 dihydroxybenzaldehyde

(400 MHz, D_2O) δ 9.59 (s, 1H), 7.50 (s, 1H), 6.49 (d, *J* = 8.3 Hz, 1H), 6.30 (s, 1H).

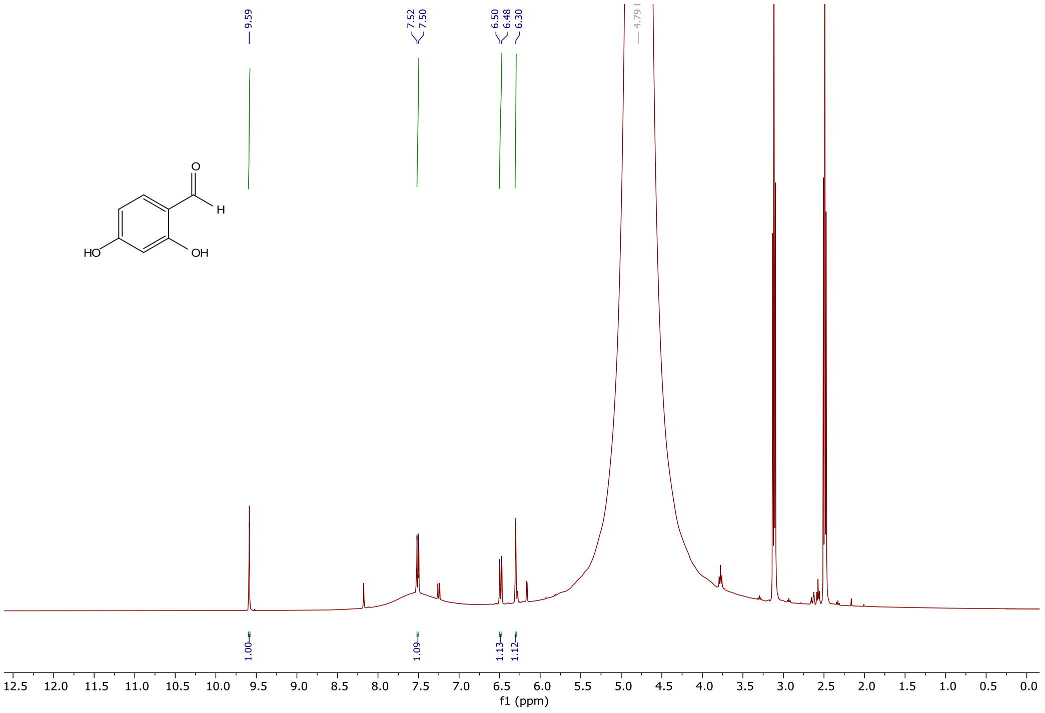

^{1}H NMR of 2,4 dihydroxybenzaldehyde Schiff Base (imine)

(400 MHz, D_2O) δ 8.18 (s, 1H), 7.25 (d, *J* = 8.8 Hz, 1H), 6.28 (d, 1H), 6.16 (d, 1H).

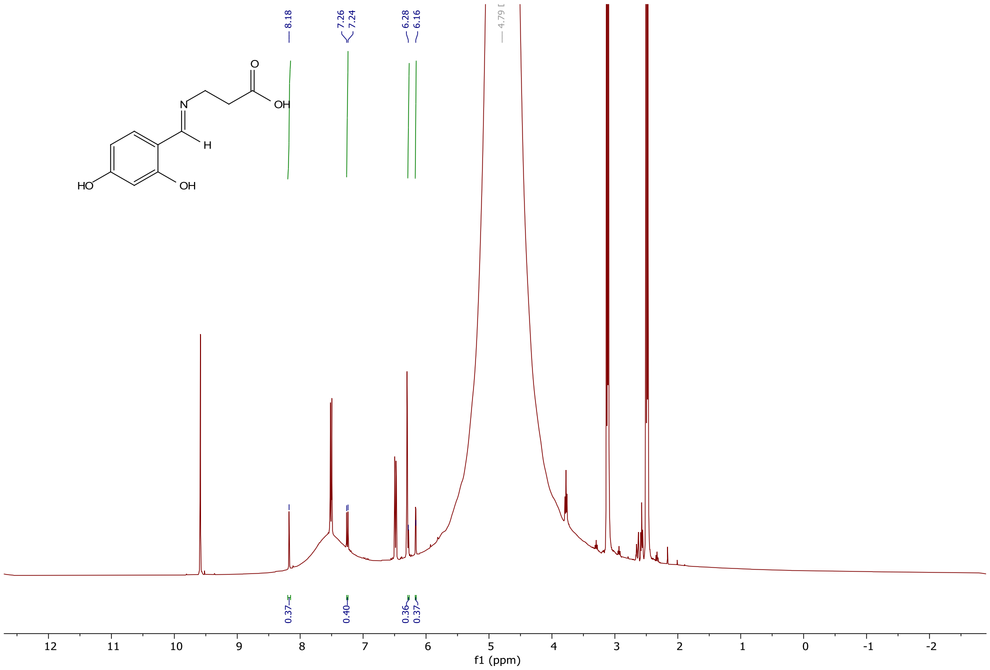

^{1}H NMR of 2,4 dihydroxybenzaldehyde and beta-alanine in pH 7.4 NaP buffer (28% imine)

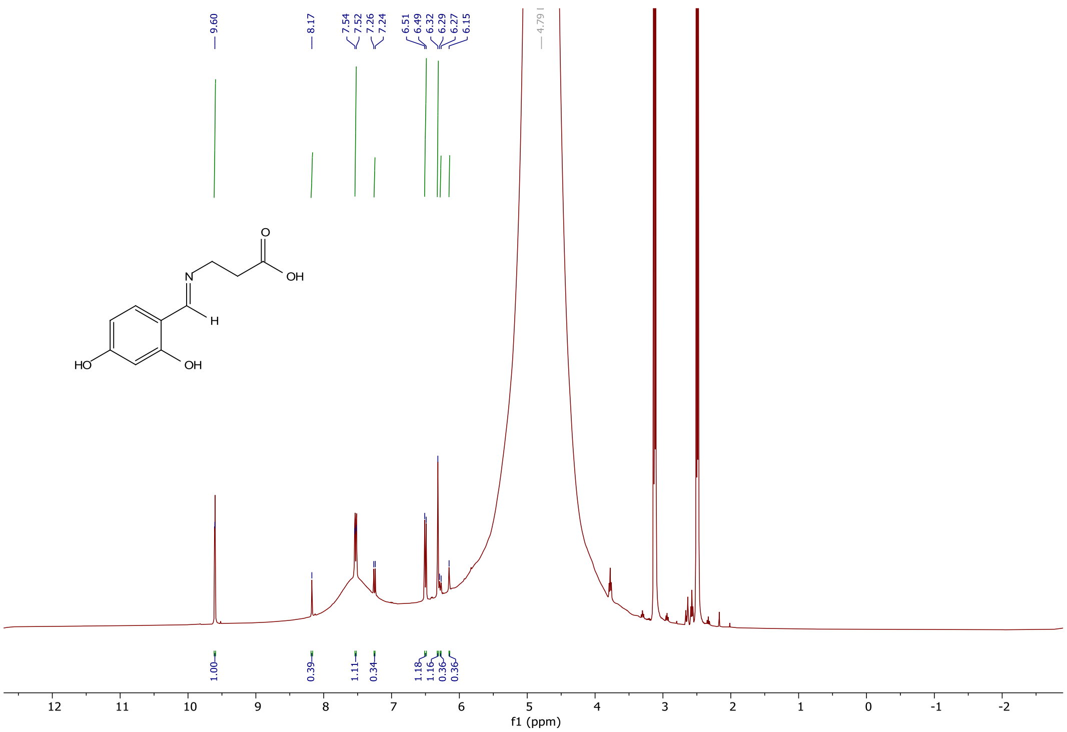

^{1}H NMR of 2,4 dihydroxybenzaldehyde and beta-alanine in pH 9 NaP buffer (39% imine)

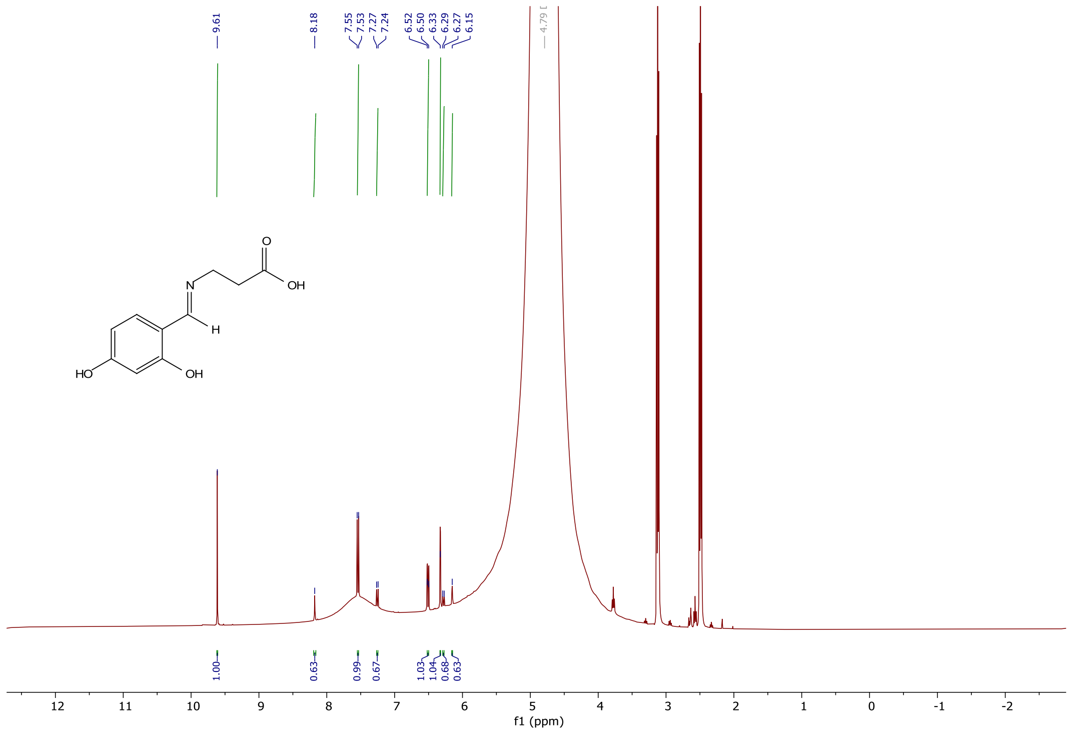

^{1}H NMR of 2,4 dihydroxybenzaldehyde and beta-alanine in pH 12 NaP buffer (42% imine).

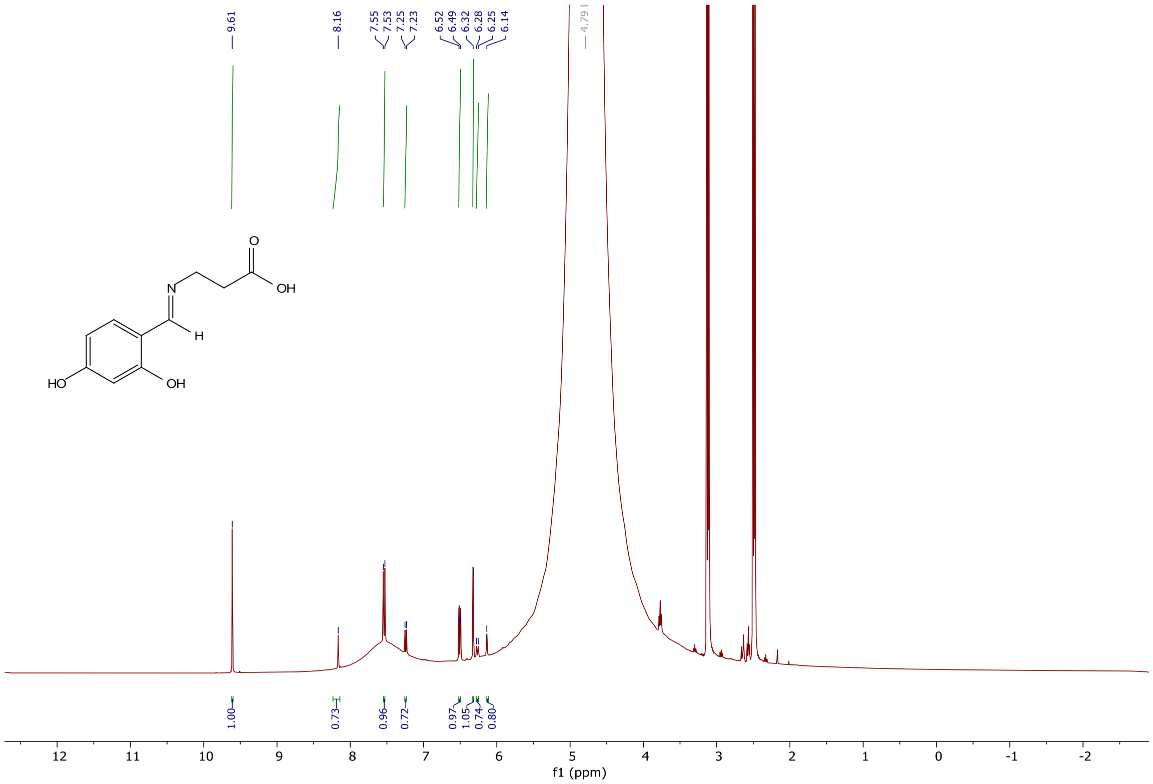

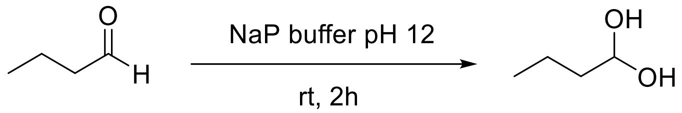

Procedure: Butyraldehyde (10 µL, 0.11 mmol) was dissolved in 600 µL of pH 12 20mM Phosphate buffer and stirred for 2h at room temperature. pH 12 20 mM phosphate buffer was prepared by dissolving 40 mg of dibasic sodium phosphate and 7 mg of monobasic sodium phosphate in 10 mL of D_2O and adding 0.8M NaOH until desired pH was acquired.

^{1}H NMR of Butyraldehyde in DMSO-d6.

^{1}H NMR (400 MHz, DMSO) δ 9.65 (t, *J* = 1.7 Hz, 1H), 2.39 (td, *J* = 7.1, 1.6 Hz, 2H), 1.54 (h, *J* = 7.3 Hz, 2H), 0.87 (t, *J* = 7.5 Hz, 3H).

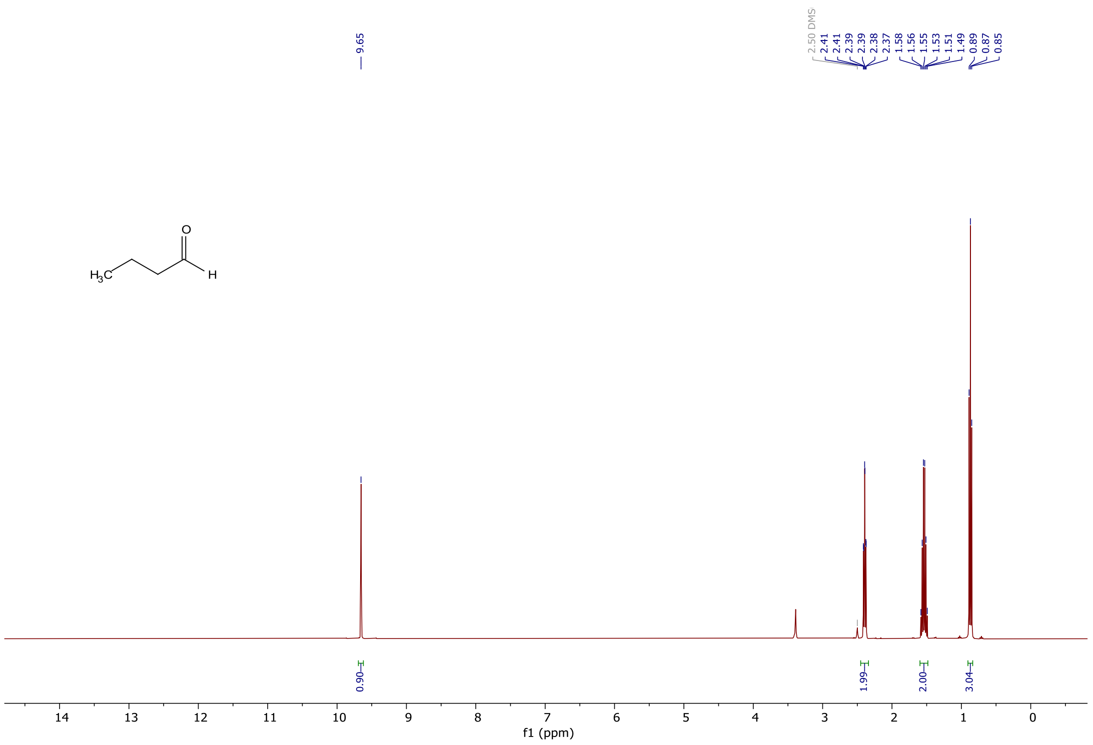

Butyraldehyde Hydrate in pH 12 NaP buffer in D_2O (50% hydrate).

^{1}H NMR (400 MHz, D_2O) δ 5.01 (t, *J* = 5.7 Hz, 1H), 1.65 – 1.57 (m, 2H), 1.41 – 1.29 (m, 2H), 0.89 – 0.86 (m, 3H).

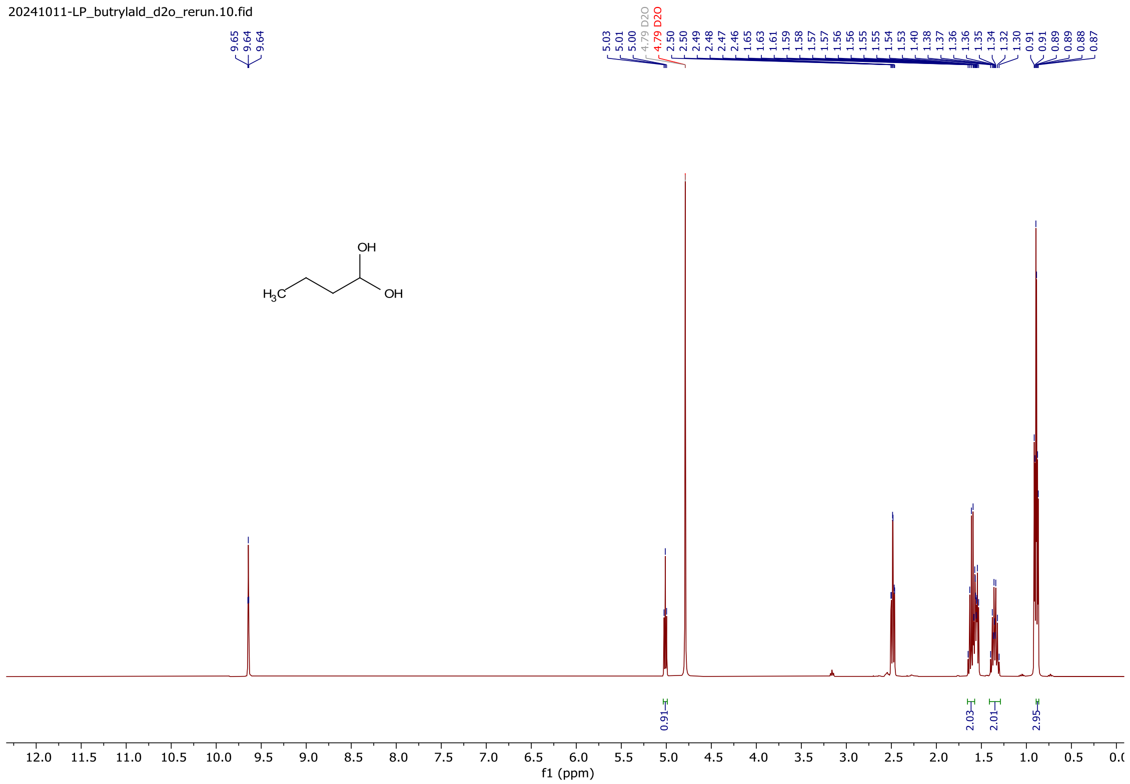

Figure S11. Arginine carbonylation of proteins and hydroxylamine fluorophore labeling

After modification of proteins using **General Procedure** for protein modification, reaction was neutralized to pH 5 with 1M HCl solution and purified with molecular weight cutoff. Modified proteins were resuspended in 500 µL of water. 200 µL of this solution was added to an aluminum foil wrapped vial, followed by the addition of 50 µL of 100 mM HA-647 fluorophore. Reaction was stirred for 3 h at room temperature. The reaction was stirred for 3 h, followed by analysis of proteins through in gel fluorescence imaging and Coomassie blue staining. Samples were loaded on a Novex WedgeWell 4-20% Tris-Glycine gel. Gel was run in Tris-glycine running buffer at 180V. The gel was then stained with Coomassie brilliant blue for 1 h and destained overnight.

Modification of Myoglobin (equine heart) with 2b:

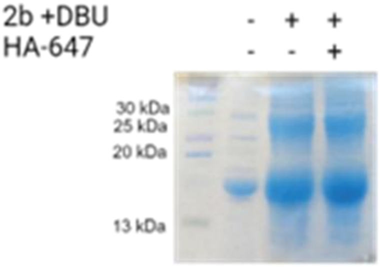

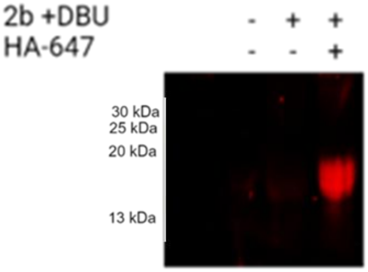

Modification of Lysozyme (human) with 2b:

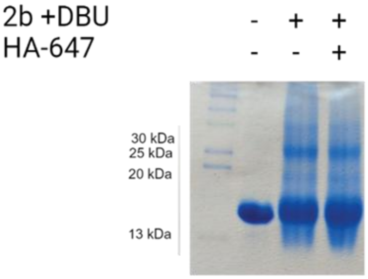

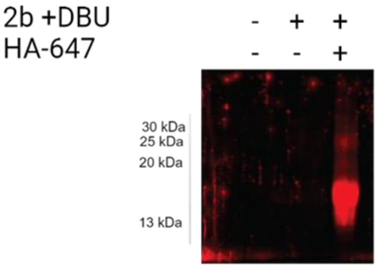

Modification of Lysozyme (chicken) with 2b:

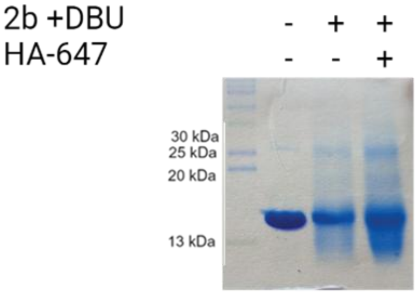

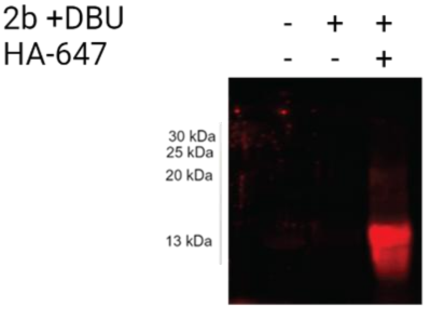

Figure S12. Arginine carbonylation of cell lysate and hydroxylamine fluorophore labeling

Cell Culture and Drugs. Cells were maintained at 37 °C and 5% CO_2. T-47D cells were cultured in RPMI supplemented with 10% (V/V) fetal bovine serum (FBS) and 1% (V/V) penicillin/streptomycin (100 µg/mL).

Cell Lysis. Whole cell lysate was generated by lysing cells on ice in RIPA buffer (50 mM TrisHCl [pH 8], 150 mM NaCl, 1% NP-40, 0.5% sodium deoxycholate, 0.1% SDS) supplemented with protease and phosphatase inhibitors. Lysates were centrifuged 6,500 x g, 10 m at 4°C, and soluble lysate was collected. Whole cell lysate proteins were separated using 16% SDS-PAGE. SDS-PAGE gels were stained with Coomassie brilliant blue dye.

Dose-dependent NORA modification of lysates and conjugation with hydroxylamine HA-647 fluorophore. To 4 tubes (individual reactions) of 100 µg of lysate in degassed MeCN:H_2O (1:4, 400 µL) were treated with freshly prepared 50 µM, 100 µM, 150 µM, and 200 µM of NORA reagents (probe **2b** and DBU) and stirred at 37 °C for 3 h. Upon completion of reaction, samples were acetone precipitated. Proteins were dissolved in 100 µL of PBS buffer, followed by the addition of 50 µL of 100 mM HA-647 fluorophore in water. The reaction was stirred for 3 h and acetone precipitated, followed by analysis of proteins through in gel fluorescence imaging and Coomassie blue staining. Samples were loaded on a Novex WedgeWell 4-20% Tris-Glycine gel. Gel was run in Tris-glycine running buffer at 180V. The gel was then stained with Coomassie brilliant blue for 1 h and destained overnight.

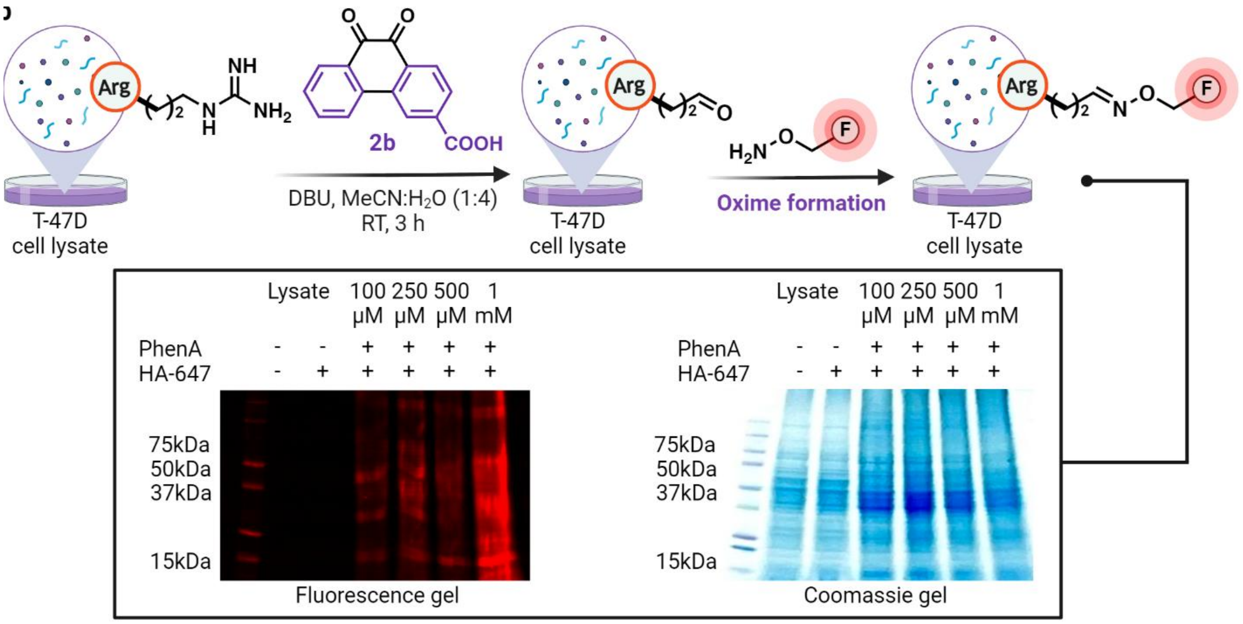

Figure S13. Cell Lysate Chemoproteomics Analysis of Arginine Carbonylation Reaction on Cell Lysate

Using the optimized reaction conditions describe above (Figure S12), whole cell lysate sample of 100 µg of lysate in degassed MeCN:H_2O (1:4, 400 µL) were treated with freshly prepared 50 µM, 100 µM, 150 µM, and 200 µM of NORA reagents (probe **2b** and DBU) and stirred at 37 °C for 3 h. Upon completion of reaction, samples were acetone precipitated and digested using SMART Digest™ GluC Kit by Thermo Scientific. This experiment was performed with (n = 2 biological samples). LC-MS/MS. Digested peptides were analyzed by LC/MS. Samples were analyzed by LC-MS using a Q-Exactive Plus orbitrap mass spectrometer equipped with Dionex UltiMate 3000 LC system (Thermo). Briefly, lyophilized digested peptides were resuspended in 0.1% formic acid in 10% acetonitrile and loaded onto a trap column (PepMap™ NEO 5 µm C18 300 µm X 5 mm Trap Cartridge) and resolved through a custom analytical column packed with ReproSil-Pur 120 C18-AQ 3 µm beads (Dr. Maisch GmbH) at a flow rate of 0.3 µL/min with a gradient solvent A (0.1% formic acid in 2% acetonitrile) and a gradient solvent B (0.1% formic acid in 80% acetonitrile) for 150 minutes. MS analysis was conducted in a data-dependent manner with full scans in the range from 400 to 1800 m/z using an Orbitrap mass analyzer set as follows: MS1: resolution = 70,000, AGC target = 3e6, Max IT = 100ms; MS2: resolution = 17,500, AGC target 1e5, Max IT = 50ms. The top fifteen most intense precursor ions were selected for MS2 with an isolation window of 4 m/z. Isolated precursors were fragmented by high energy collisional dissociation (HCD) with normalized collision energy (NCE) of 27. LC-MS RAW files were searched against the human proteome sequence database from UniProt using the Sequest HT search engine embedded in Proteome Discoverer 3.0 (Thermo) with 10 ppm MS1 precursor mass tolerance, 0.02 Da MS2 fragment mass tolerance, 0.01 false discovery rate. Search included the following modifications: Methionine oxidation (+15.99492 Da), asaparagine and glutamine deamidation (+0.98402 Da) and protein N-terminal acetylation (+42.03670), arginine modification (-43.0535) (up to 3 allowed per peptide); cysteine was assigned a fixed carbamidomethyl modification (+57.021465 Da).

Excel sheet of analysis is attached as supplementary data.

Metal-catalyzed oxidation (MCO) reactions of proteins.

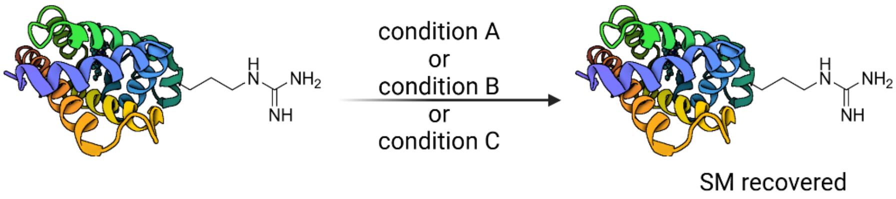

Procedure for carbonylation of myoglobin (condition A): Myoglobin (2 mg) was dissolved in 1000 µL Phosphate buffer solution (10 mM, pH 6.3). $MgCl_2$ (10 mM), KCl (90 mM), $FeSO_4$ (4 mM), and EDTA (8 mM) were added to reaction. Reaction was incubated overnight at 37 °C. Excess reagents were removed by purification with Amicron 3000 kDa molecular weight cutoffs

filters. Modified proteins were resuspended in 0.1% formic acid in water and analyzed by LCMS. No carbonylation of myoglobin was observed.

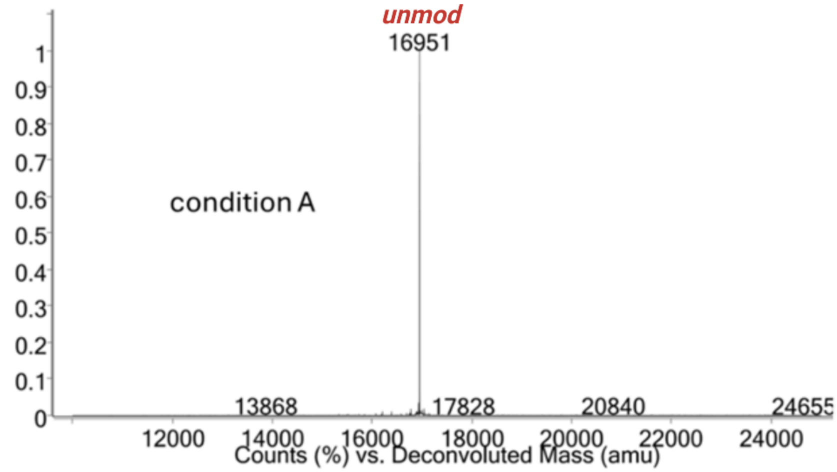

Procedure for carbonylation of myoglobin (condition B): Myoglobin (2 mg) was dissolved in 1000 µL Phosphate buffer solution (10 mM, pH 6.3). H_2O_2 (20 mM) and $FeSO_4$ (20 mM) were added to reaction. Reaction was incubated overnight at 37 °C. Excess reagents were removed by purification with Amicron 3000 kDa molecular weight cutoffs filters. Modified proteins were resuspended in 0.1% formic acid in water and analyzed by LCMS. No carbonylation of myoglobin was observed.

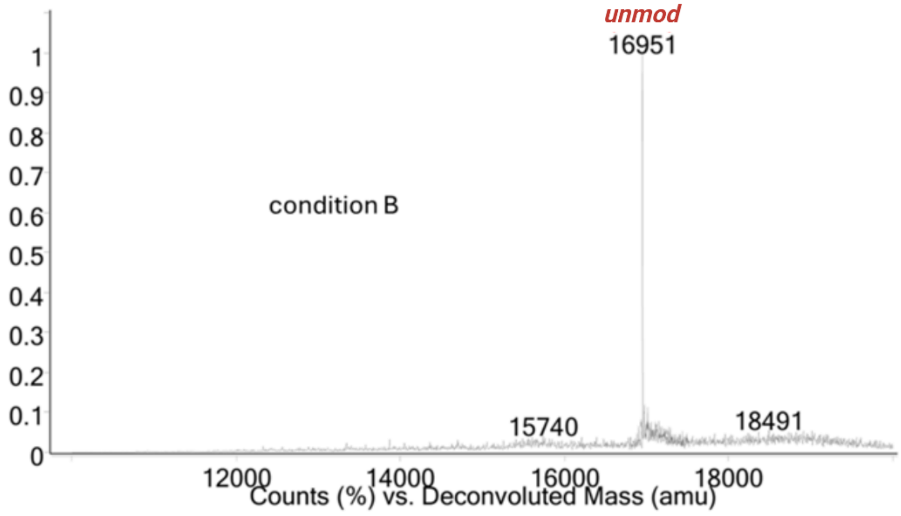

Procedure for carbonylation of myoglobin (condition C): Myoglobin (2 mg) was dissolved in 1000 µL Phosphate buffer solution (10 mM, pH 6.3). H_2O_2 (20 mM), $CuSO_4$ (20 mM), and 3-mercaptopriopionic acid (80 mM) were added to the reaction. Reaction was incubated overnight at 37 °C. Excess reagents were removed by purification with Amicron 3000 kDa molecular weight cutoffs filters. Modified proteins were resuspended in 0.1% formic acid in water and analyzed by LCMS. No carbonylation of myoglobin was observed.

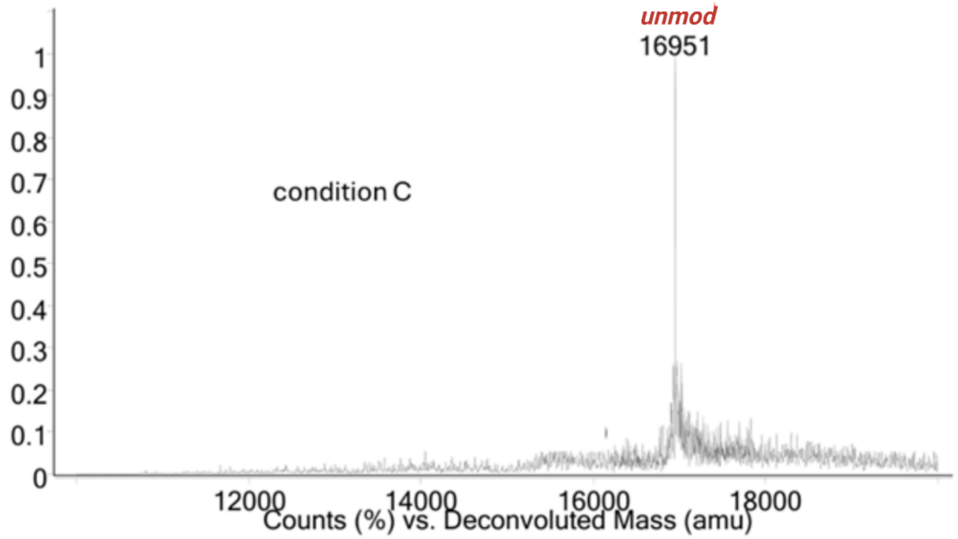

Figure S14. Sequence Motif analysis Sequence motif of modified arginine sites.

To identify the sequence motif of modified arginine sites, NORA modified arginine sites were utilized. Sequences containing 4 residues from the left and 4 residues from the right of modified arginine sites were utilized, with arginine as the fixed positions with a p-value <0.05. Sequence motif was generated using "probability logo generator for biological sequence motif" plogo v1.2.0

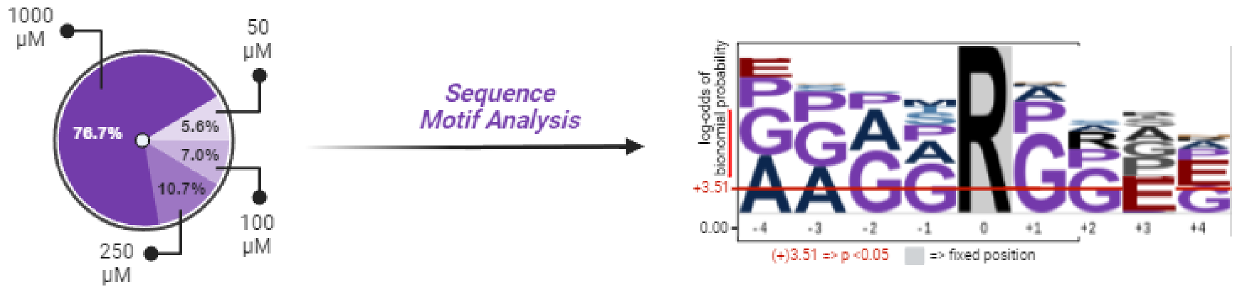

Figure S15. Gene Ontology (GO) analysis of hyperreactive arginine sites.

To evaluate the biological processes and localization of arginine modified proteins, Gene ID of NORA modified protein targets were extracted followed by GO analysis using ShinyGO 0.77. FDR cut-off was set at 0.05%.

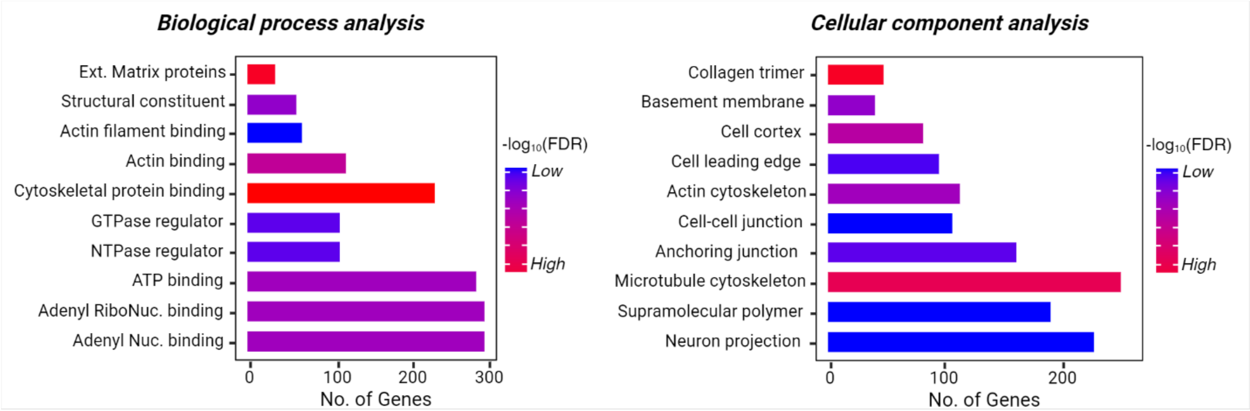

Figure S16. Confocal microscopy imaging of carbonylated T47D cells.

Live T-47D cells were plated on microscope slides in a 6 cm petri dish supplemented with RPMI media and incubated for 24 h. Cells were washed with PBS (3x) and fixed with 4% formaldehyde solution for 10 min. Cells were subsequently washed 3 times with PBS (5 min) and permeabilized with freshly prepared 0.1% Triton-X solution in PBS. Fixed cells were washed with PBS (3x) and treated with **2b** (250 µM and 1 mM), DBU (250 µM and 1 mM) for 3 h. After 3 h, cells were washed 3 times with cold PBS and labeled with hydroxylamine-647 fluorophore for 3 h. The reaction was stirred for 2 h and washed 3 times with PBS. Nuclear staining of cells was done with Fluoroshield-DAPI mounting media and subsequently imaged on a Leica SP8 confocal microscope. The images were processed and analyzed using ImageJ software to determine relative labelling of cells. For quantification >20 cells were used for control and experimental samples. A z-stack image of median intensity image from 22 slices of NORA modified cells are reported below. This experiment was repeated (n= 2 biological samples) with similar results.

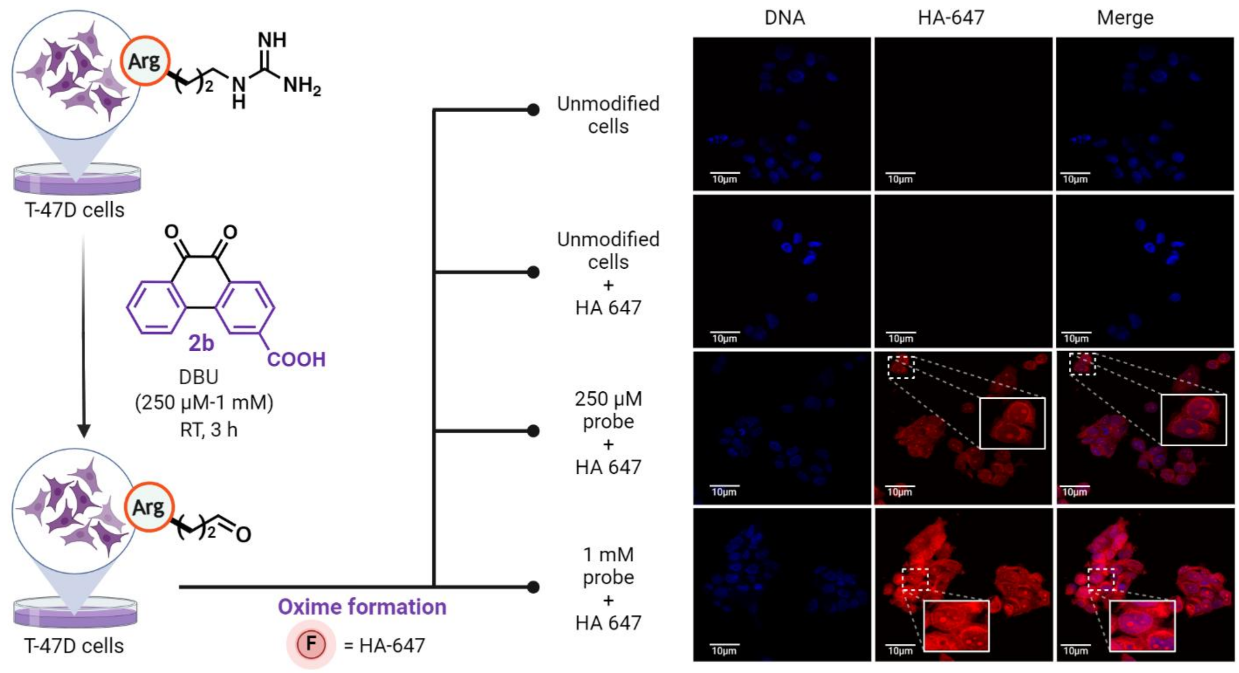

Figure. S17. Carbonylation mediated installation of post-translational modifications

Conversion of Aldehyde to Ornithine:

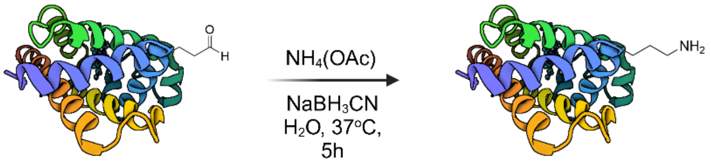

General Procedure: Myoglobin with single modification to aldehyde (1 mg, 118 µM) and ammonium acetate (4 mg, 104 mM) was dissolved in 125 µL of 2.0 M $NaBH_3CN$ solution in H_2O. Reaction was diluted to 0.5 mL with H_2O and stirred at 37 °C for 5 h. Reaction was purified using Sigma 3000 kDa molecular weight cutoffs. Modified protein was resuspended in 0.1% formic acid in water and analyzed by LCMS. Products are listed as modified. The conversion was found to be >95%.

Starting Aldehyde:

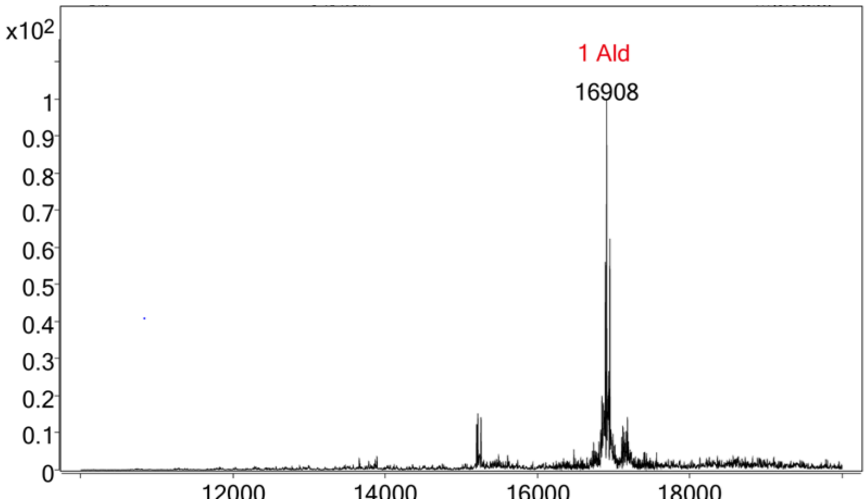

Ornithine:

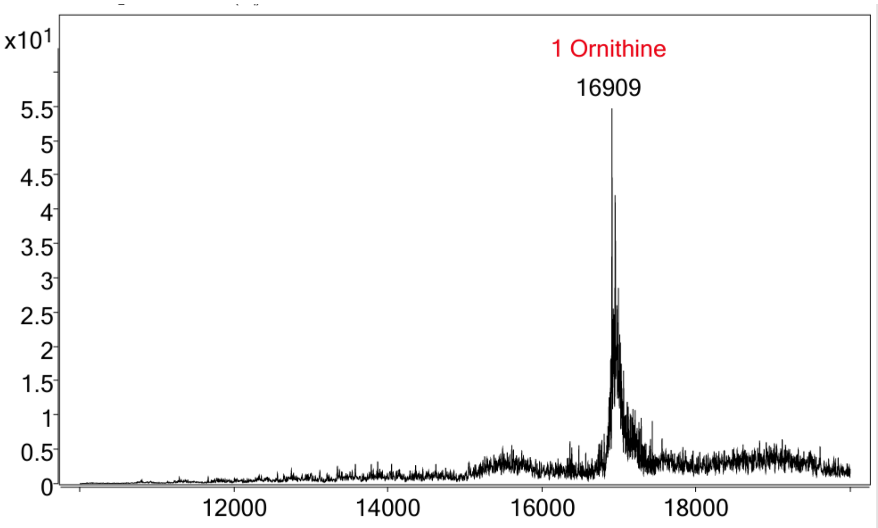

Conversion of Aldehyde to Monomethylornithine

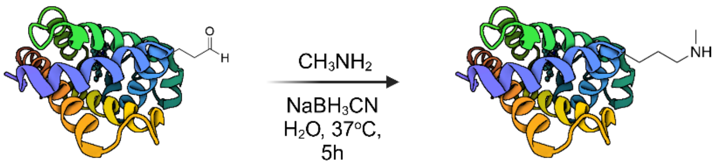

General Procedure: Myoglobin with single modification to aldehyde **(**1 mg, 118 µM) and methylamine solution in THF (4 µL, 16 mM) was dissolved in 125 µL of 2.0 M $NaBH_3CN$ solution in H_2O. Reaction was diluted to 0.5 mL with H_2O and stirred at 37 °C for 5 h. Reaction was purified

using Sigma 3000 kDa molecular weight cutoffs. Modified protein was resuspended in 0.1% formic acid in water and analyzed by LCMS. Products are listed as modified. The conversion was found to be ~56%.

Starting Aldehyde:

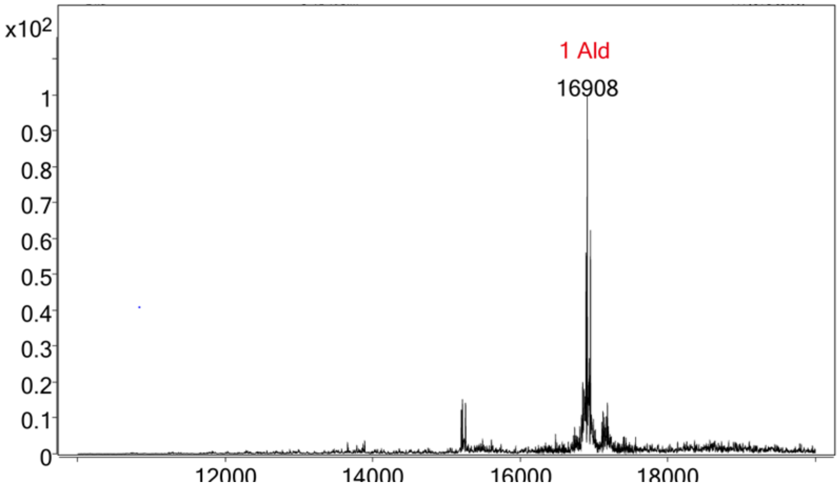

Monomethylornithine:

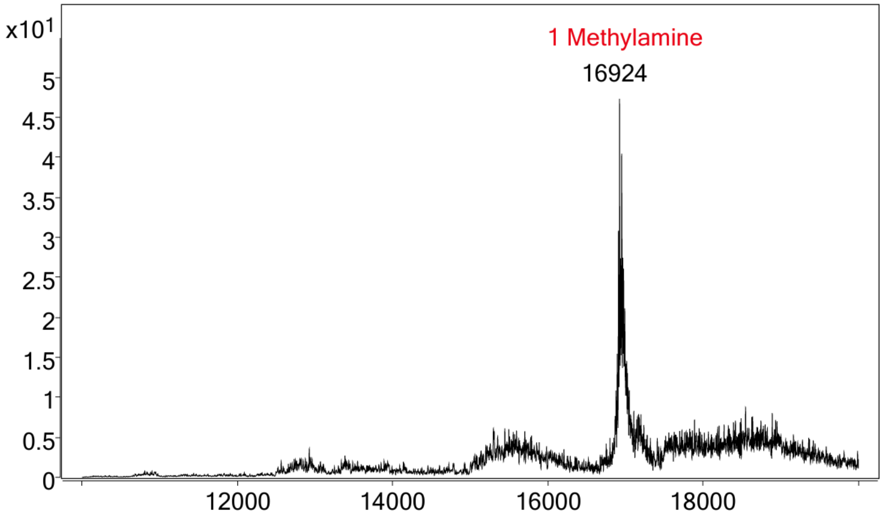

Conversion of Aldehyde to N-Methyl propargyl ornithine:

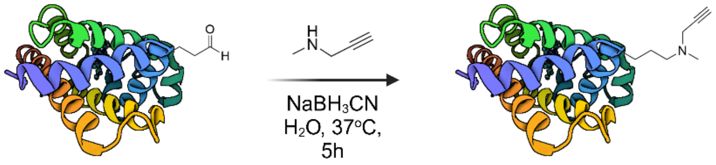

General Procedure: Myoglobin with single modification to aldehyde (1 mg, 118 µM) and *N*-methylpropargylamine (4 µL, 95 mM) was dissolved in 125 µL of 2.0 M $NaBH_3CN$ solution in

H_2O. Reaction was diluted to 0.5 mL with H_2O and stirred at 37 °C for 5 h. Reaction was purified using Sigma 3000 kDa molecular weight cutoffs. Modified protein was resuspended in 0.1% formic acid in water and analyzed by LCMS. Products are listed as modified. The conversion was found to be ~61%.

Starting Aldehyde:

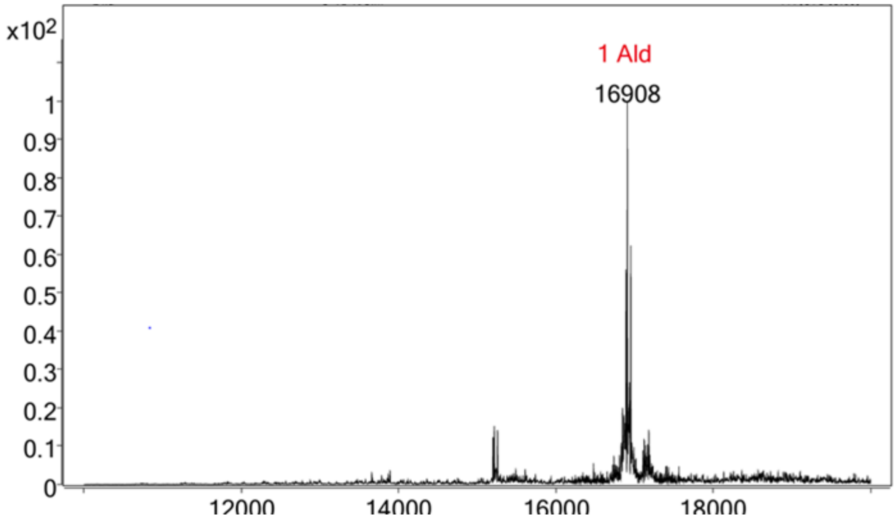

N-Methyl propargyl ornithine:

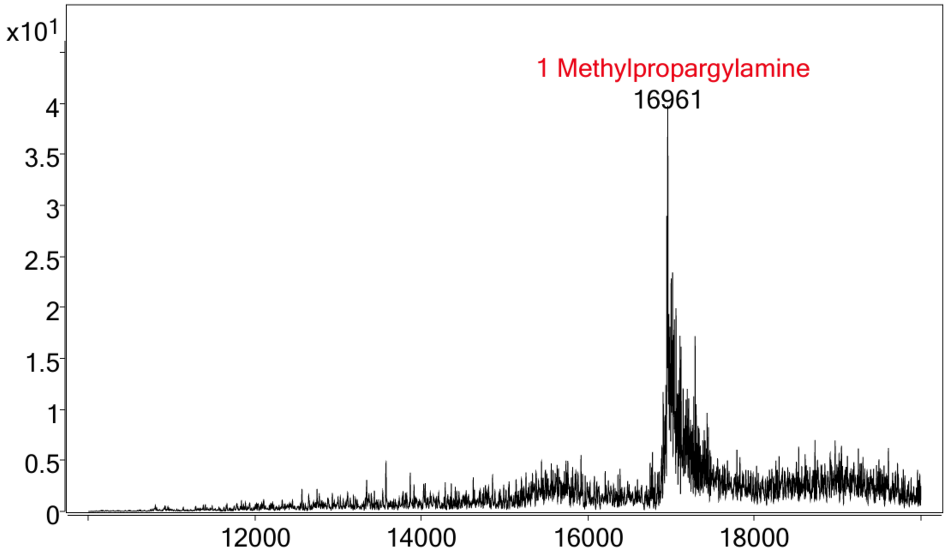

Conversion of Aldehyde to Diethyl ornithine:

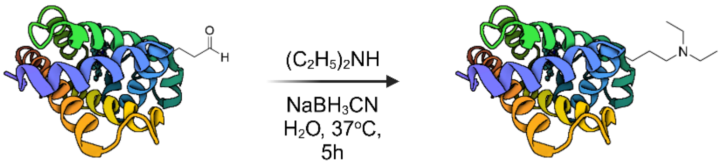

General Procedure: Myoglobin with single modification to aldehyde (1 mg, 118 µM) and diethylamine (1 µL, 16 mM) was dissolved in 125 µL of 2.0 M $NaBH_3CN$ solution in H_2O. Reaction was diluted to 0.5 mL with H_2O and stirred at 37 °C for 5 h. Reaction was purified using Sigma 3000 kDa molecular weight cutoffs. Modified protein was resuspended in 0.1% formic acid in water and analyzed by LCMS. Products are listed as modified. The conversion was found to be ~70%.

Starting Aldehyde:

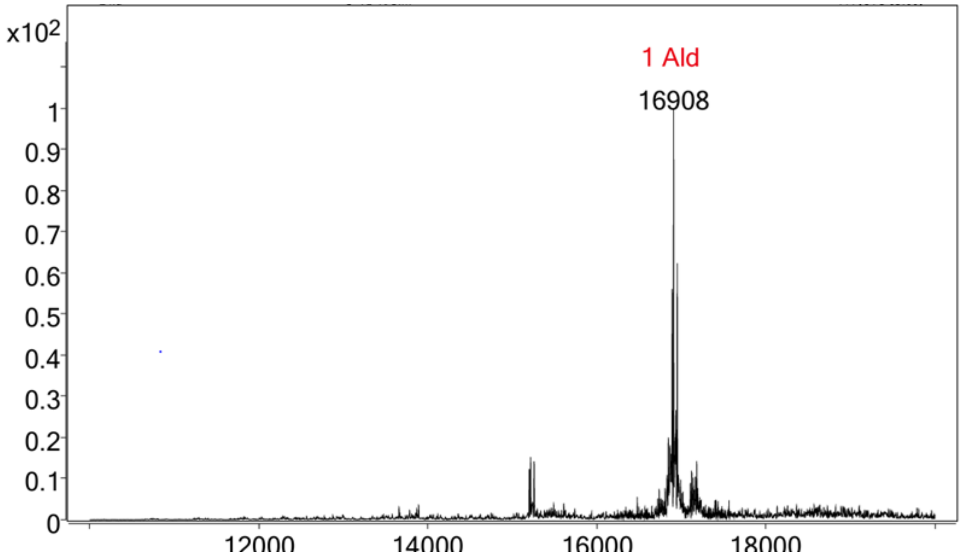

Diethylornithine Product:

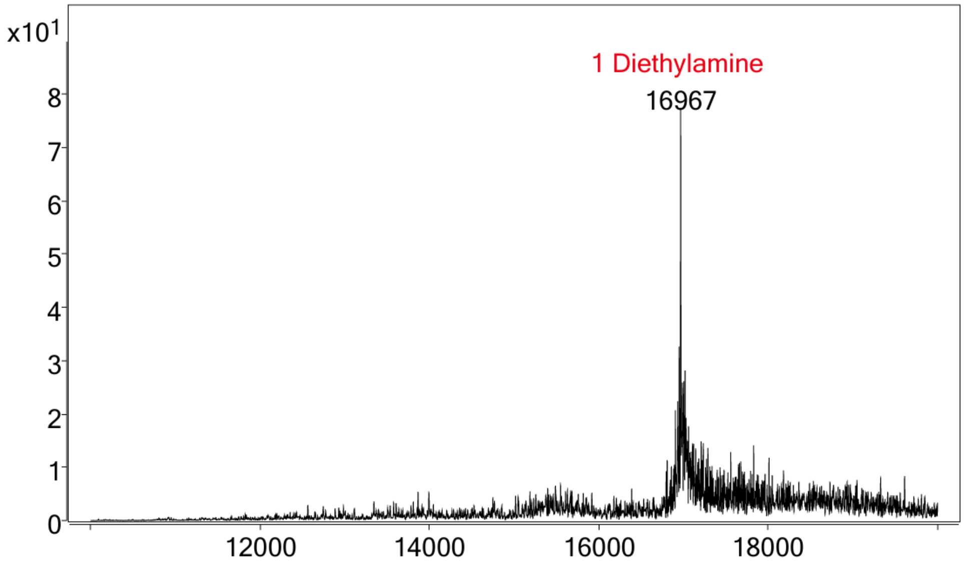

Conversion of Aldehyde to Dimethylornithine

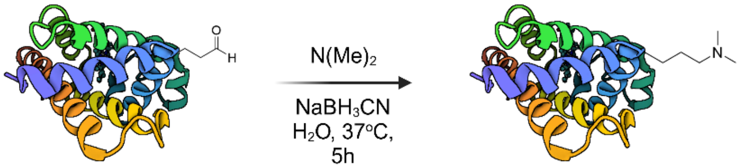

General Procedure: Myoglobin with single modification to aldehyde (1 mg, 118 µM) and *N*,*N*-dimethylamine solution in THF(4 µL, 16 mM) was dissolved in 125 µL of 2.0 M $NaBH_3CN$ solution in H_2O. Reaction was diluted to 0.5 mL with H_2O and stirred at 37 °C for 5 h. Reaction was purified using Sigma 3000 kDa molecular weight cutoffs. Modified protein was resuspended in 0.1% formic acid in water and analyzed by LCMS. Products are listed as modified. The conversion was found to be ~50%

Starting Aldehyde:

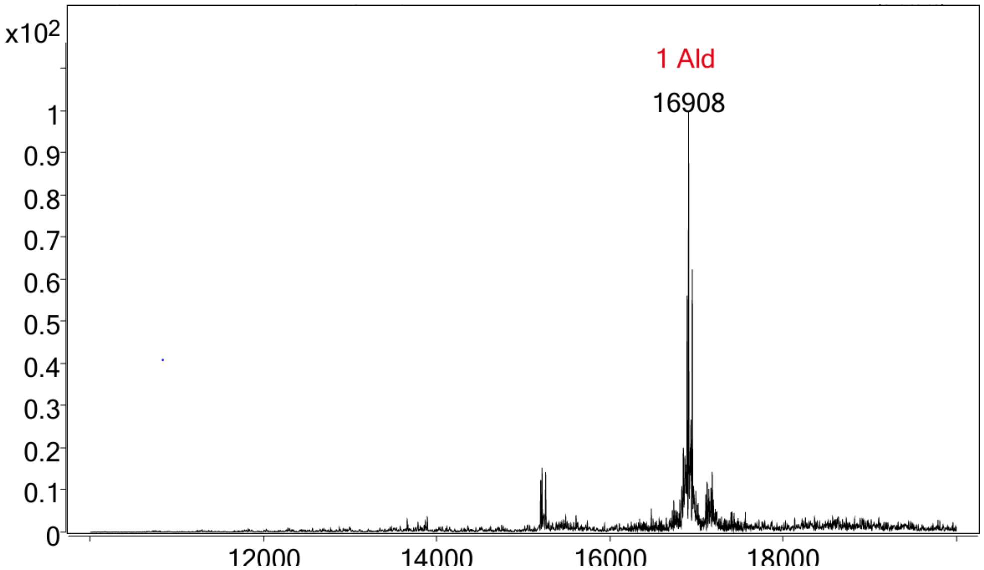

Dimethylornithine Product:

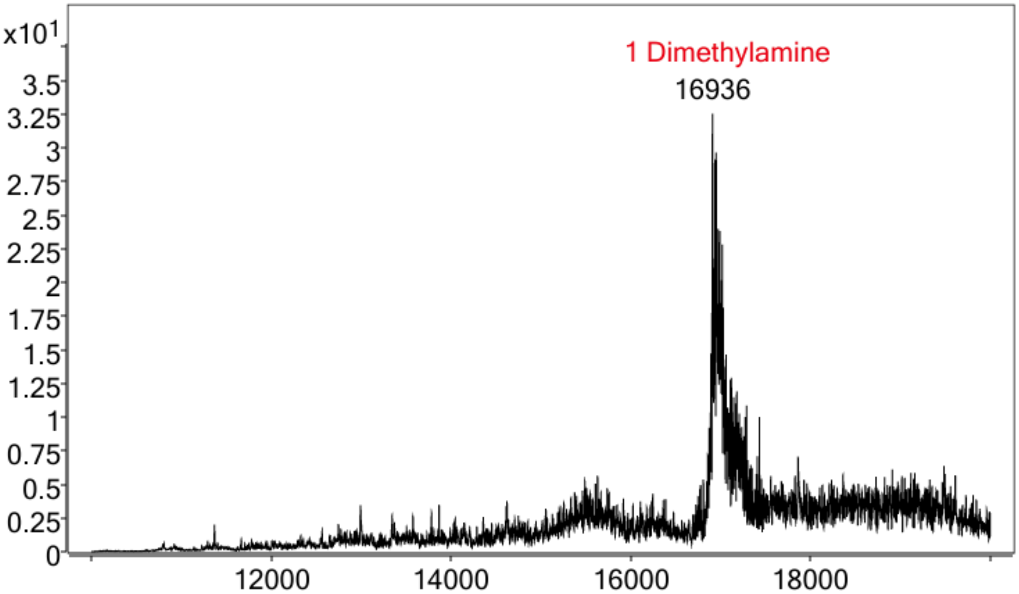

References.

1. Chan, W. C.; White, P. D. Fmoc solid phase peptide synthesis: A practical approach (Oxford Univ. Press, New York, 2000).
